# Multicolor Digital Light Processing 3D Printing Enables Dissolvable Supports for Freestanding and Non-Assembly Structures

**DOI:** 10.1021/acscentsci.5c00289

**Published:** 2025-05-29

**Authors:** Keldy S. Mason, Ji-Won Kim, Elizabeth A. Recker, Jenna M. Nymick, Mingyu Shi, Franz A. Stolpen, Jaechul Ju, Zachariah A. Page

**Affiliations:** † Department of Chemistry, 12330 The University of Texas at Austin, Austin, Texas 78712, United States; ‡ McKetta Department of Chemical Engineering, 12330 The University of Texas at Austin, Austin, Texas 78712, United States

## Abstract

The limited diversity in photocurable resin chemistries has precluded access to certain geometries using digital light processing (DLP) 3D printing, a rapid, precise, economical, and low-waste manufacturing technology. Specifically, freestanding structures with floating overhangs (e.g., hooks) and mobile nonassembly structures that cannot be physically separated (e.g., joints) represent two such geometries that are difficult or impossible to access with contemporary DLP 3D printing. Herein, we disclose novel resins that selectively react with different colors of light to form soluble thermoplastics and insoluble thermosets. Systematic characterization of the acrylate- and epoxy-based resins and corresponding polymers from simultaneous UV and visible (violet or blue) light exposure revealed a rapid multimaterial 3D printing process (∼0.75 mm/min) capable of providing supports that dissolve in ethyl acetate, a “green” solvent, within 10 min at room temperature. Relative to manual support removal, the present process provides comparable or improved surface finishes and higher throughput. Finally, several proof-of-concept structures requiring dissolvable supports were 3D printed, including hooks, chains, and joints, which were scanned using computed tomography to showcase the process’s geometric versatility and high fidelity. This work provides fundamental design principles for multimaterial resin chemistry and lays a foundation for automating next generation additive manufacturing.

## Graphical abstract

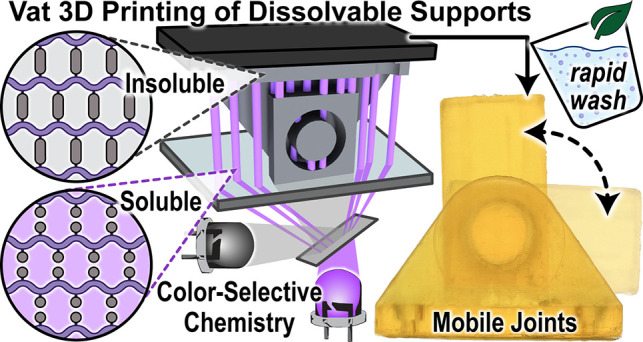

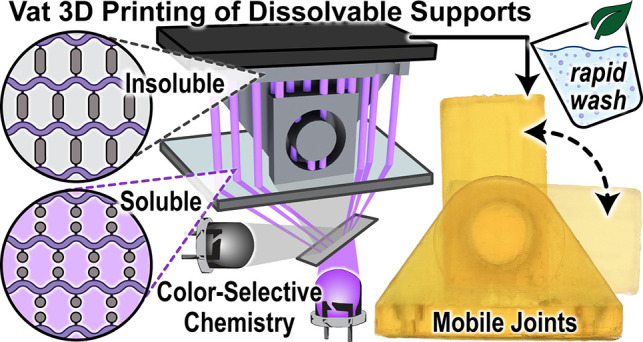

## Introduction

Additive manufacturing (AM) has emerged as a pillar for rapid and material conservative fabrication of objects with complex geometries, from consumer goods (e.g., toys, shoes, and jewelry)^1^ to medical devices (e.g., prosthetics^2^ and dental aligners^3^) and robotics (e.g., joints and actuators).^4–6^ Among the various AM methods, digital light processing (DLP), where liquid resin is rapidly cured into a solid polymer upon exposure to light in a layer-by-layer fashion, provides exceptional precision and throughput at a low cost.^7–9^ However, several hurdles have prevented widespread adoption of DLP and vat photopolymerization in general, particularly related to automation and the need for supports to access freestanding and nonassembly structures.^10^ Such structures currently require manual postprocessing steps, including labor intensive support removal and surface finishing (Figure 1A), which can account for ∼10% of the total AM cost.^11,12^ To effectively address these issues requires innovative resin chemistries and curing processes.

**Figure 1:**
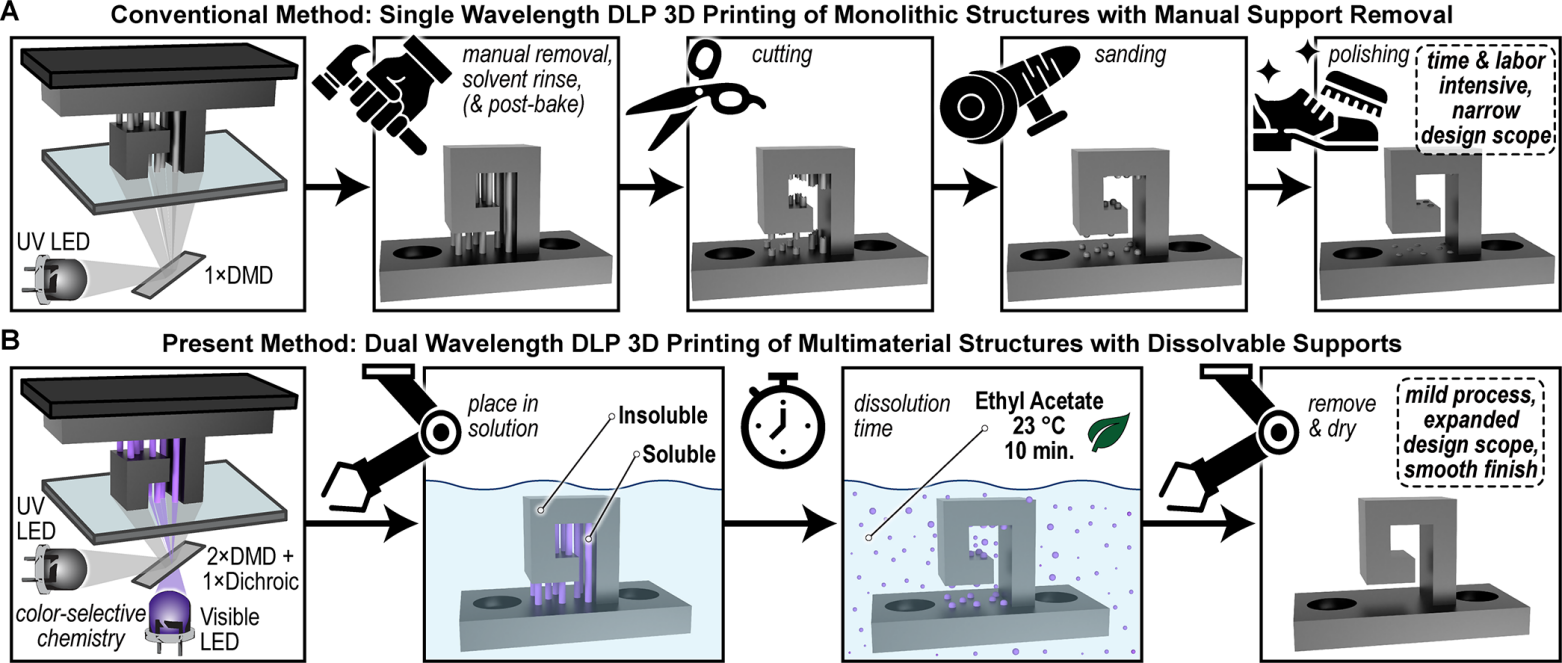
Illustrations of a hook geometry containing the necessary supports for preparation via digital light processing (DLP) 3D printing and associated postprocesses governed by the resin chemistry employed. (A) Contemporary method where a UV light curable resin results in the formation of a monolithic structure that requires laborious manual support removal. (B) Present strategy where a wavelength-selective resin results in the formation of a multimaterial structure with dissolvable supports for ease of postprocessing via solvent washing.

Supports are vital to many AM processes to stabilize parts during fabrication and prevent distortions. This is especially true for components with large overhangs, limited build platform contact, or structures demanding high accuracy and precision.^13–15^ Furthermore, joint structures fabricated using AM present unique challenges, yet the versatility of motion that they impart has motivated clever solutions to their production.^16^ Specialized designs to enable assembly postproduction, easy to remove supports, or alternative nonassembly geometries that provide similar functionality (i.e., movement) have been developed.^5,17,18^ Unfortunately, these methods exacerbate production cost and time, while also limiting access to joints that provide smooth motion owing to surface blemishes from residual support material.^5^ Contemporary multimaterial AM has offered a unique approach to this problem, where a dissolvable material is deposited as the support structure, followed by simple solvent washing for removal.

Exemplary multimaterial AM technologies that have introduced dissolvable supports include inkjet (e.g., material and binder jetting) and extrusion (e.g., fused deposition modeling).^19–24^ These processes rely on disparate physical properties (e.g., solubility or melting point) of the support material relative to those making up the desired product. While multimaterial jetting offers high feature resolution and fast build speeds with multiple printheads, the printers are costly. In contrast, multimaterial extrusion printers can be inexpensive, but build times are long, large flushing volumes during filament exchange leads to considerable waste, and feature resolution is limited.^20,25^ Multimaterial vat photopolymerization has the potential to combine high resolution, minimal waste, and low cost. However, multimaterial DLP from a single vat remains a nascent area of research that has yet to demonstrate the production of dissolvable supports, although the concept has been proposed in the patent literature^26–128^ and demonstrated using a vat-exchange process. Specifically, multicolor DLP 3D printing offers a foundation for spatially programming material properties, which has enabled the integration of disparate mechanical, optical, and dynamic chemical profiles.^129–34^

Inspired by these prior efforts, we developed novel resins that upon exposure to ultraviolet or visible light selectively produced an insoluble thermoset or soluble thermoplastic, respectively (Figure 1B). Systematic characterization of photosystem components enabled the requisite selectivity to provide disparate solubility without additional postprocessing steps (e.g., thermal treatment) prior to support removal via fast dissolution. As a proof-of-concept, these resins were applied to multicolor DLP 3D printing to produce smooth, freestanding and nonassembly structures (e.g., joints) that would otherwise be difficult (or impossible) to produce via vat photopolymerization. This work represents a rapid and potentially automated approach for support removal from DLP 3D printed objects, which can reduce cost and increase throughput by removing manual intervention while also facilitating the production of structures useful in medicine and robotics.

## Results and Discussion

Key monomers in the multimaterial resins were isobornyl acrylate (IBOA, ∼58 wt %) to provide the soluble thermoplastic via radical polymerization (previously demonstrated for single-component DLP 3D printing),^35,36^ 3,4-epoxycyclohexylmethyl acrylate (ECA, ∼24 wt %) as a hybrid monomer to provide the insoluble thermoset via cationic epoxy cross-linking, and 2-hydroxyethyl acrylate (HEA, 12 wt %) to accelerate the cationic curing^31,37,38^ (Figure 2A and Tables S1–S3). The dual functionality of the hybrid monomer, ECA, was envisioned to facilitate (1) dilution of IBOA without compromising vitrification of the soluble support material required to print the thermoplastic and (2) thermomechanical stability of the insoluble material by covalently tying the otherwise orthogonal acrylate and epoxy networks together.

Two distinct photosystems were developed to selectively react under UV and violet light (photosystem 1) or UV and blue light (photosystem 2) (Figure 2B). The photosystems were designed to have 365 nm UV light trigger radical (acrylate) and cationic (epoxy) polymerizations, while visible light (405 or 460 nm) would induce radical-only polymerization. Commercially available diphenyl­[4-(phenylthiol)­phenyl]­sulfonium hexafluoroantimonate (DHS, 0.5 mol %) served as a common photoacid generator (PAG) in both photosystems. Photosensitizers were employed to accelerate the rate-limiting cationic curing process. Namely, 3,6-dimethoxy-9*H*-thioxanthen-9-one (MeOTX, 0.5 mol %),^39^ synthesized in-house (Scheme S1), and commercial isopropylthioxanthone (ITX, 0.5 mol %, mixture of 2- and 4-isomers), were used with photosystems 1 and 2, respectively. For the visible-light reactive radical photoinitiators phenylbis­(2,4,6-trimethylbenzoyl)­phosphine oxide (BAPO, 0.2 mol %) and bis­(4-methoxybenzoyl)­diethylgermanium (Ivocerin, 0.2 mol %) were incorporated into photosystems 1 and 2, respectively. Finally, ethyl 2-(tosyloxy)­acrylate (EVS, 2 mol %) was added to both resins as a rapid chain transfer agent, reducing the kinetic polymer chain length and network homogeneity to delay vitrification and increase dissolution of the thermoplastic.^40^

UV–vis absorption spectroscopy was conducted for each photosystem in the multimaterial (IBOA, ECA, HEA) resin to assess their spectral overlap with the LEDs used for DLP 3D printing (Figure 2C). Samples 50 μm thick (= one printed layer) were measured and absorption profiles were overlaid onto the relevant LED emission profiles. For both photosystems, the full resin absorption trace matched a linear sum of each component, indicating that there were no adverse ground state interactions. As indicated by the shaded regions in Figure 2C, MeOTX in photosystem 1 selectively absorbed only UV light, while BAPO effectively absorbed violet light. Similarly, in photosystem 2 ITX only absorbed UV light, while Ivocerin absorbed blue light. These results encouraged further wavelength-selective reactivity studies, leveraging the selective absorption of photosensitizers and initiators to drive distinct curing mechanisms.

Fourier transform infrared (FTIR) spectroscopy was used to monitor monomer conversion (ρ) during irradiation (Figure 2D and Section S2.1). Acrylate consumption was quantified by monitoring the disappearance of the C=C–H and C=C stretches at 3130 cm^–1^ and 1620 cm^–1^, respectively, while epoxide functionality could not be directly quantified in the multimaterial resin owing to overlapping IR absorption bands at ∼750 and 910 cm^–1^ (cyclic C–O–C vibrations).^41–44^ Instead, an acrylate-free proxy resin was developed to assess epoxide reactivity and wavelength-selectivity. The proxy resin contained 3,4-epoxycyclohexylmethyl 3,4-epoxycyclohexanecarboxylate (ECC, ∼56 wt %), a cycloaliphatic epoxide analogous to ECA, and 3-ethyl-3-oxetanemethanol (OXA, ∼41 wt %) to accelerate the cationic polymerization, similar to HEA (Figure S1 and Tables S4–S6). All resins were irradiated ten seconds after starting each FTIR measurement to demonstrate the temporal control required for 3D printing. Exposing resins containing photosystem 1 to violet light (405 nm, 50 mW/cm^2^) led to rapid acrylate conversion (952 ± < 1 mM/s) and a max conversion (ρ_max_) of 0.95 ± 0.01, while epoxy functionality did not react within the measurement time frame (2 min, Figure S8 and Table S8). The less than unity ρ_max_ for acrylate was attributed to vitrification of the glassy thermoplastic (*T*_g_ ≈ 60 °C by dynamic mechanical analysis, Figures S12–S14 and Tables S10–S12), a necessary feature for DLP 3D printing of the present dissolvable material. Irradiating photosystem 1 with UV light (365 nm, 50 mW/cm^2^) led to rapid conversion of both acrylate and epoxy functionality (1360 ± 20 mM/s and 450 ± 30 mM/s, respectively). Notably, ρ_max_ of the acrylate system was 0.90 ± 0.03, ∼5% lower than samples irradiated with violet light, which may be due to epoxy cross-linking restricting monomer mobility. However, the low epoxy ρ_max_ of 0.59 ± 0.01 may not be representative of the multicomponent, ECA-containing, resins, given potentially distinct gelation/vitrification kinetics. For resins containing photosystem 2, similar trends were observed, albeit with overall slower reaction kinetics for acrylate polymerization upon exposure to blue light (460 nm, 50 mW/cm^2^, 391 ± 6 mM/s) and epoxy polymerization upon exposure to UV light (365 nm, 50 mW/cm^2^, 80 ± 3 mM/s). Crucially, irradiating the multicomponent resin with UV light led to a rapid acrylate polymerization (2020 ± 90 mM/s) with a ρ_max_ of 0.88 ± 0.01.

**Figure 2:**
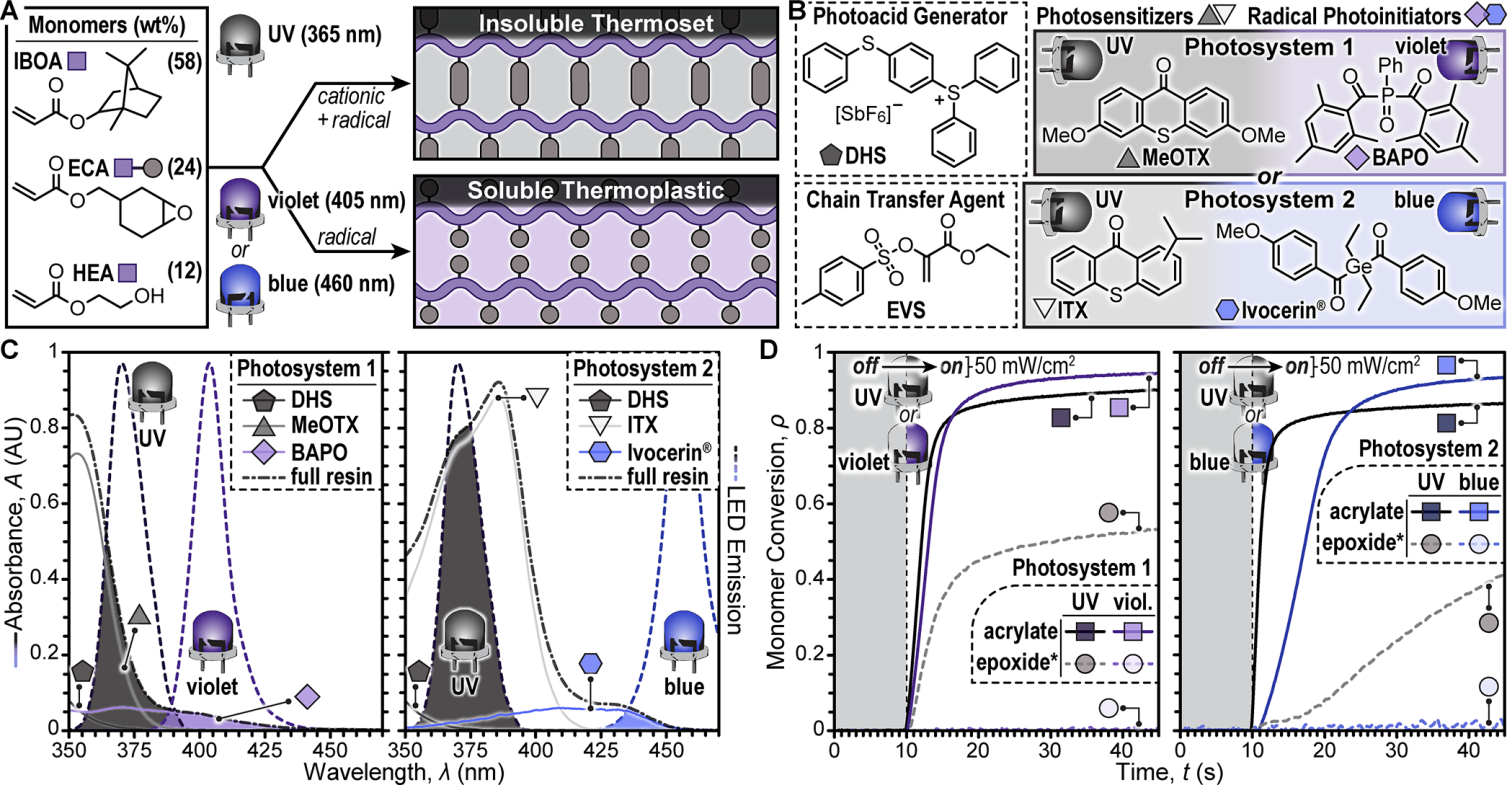
Resin formulation, photophysical characterization, and reaction kinetics. (A) Chemical structures for monomers and illustrations of networks from UV light (thermoset) or visible light (thermoplastic) exposure to provide disparate solubility. (B) Chemical structures of photosystem components for two distinct formulations. (C) UV–vis absorption spectra of the full resin and its individual components overlaid onto LED traces from the corresponding DLP 3D printer projections. Samples were 50 μm thick, and shaded regions represent overlap between full resin absorption and LED emission. (D) Monomer conversion vs time determined using Fourier transform infrared spectroscopy during irradiation with UV or visible light (50 mW/cm^2^). Samples were 50 μm thick. *Selective epoxide conversion was demonstrated using a proxy resin owing to convolution of the C–O–C absorption band in the ECA resin. The proxy resin comprised an analogous cycloaliphatic epoxide (ECC) and an oxetane-alcohol (OXA) without acrylate monomer (see SI for details). Traces are averages of three measurements.

Next, dissolution studies were conducted by applying these resins in DLP 3D printing and using ethyl acetate as a “green” solvent^45^ for washing. A light intensity of 50 mW/cm^2^ at the image plane for all three colors facilitated rapid printing, with 4 s (photosystem 1) or 6 s (photosystem 2) of exposure per 50 μm layer (build speed = 0.5–0.75 mm/min). Postprinting, all samples were rinsed with isopropyl alcohol, submerged in ethyl acetate at room temperature (∼23 °C) for an allotted time, and then rigorously dried in a vacuum oven (100 °C, 0.05 mmHg, 24 h) prior to testing, which ensured complete removal of solvent as confirmed using thermogravimetric analysis (Figure S15). As a simple representative object, UV light cured disks (14 mm diameter, 2 mm thick) with visible (violet or blue) light cured cylindrical supports (750 μm diameter) were prepared and examined for dissolution studies (Figure 3A, Movie S1, and Section S2.4 for experimental details). Gravimetric analysis revealed that only 10 min was required to fully dissolve the supports with mild agitation (shaking, 300 rpm), irrespective of the photosystem used, while the disks remained insoluble with little-to-no mass loss after ∼15 min of soaking (Figure 3B). Rheology confirmed dissolution of the support material, which displayed Newtonian liquid behavior in diglyme, a high boiling point proxy solvent (Figure S21).

**Figure 3:**
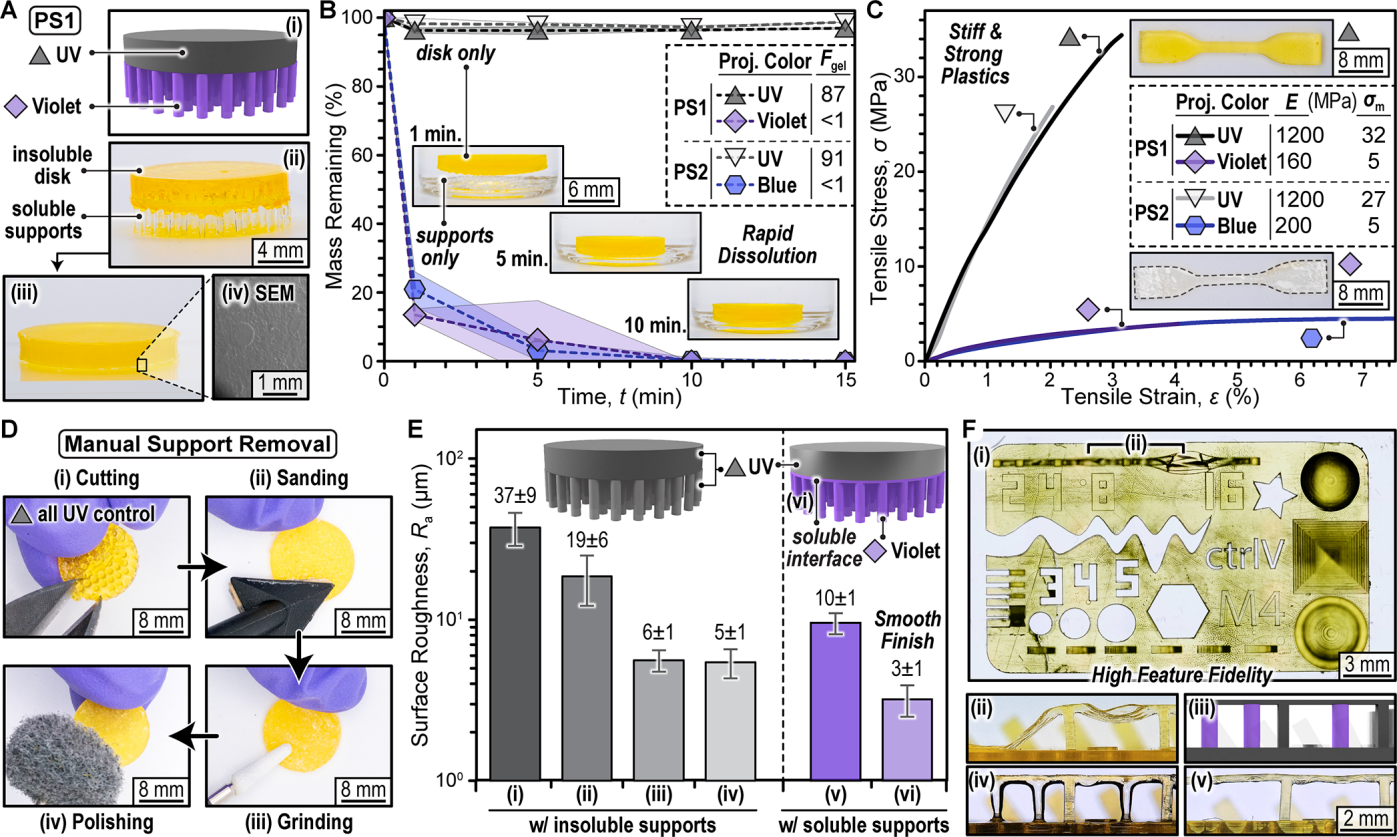
Dissolution kinetics, mechanical properties, and resolution of multimaterial prints. (A) Disk print with soluble supports showing (i) digital rendering, (ii) image of structure as printed and (iii) washed with ethyl acetate, and (iv) SEM image. (B) Gravimetric analysis of separate UV light printed disks, and violet or blue light printed supports upon soaking in ethyl acetate at room temperature with mild shaking. Symbols are averages (*n* = 3), and shaded regions represent ± 1 standard deviation from the mean. Inset: representative images of a disk print in a vial containing ethyl acetate. *F*_gel_ = gel fraction (wt %). (C) Stress–strain plots from uniaxial tensile testing of dogbones, as pictured. *E* = tensile modulus and σ_m_ = max stress with average values provided (*n* = 3). (D) Images of manual support removal and cleaning for a monolithic structure prepared using UV light (photosystem 1). (E) Average surface roughness characterized using profilometry on monolithic samples at different support removal stages (i–iv) and multimaterial samples with no (v) or one (vi) interfacial layer of dissolvable material. (F) Images of a resolution test print (UV light, photosystem 1) showing (i) several complex features and the need for dissolvable supports as indicated by (ii) a print without supports having collapsed bridges and (iii) rendering, (iv) pre-, and (v) postwashing a bridge with dissolvable supports.

Printing with resins comprising different amounts of chain transfer agent (EVS) unveiled its unique role (Figures S17–S18 and Table S13). In going from 2 to 1 to 0.5 mol % EVS dissolution in ethyl acetate became noticeably slower, while samples containing 0.25 and 0 mol % EVS visually swelled instead of dissolving over the course of 24 h. The lack of dissolution was attributed to light cross-linking, covalent and/or physical. Size exclusion chromatography revealed a number-average molecular weight of ∼11 kDa and dispersity of ∼2 relative to polystyrene standards for samples containing 2 mol % EVS (photosystem 1, violet light). Decreasing EVS led to an increase in molecular weight and dispersity, with the soluble components of samples containing 0.25 mol % having a molecular weight approaching 40 kDa, which is near the entanglement molecular weight for poly­(IBOA).^46^ In contrast, UV cured samples prepared with 2 mol % EVS left in ethyl acetate for ∼24 h did not dissolve, but swelled considerably (swelling ratio ≈ 53 ± 8% w/w Table S14). Drying these samples revealed a ∼9–13 wt % loss from the as printed samples, indicating a gel fraction (*F*_gel_) of ∼90 wt % (Figure 3B, legend). Thus, the incorporation of EVS facilitated rapid dissolution of supports printed with visible light, while maintaining high gel fractions for UV cured components.

Next, we characterized the individual mechanical properties of UV and violet or blue light cured structures (Figure 3C). Dogbones (ASTM D638 Type IV standards) were 3D printed and characterized under uniaxial tension (*n* ≥ 6). Both the soluble thermoplastic and insoluble thermoset materials were stiff plastics, providing comparable mechanical properties across the two photosystems. Specifically, the tensile modulus (*E*) for the violet or blue light cured support materials were 160 ± 40 MPa and 200 ± 50 MPa, respectively, while the corresponding stress at break (σ_m_) values were both 4.9 ± 0.6 MPa. Thus, the dissolvable material provided the requisite mechanical properties to act as support structures (e.g., lattices and bars) without the need for complete encapsulation of prints, greatly reducing the amount of waste and heat generation during printing known to lower max printing speeds and reduce resolution.^47^ For example, with the reference disk print cylindrical supports having diameters as small as 600 μm were possible, providing a fill density of ∼14 vol % (Figure S22 and Table S17). Moreover, angled supports (15°, 30°, and 45°) were effective (Figures S23–S24), and interfaces between dissolvable and nondissolvable domains were qualitatively strong as indicated by fracture of multimaterial dogbones under tension away from the interfaces (Figure S25). Compared to the thermoplastic material, the corresponding thermoset (UV light cured) was considerably more rigid and strong. Green body samples (only rinsed with isopropyl alcohol) prepared using photosystems 1 and 2 gave *E* values of 1.6 ± 0.1 GPa and 1.4 ± 0.1 GPa, respectively, along with corresponding σ_m_ values of 36 ± 5 MPa and 34 ± 2 MPa (Figure S26 and Table S18). Soaking in ethyl acetate for 20 min followed by drying resulted in a marginal reduction, with *E* values of 1.2 ± 0.1 GPa for both photosystems and σ_m_ values of 32 ± 0.8 MPa and 27 ± 2 MPa for photosystem 1 and 2, respectively (Figure 3C). The decrease in stiffness and stress at break may arise from defect formation, such as microcracks from swelling or microvoids during drying (Figure S28). However, all insoluble structures produced were easy-to-handle rigid plastics.

For comparison, contemporary postprocessing procedures for manual support removal were applied to a reference disk with supports printed using UV light exposure (Figure 3D). Using a Formlabs finishing kit the supports were sequentially removed by (i) cutting, (ii) sanding, (iii) grinding, and (iv) polishing. The entire procedure took ∼12 min for a single disk and was markedly challenging owing to its small size (∼14 mm diameter). This first-hand demonstration highlighted the difficulties associated with removing supports from simple flat objects and foreshadowed the impossible task of support removal from more complex 3D structures, such as a ball and socket joint. Next, we characterized the average surface roughness using a stylus profilometer and compared the results between manual support removal and our dissolvable supports (Figure 3E, Figures S30–S33, and Table S19). As expected, roughness decreased in going from cutting, (i) = 37 ± 9 μm, to sanding, (ii) = 19 ± 6 μm, to grinding, (iii) 6 ± 1 μm. In our hands, polishing provided a similar average roughness of 5 ± 1 μm relative to the penultimate finishing step. Of note, the average roughness from step (i), cutting, does not accurately reflect the support remnants, which had an average height of 190 ± 30 μm from the disk surface, clearly visible by scanning electron microscopy (SEM) (Figure S29). In comparison, samples with dissolvable supports directly connected to the insoluble disk resulted in an average surface roughness of 10 ± 1 μm, which was between that of sanding and grinding for the manual support removal. However, adding one 50 μm layer of dissolvable material between the supports and disk (Figure 3E inset) led to significantly smoother surfaces having an average roughness of 3 ± 1 μm. Additionally, angled supports with one dissolvable interface layer provided similarly smooth surfaces with average roughness <6 μm. Thus, the present dissolvable support strategy led to smooth surface finishes, where dissolvable interface layers further reduced roughness.

To assess the scope of printable features for the insoluble thermoset (photosystem 1) we employed a “Test your 3D printer” file (scaled down by 50%) that contained several challenging geometries, including small holes, slits, rounded components, overhangs, and bridges with variable gap distances (Figure 3F, i). Qualitatively all features printed with good fidelity, apart from long bridges that collapsed in the absence of supports (Figure 3F, ii). Introducing dissolvable supports (i.e., “piers”) remedied this issue, enabling free-standing bridges that were ∼7 mm long and only ∼250 μm (5 layers) thick between ∼500 μm wide insoluble piers (Figure 3F, iii–v). Additionally, we demonstrated that insoluble features <100 μm were achievable with the present process (Figures S39–S40) without adding opaquing agent (i.e., passive absorbers) commonly used in DLP 3D printing to improve resolution.^48^

To exemplify the present method, we 3D printed several proof-of-concept objects using photosystem 1 that benefited from, or required, support material (Figure 4 and Figures S45–S51). Prints included a detailed retainer (Figure 4A and Movie S2), a hook with a freestanding overhang (Figure 4B), and nonassembly structures, from interlocked chains (Figure 4C and Movie S3) to a ball in a box (Figures S48–S49 and Movie S4) and joints with different degrees of freedom (Figure 4D/E and Movies S5, S6, S7, and S8). For the retainer computed tomography (CT) scanning revealed excellent print fidelity, with an average distance of 140 μm between the digital input without supports and printed structure postdissolution of supports (Figure 4A (iv), Figure S52, and Movie S2). For the hook print, no orientation of the file on the build platform could avoid “floating” layers, necessitating supports (Figure 4B and Figure S46). For the chain mail example, two rows of five rings were interlocked, with each ring having a width of 1.5 mm. Despite having UV light exposed regions as close as 180 μm, all rings printed in-tact, without fusion between neighbors (Figure 4C (ii), Figure S47, and Movie S3). Nonassembly joints with internal floating components were chosen as the final examples given the need for supports that would be difficult (or impossible) to access for manual removal. Revolute (or rotary) and spherical (or socket) joints were selected as they represent two predominant building blocks for robotic actuators.^49^ Upon printing, washing, and drying the revolute joint displayed the intended one degree of freedom (rotation about a fixed axis), while the spherical joint displayed three degrees of freedom (roll, pitch, and yaw) (Figure 4D/E (ii and iii), Figures S50–S51, and Movies S5 and S7). CT scanning of each joint hanging upside down revealed a smooth gap between the individual components (Figure 4D/E (ii and iii), Figures S53–S54, and Movies S6 and S8). Furthermore, the revolute and spherical joints had average distances of 219 and 126 μm from the digital input (supports removed), showcasing the high feature fidelity achieved with the present process.

**Figure 4:**
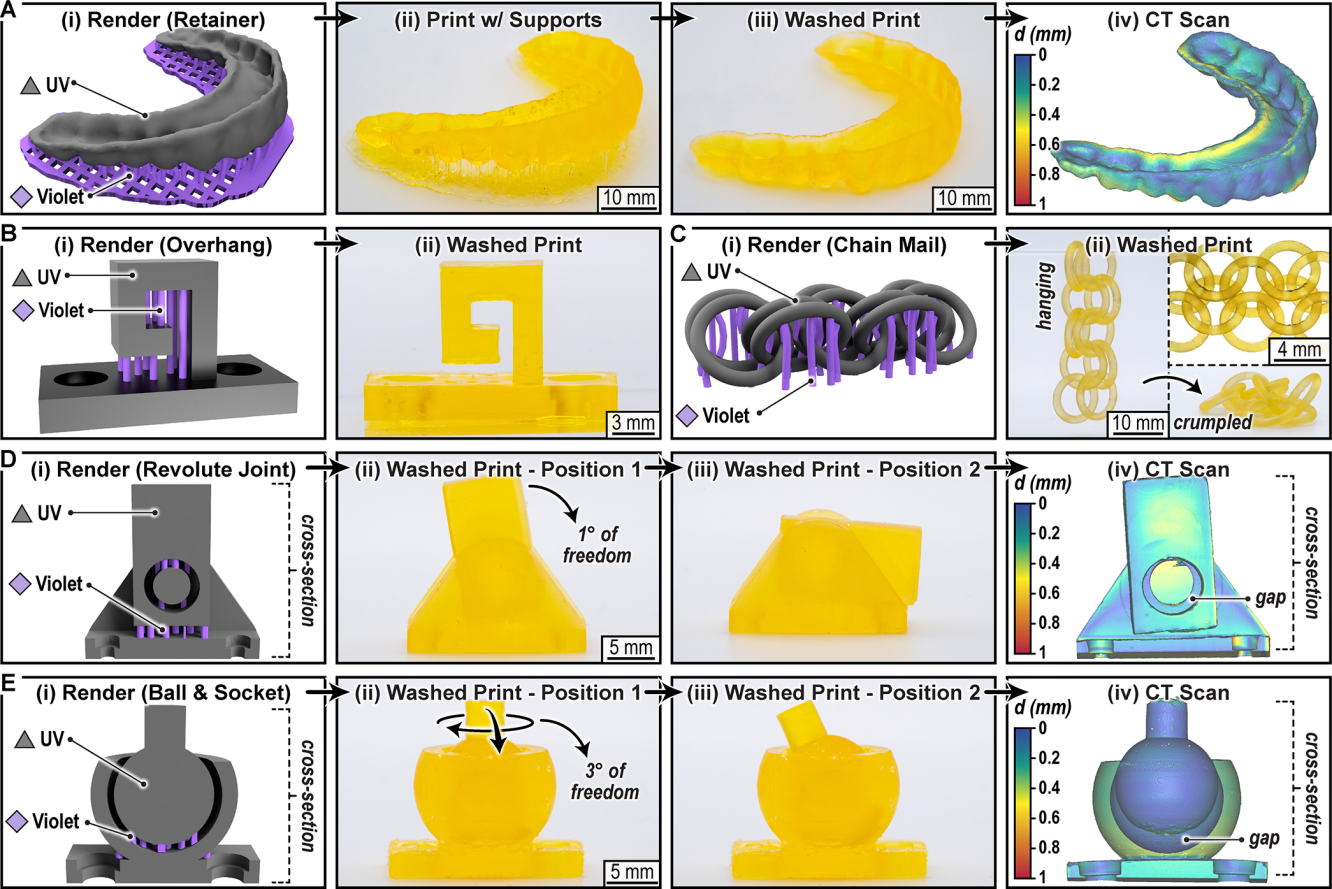
Structures enabled by multimaterial 3D printing with dissolvable supports. (A) Retainer showing (i) color-coded rendering, (ii) print with dissolvable supports, (iii) print after dissolving away supports, and (iv) computed tomography (CT) scan. (B) Hook with an overhang showing (i) digital rendering and (ii) image of a washed print. (C) Interlocked chains as a representative nonassembly structure showing (i) digital rendering and (ii) images of a washed print. (D) Revolute joint showing (i) digital rendering cross-section, (ii/iii) images of a washed print in two positions, and (iv) CT image cross-section. (E) Ball and socket joint, showing (i) digital rendering cross-section, (ii/iii) images of a washed print in two positions, and (iv) CT image cross-section. *d* = average distance from digital input.

## Concluding Remarks

Vat photopolymerization DLP 3D printing with wavelength-selective resins enabled the production of multimaterial objects composed of insoluble thermosets and readily soluble thermoplastics. Key to success was the selective violet or blue light activation of a radical polymerization to form a glassy, non-cross-linked polymer, and UV light activation of a cationic epoxy polymerization to form cross-links. Utility of a hybrid epoxy-acrylate monomer increased the maximum possible cross-linking density, photosensitizers and hydroxyl-containing comonomers accelerated acid generation and cationic cross-linking, and a chain transfer agent enhanced thermoplastic solubility and dissolution kinetics. As a result, high resolution multimaterial 3D printing was possible using 4–6 s of light exposure per 50 μm layer, with <100 μm insoluble features possible. Soaking the structures in ethyl acetate as a green solvent led to complete dissolution of support material within 10 min at room temperature, leaving behind a mechanically robust (*E* ≈ 1 GPa and σ_m_ ≈ 30 MPa) rigid plastic with a smooth surface finish (roughness <5 μm). This enabled the production of several structures containing geometries that required supports, including a hook with overhangs and mobile nonassembly joints. Looking ahead, alternative resin formulations could be examined to enhance mechanical properties, resolution, and printing speed while minimizing coloration. This includes using new reactive diluents, chain transfer agents, photoacid generators, and photosensitizers, while also incorporating opaquing agents. We anticipate that the present process is both scalable and automatable, which will facilitate the rapid manufacturing of next generation 3D structures, such as complex multisegmented actuators for robotics.

## Supplementary Material





















## Notes

Experimental (materials, instrumentation, and synthesis) and characterization details (PDF)
Representative example for support dissolution using a reference disk print containing 750 μm wide supports placed in ethyl acetate for five minutes to facilitate their dissolution (MP4)
Rotational view of retainer obtained from computed tomography scanning (MP4)
Movement of chainmail with gravity in real time (MP4)
Movement of washed ball in a box in real time (MP4)
Movement of washed revolute joint in real time (MP4)
Rotation and cross section view of revolute joint obtained from computed tomography scanning (MP4)
Movement of washed ball & socket joint in real time (MP4)
Rotation and cross section view of ball & socket joint obtained from computed tomography scanning (MP4)
Transparent Peer Review report available (PDF)

## Abbreviations

DLP: digital light processing
UV: ultraviolet
AM: additive manufacturing
IBOA: isobornyl acrylate
HEA: 2-hydroxethyl acrylate
ECA: 3,4-epoxycyclohexylmethyl acrylate
PAG: photoacid generator
MeOTX: 3,6-dimethoxy-9H-thioxanthen-9-one
DHS: diphenyl­[4-(phenylthiol)­phenyl]­sulfonium hexafluoroantimonate
ITX: isopropylthioxanthone
BAPO: phenylbis­(2,4,6-trimethylbenzoyl)­phosphine oxide
Ivocerin: bis­(4-methoxybenzoyl)­diethylgermanium
EVS: ethyl 2-(tosyloxy)­acrylate
ECC: 3,4-epoxycyclohexylmethyl 3,4-epoxycyclohexanecarboxylate
OXA: oxetane-alcohol
LED: light emitting diode
FTIR: Fourier transform infrared spectroscopy
ρ_max_: max conversion
*T*_g_: glass transition temperature
*F*_gel_: gel fraction
*E*: tensile modulus
σ_m_: max stress
SEM: scanning electron microscopy
CT: computed tomography
