# Supplementary material for: Multicolor Digital Light Processing 3D Printing Enables Dissolvable Supports for Freestanding and Non-Assembly Structures
Source: ACS Cent Sci. 2025 May 29;11(6):975–82. doi: 10.1021/acscentsci.5c00289 (PMC12203260; doi:10.1021/acscentsci.5c00289)
Supplement: Supplementary file 1 [file oc5c00289_si_001.pdf]

Supporting Information

Multicolor Digital Light Processing 3D Printing Enables Dissolvable Supports for Freestanding and Non-Assembly Structures

Keldy S. Mason,[a] Ji-Won Kim,[a] Elizabeth A. Recker,[b] Jenna M. Nymick,[a] Mingyu Shi,[a] Franz A. Stolpen,[a] Jaechul Ju,[a] Zachariah A. Page[a,b]*

[a]Department of Chemistry, The University of Texas at Austin, Austin, TX 78712, USA
[b]McKetta Department of Chemical Engineering, The University of Texas at Austin, Austin, TX, 78712, USA
***Corresponding authors:** *zpage@utexas.edu

Table of Contents

S1. EXPERIMENTAL DETAILS **S2**

S1.1. Materials *S2*

S1.2. Sample Preparation *S2*

S1.3. Synthesis *S5*

S1.4. Instrumentation *S6*

S2. CHARACTERIZATION **S11**

S2.1. Fourier Transform Infrared (FTIR) Spectroscopy *S11*

S2.2. Dynamic Mechanical Analysis (DMA) *S17*

S2.3. Thermogravimetric Analysis *S19*

S2.4. Dissolution Studies *S20*

S2.5. Molecular Weight Characterization *S21*

S2.6. Gel Fraction of 3D Printed Thermosets *S22*

S2.7. Viscosity Measurements *S24*

S2.8. Support Design *S25*

S2.9. Angled Supports *S26*

S2.10. Mechanical Properties *S28*

S2.11 SEM of Dried Samples *S31*

S2.12. Surface Roughness *S32*

S2.13. Supplemental Resolution Prints *S35*

S2.14. Supplemental Proof-of-Concept Prints *S40*

S2.15. Computed Tomography (CT) *S42*

S2.16. Cure Depth Experiments *S44*

S2.17. Real-Time UV-Vis Absorption Spectroscopy *S45*

S2.18. Nuclear Magnetic Resonance (NMR) Spectra *S47*

S3. MOVIE CAPTIONS **S50**

S4. REFERENCES **S51**

S1. EXPERIMENTAL DETAILS

S1.1. Materials

Chemicals. All reagents were used as received unless otherwise noted. Activated charcoal (powder, 100 particle size), copper oxide (Cu_2O) (97%), copper (98%), oxalyl chloride (98%), isopropylthioxanthone (mixture of 2- and 4- isomers) (ITX) (97%), and 2-hydroxyethyl acrylate (HEA) (96%) were purchased from Sigma-Aldrich. Diethylene glycol dimethyl ether (diglyme) (≥99.5%) was purchased from Chem-Impex. 3,4-Epoxycyclohexylmethyl acrylate (ECA) (96%) and diphenyl[4-(phenylthiol)phenyl]sulfonium hexafluoroantimonate (DHS) (98%) were purchased from Combi-blocks. *p*-Toluenesulfonyl chloride (>99.0%) was purchased from ACROS Organics. Ethyl acetate (EA) (certified ACS), dichloromethane (DCM) (certified ACS), hexanes (certified ACS), magnesium sulfate, hydrochloric acid, sodium bicarbonate (ACS grade), potassium carbonate (anhydrous), sodium chloride (certified ACS), and Celite were purchased from Fisher Scientific. Aluminum trichloride ($AlCl_3$) (99%, anhydrous), 1,4-diazabicyclo[2.2.2]octane (DABCO) (>98.0%), 2-ethoxyethanol (>99.0%), ethyl pyruvate (>97.0%), and 3-methoxybenzenethiol (>98.0%) were purchased from Tokyo Chemical Industry. $CDCl_3$ 99.8% was purchased from Cambridge Isotope Laboratories. Isobornyl acrylate (IBOA) (95%), 2-bromo-4-methoxybenzoic acid (98%), 3,4-epoxycyclohexylmethyl 3,4-epoxycyclohexanecarboxylate (ECC) (97%), and phenylbis(2,4,6-trimethylbenzoyl)phosphine oxide (BAPO) (99%) were purchased from Ambeed. 3-Ethyl-3-oxetanemethanol (OXA) (97%) was purchased from Synquest Laboratories. Bis(4-methoxybenzoyl)diethylgermanium (Ivocerin®) (96%) was purchased from Synthon. Anhydrous dichloromethane (DCM) and anhydrous dimethylformamide (DMF) were obtained from a Vac solvent purification system prior to their use and stored over molecular sieves. For all reactions, polymerizations, and measurements, no unexpected or unusually high safety hazards were encountered.

Non-chemicals. Analytical thin-layer chromatography was performed on Merck silica gel 60 F254 glass plates. Visualization was accomplished using a $KMnO_4$ stain or with ultra-violet (UV) light. Flash-column chromatography was performed using SUPELCO silica gel (technical grade, pore size 60 Å, 400 mesh particle size, 40-63 μm) that was purchased from Sigma-Aldrich.

S1.2. Sample Preparation

Resin Preparation. Resins were prepared in the dark by wrapping a 20 mL scintillation vial in aluminum foil. The vial was charged with solid photosystem components followed by the monomers (see **Tables S1** & **S2**). The vial was then sonicated until complete dissolution.

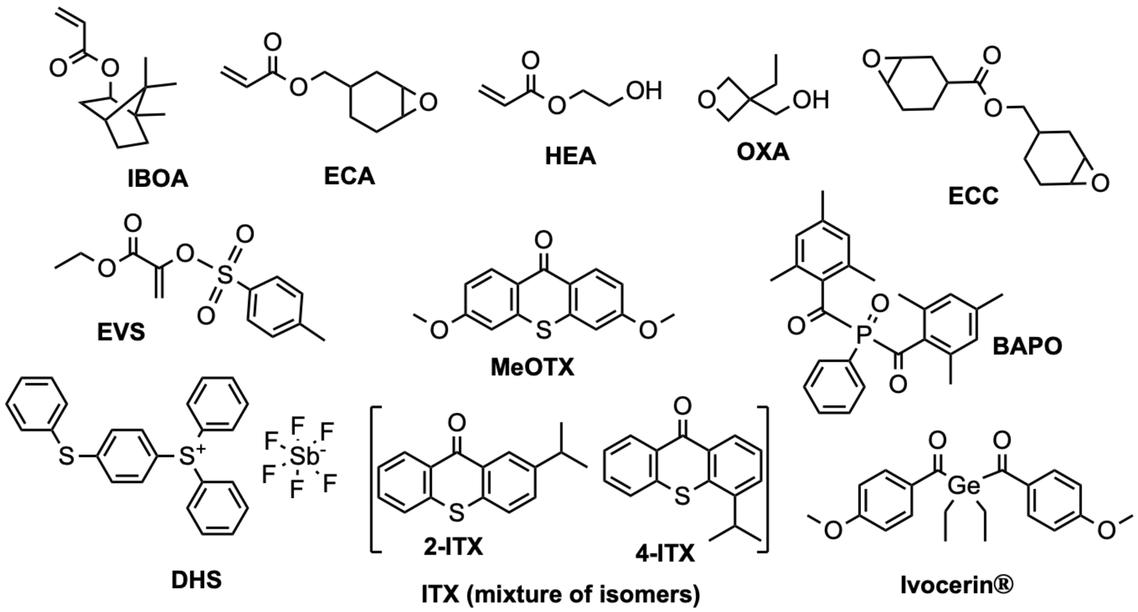

Figure S1. Chemical structures of components used in resins for dissolvable supports.

Table S1. Resin formulation for PS-1 resins.

Component	Mass (mg)	Mol%/ Wt%
3,4-Epoxycyclohexylmethyl Acrylate (ECA)	1368	25.0/ 24.3
Isobornyl acrylate (IBOA)	3240	51.8/ 57.6
2-hydroxyethyl acrylate (HEA)	696.8	20.0/ 12.4
diphenyl[4-(phenylthiol)phenyl]sulfonium hexafluoroantimonate (DHS)	92.0	0.5/ 1.6
Ethyl 2-(tosyloxy)acrylate (EVS)	163.0	2.0/ 2.9
Phenylbis(2,4,6-trimethylbenzoyl)phosphine oxide (BAPO)	26.0	0.2/ 0.5
3,6-dimethoxythioxanthone (MeOTX)	41.0	0.5/ 0.7

Table S2. Resin formulation for PS-2 resins.

Component	Mass (mg)	Mol%/ Wt%
3,4-Epoxycyclohexylmethyl Acrylate (ECA)	1368	25.0/ 24.3
Isobornyl acrylate (IBOA)	3240	51.8/ 57.6
2-hydroxyethyl acrylate (HEA)	696.8	20.0/ 12.4
diphenyl[4-(phenylthiol)phenyl]sulfonium hexafluoroantimonate (DHS)	92.0	0.5/ 1.6
Ethyl 2-(tosyloxy)acrylate (EVS)	162.4	2.0/ 2.9
(diethylgermanediyl)bis((4-methoxyphenyl)methanone) (Ivocerin)	24.1	0.2/ 0.4
isopropylthioxanthone (mixture of 2- and 4- isomers) (ITX)	38.4	0.5/ 0.7

Table S3. Resin formulation for PS-3 resins.

Component	Mass (mg)	Mol%/ Wt%
3,4-Epoxycyclohexylmethyl Acrylate (ECA)	1368	25.0/ 24.3
Isobornyl acrylate (IBOA)	3240	51.8/ 57.6
2-hydroxyethyl acrylate (HEA)	696.8	20.0/ 12.4
diphenyl[4-(phenylthiol)phenyl]sulfonium hexafluoroantimonate (DHS)	92.0	0.5/ 1.6
Ethyl 2-(tosyloxy)acrylate (EVS)	163.0	2.0/ 2.9
(diethylgermanediyl)bis((4-methoxyphenyl)methanone) (Ivocerin)	26.0	0.2/ 0.4
3,6-dimethoxythioxanthone (MeOTX)	41.0	0.5/ 0.7

Table S4. Resin formulation for PS-1 proxy resins to track epoxide conversion.

Component	Mass (mg)	Mol%/ Wt%
3,4-Epoxycyclohexylmethyl 3,4-epoxycyclohexanecarboxylate (ECC)	959.2	38.0/55.8
3-Ethyl-3-oxetanemethanol (OXA)	706.1	60.8/41.1
Diphenyl[4-(phenylthiol)phenyl]sulfonium hexafluoroantimonate (DHS)	30.4	0.5/1.8
Phenylbis(2,4,6-trimethylbenzoyl)phosphine oxide (BAPO)	8.4	0.2/0.5
3,6-dimethoxythioxanthone (MeOTX)	13.6	0.5/0.8

Table S5. Resin formulation for PS-2 proxy resins to track epoxide conversion.

Component	Mass (mg)	Mol%/ Wt%
3,4-Epoxycyclohexylmethyl 3,4-epoxycyclohexanecarboxylate (ECC)	959.5	38.0/55.9
3-Ethyl-3-oxetanemethanol (OXA)	705.9	60.8/41.1
Diphenyl[4-(phenylthiol)phenyl]sulfonium hexafluoroantimonate (DHS)	30.4	0.5/1.8
(diethylgermanediyl)bis((4-methoxyphenyl)methanone) (Ivocerin)	8.0	0.2/0.5
isopropylthioxanthone (mixture of 2- and 4- isomers) (ITX)	12.7	0.5/0.7

Table S6. Resin formulation for PS-3 proxy resins to track epoxide conversion.

Component	Mass (mg)	Mol%/ Wt%
3,4-Epoxycyclohexylmethyl 3,4-epoxycyclohexanecarboxylate (ECC)	959.2	38.0/55.8
3-Ethyl-3-oxetanemethanol (OXA)	706.1	60.8/41.1
Diphenyl[4-(phenylthiol)phenyl]sulfonium hexafluoroantimonate (DHS)	30.4	0.5/1.8
(diethylgermanediyl)bis((4-methoxyphenyl)methanone) (Ivocerin)	8.0	0.2/0.5
3,6-dimethoxythioxanthone (MeOTX)	13.6	0.5/0.8

S1.3. Synthesis

The two-step synthesis of photosensitizer MeOTX followed a previous protocol developed by our group.[S1]

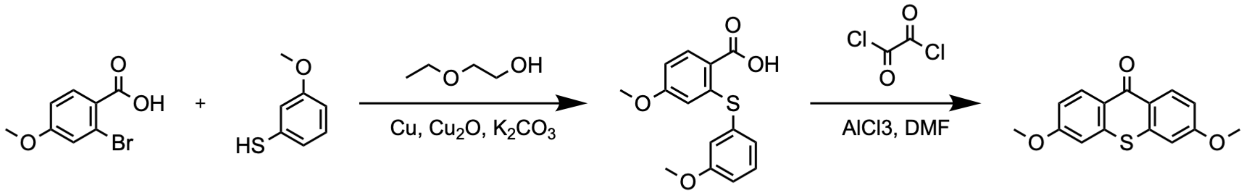

Scheme S1. Two-step synthetic scheme for 3,6-dimethoxy-9H-thioxanthen-9-one (MeOTX) synthesis.

4-methoxy-2-((3-methoxyphenyl)thio)benzoic acid. White powder. ^{1}H NMR (400 MHz, $CDCl_3$) δ 8.11 (d, J = 8.7 Hz, 1H), 7.41 – 7.34 (m, 1H), 7.22 – 7.13 (m, 2H), 6.99 (ddd, J = 8.4, 2.6, 1.0 Hz, 1H), 6.66 (dd, J = 8.8, 2.4 Hz, 1H), 6.31 (d, J = 2.5 Hz, 1H), 3.82 (s, 3H), 3.64 (s, 3H). ^{13}C NMR (101 MHz, $CDCl_3$) δ 171.43, 163.37, 160.48, 147.38, 134.42, 132.84, 130.64, 128.33, 120.72, 117.56, 115.87, 112.42, 109.79, 55.44, 55.22. HRMS (ESI): exact mass calculated for C15H14O4S [M+Na]+ 313.0505, found 313.0507.

3,6-dimethoxythioxanthone (MeOTX). Off-white solid. ^{1}H NMR (400 MHz, $CDCl_3$) δ 8.51 (d, J = 8.9 Hz, 2H), 6.99 (dd, J = 9.0, 2.6 Hz, 2H), 6.90 (d, J = 2.5 Hz, 2H), 3.89 (s, 6H). ^{13}C NMR (101 MHz, $CDCl_3$) δ 178.33, 162.26, 139.14, 131.80, 123.13, 114.82, 108.06, 55.67. HRMS (ESI): exact mass calculated for C15H12O3S [M+Na]+ 295.0399, found 295.0403.

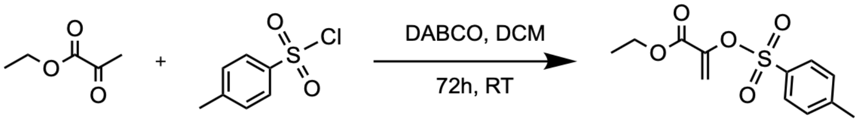

Scheme S2. Synthetic scheme for ethyl 2-(tosyloxy)acrylate (EVS) synthesis.

Ethyl 2-(tosyloxy)acrylate (EVS). This procedure was adapted from Liska and coworkers.[S2] To a flame-dried 1 L three-neck round-bottom flask equipped with a magnetic stir bar and under an inert atmosphere (argon) was added DABCO (19.71 g, 1.7 eq., 175.7 mmol) and anhydrous CH_2Cl_2 (90 mL). To this, a solution of freshly distilled ethyl pyruvate (12.00 g, 1.00 eq, 103.30 mmol) and 4-toluenesulfonyl chloride (29.55 g, 1.50 eq, 155.0 mmol) in anhydrous CH_2Cl_2 (310 mL) was added dropwise via a 500 mL oven-dried addition funnel. The reaction mixture was stirred at room temperature under a nitrogen atmosphere for 72h. The reaction progress was monitored using TLC (hexanes:ethyl acetate, 4:1). Upon completion, the reaction mixture was filtered through silica gel, washed with CH_2Cl_2 (100 mL). The organic phase was washed sequentially with 1.5 N HCl (2 × 100 mL) and deionized water (100 mL). The combined aqueous phases were extracted with CH_2Cl_2 (50 mL), and the combined organic phases were washed with brine (70 mL), dried over Na_2SO_4, filtered, and concentrated in vacuo. Further purification by column chromatography (hexanes:ethyl acetate, 4:1 as the eluent) yielded the product as a colorless crystalline solid (18.5 g, 66% yield). ^{1}H NMR (500 MHz, $CDCl_3$) δ 7.87 – 7.81 (m, 2H), 7.36 (d, J = 8.1 Hz, 2H), 6.14 (d, J = 2.2 Hz, 1H), 5.63 (d, J = 2.2 Hz, 1H), 4.15 (q, J = 7.1 Hz, 2H), 2.46 (s, 3H), 1.22 (t, J = 7.1 Hz, 3H). ^{13}C NMR (126 MHz, $CDCl_3$) δ 160.95, 145.64, 143.32, 132.61, 129.76, 128.58, 116.97, 62.03, 21.70, 13.93. HRMS (ESI): exact mass calculated for C12H14O5S [M+Na]+ 293.0454, found 293.0450.

S1.4. Instrumentation

S1.4.1. Nuclear Magnetic Resonance (NMR) Spectroscopy. NMR spectra were recorded at room temperature on an Agilent MR spectrometer (400 MHz for ^{1}H NMR and 100 MHz for ^{13}C NMR) or a Bruker AVIII 500 (500 MHz for ^{1}H NMR) utilizing $CDCl_3$ or CD_2Cl_2 as the solvent. ^{1}H NMR were carried out coupled and referenced to the $CDCl_3$ chemical shift or CD2Cl_2 chemical shift at 7.26 ppm or 5.30 ppm, respectively. ^{13}C NMR was carried out decoupled and referenced to the $CDCl_3$ chemical shift at 77.16 ppm. Data reported as multiplicity (s = singlet, br s = broad singlet, d = doublet, t = triplet, q = quartet, m = multiplet).

S1.4.2. Real-Time Fourier Transform Infrared Spectroscopy (RT-FTIR). Light-induced polymerizations were monitored in real-time using FTIR spectroscopy (INVENIO-R, Bruker), controlled via OPUS spectroscopy software. A horizontal transmission accessory (A043-N/Q, Bruker) was used under ambient condition to provide an analogous environment to 3D printing. For in-situ light irradiation, 365 nm, 405 nm, or 460 nm LEDs (Type H, Mightex) were connected to the transmission accessory through a liquid light guide (LLG5-4H, Thorlabs) with a collimator (LGC-019-022-05-V, Mightex). To control light intensities, an LED driver (M/N SLC-MA02-U, Mightex) was used, and the intensities on the samples were measured by a thermal photodiode power sensor (S401C, Thorlabs) which was connected to a photometer (PM100D, Thorlabs). The samples were prepared in between either salt plates (NaCl plates, 1 inch × 1 inch × 4 mm, International Crystal Laboratories) or glass slides (1 mm × 25 mm × 75 mm, Fisher Scientific) with spacers (50 µm or 100 µm as noted, McMaster-Carr) to provide uniform thicknesses during photo-polymerizations. Spectra were taken over 120 s, and specific peaks (3100 cm^{-1} for C=C–H from acrylate, and 750 cm^{-1} for epoxide groups)[S3] were integrated over time to provide conversion-time curves. Both LEDs were turned on after 10 s of IR measurement to ensure resins were non-reactive in dark (i.e., provided temporal control).

S1.4.3. UV-Visible Spectroscopy. Absorptivity in the ultraviolet and visible spectral regions for each of the photosystem components was characterized using UV-vis absorption spectroscopy (QE PRO-ABS, Ocean Insight). A deuterium-tungsten halogen light source (DH-2000-BAL, Ocean Insight) was utilized as the probe light, with connection to the spectrophotometer via optical fibers (QP600-025-SR, Ocean Insight) by passing through a horizontal transmission accessory (Stage RTL-T, Ocean Insight). To measure molar absorptivity of each photosystem component, compounds were dissolved into the main resin monomers at the concentration equivalent to the full resin system and measured between glass slides with 50 µm thick spacers (**Figure S2**). For real-time measurements, light from an LED was controlled using strobe mode (M/N SLC-MA02-U, Mightex) to preclude LED scatter, and an automated LabView code was created to capture the absorption spectra every 100 ms. Finally, LED emission profiles from both the type-H LEDs used for UV-vis and FTIR absorption spectroscopy, as well as printer LED profiles were, collected using the same UV-vis spectrophotometer.

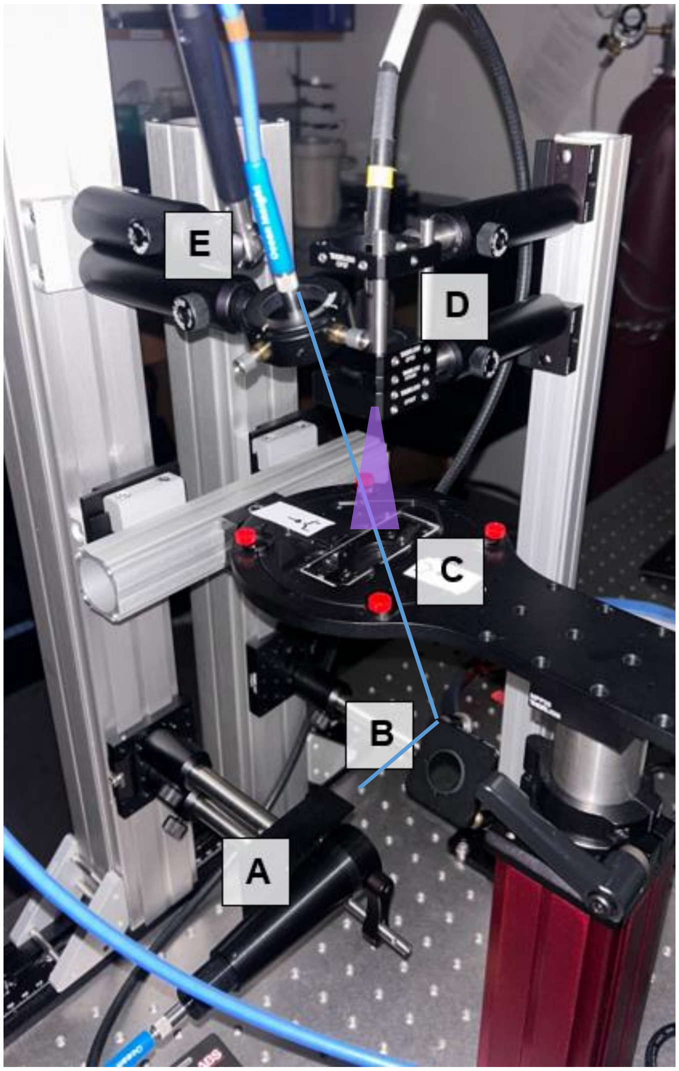

Figure S2. Real-time UV-vis absorption spectroscopy setup. **A** corresponds to UV-vis beam emission side, **B** is a mirror that reflects the beam through the sample and into the detector, and **C** is the staging area where the sample is placed. Directly underneath the stage is a focusing lens and aperture. **D** corresponds to the light guide and collimator that is connected to a light source; **E** corresponds to the UV-vis beam detector. The blue line represents the path of the UV-vis source beam, while the purple shading represents the LED irradiation.

S1.4.4. Two Channel DLP 3D Printer. 3D printing was performed using a custom-made, digital light processing (DLP) 3D printer (MONO3VZ4, Monoprinter, MA, USA) (**Figure S3**). The printer contained two LED projectors with wavelengths centered at 365 and 405 nm (for configuration 1), or 365 and 460 nm (for configuration 2). A dichroic mirror (T387lp, 35 mm × 50 mm × 1 mm, Chroma) was used to combine the two LED projections. The projector resolution was 1600 × 2560 pixels, with each pixel at the image plane being 15.1 μm × 15.1 μm (LRS-20 lenses) or 35 μm × 35 μm (LRS-50 lenses). Unless noted otherwise, digital files for each LED projection were generated by designing the 3D models using computer-aided design (CAD) software (Blender, SolidWorks) and exported into an STL format. Scans of one of the authors teeth were obtained by Forest Family Dentistry and used for generating .STL files of the retainer. Supports were generated with Netfabb support generation with the bars on area support script and

a critical angle of 40°, anchor distance of 1.65-2.00 mm, right angle at part, circular pattern, and varied support widths from 600-1000 μm. Supports were exported as a separate STL file to facilitate wavelength programming. Each STL file was imported into MonoWare software, aligned, supports were added, and the object was sliced into a 2D MNF file with a slice thickness of 50 μm. The exposure times per slice for each LED could be controlled independently, and light intensities were controlled with digital-to-analog converter (DAC) values of each LED. Unless noted otherwise, prints were conducted with 50 mW/cm^2 and an exposure time of 4 seconds per 50 μm layer. The MNF files were then simultaneously projected onto the bottom of a resin vat with maximum image plane dimensions of 25 mm × 44 mm (smaller vat) or 56 mm × 89.6 mm (larger vat). A transparent fluorinated ethylene propylene (FEP) polymer film (Teflon FEP film, 127 μm thick, DuPont) was used as the base of the resin vat to provide a non-stick bottom surface.

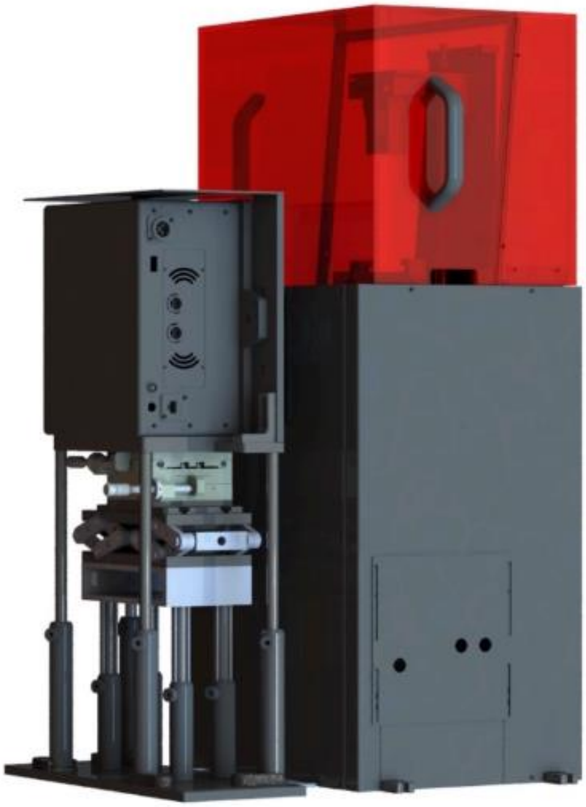

Figure S3. A custom digital light processing (DLP) 3D printer from Monoprinter. The printer contains two LED projectors from Visitech (LRS-WQ SL LC). The projector resolution with two LRS-20 lenses is 1600 × 2560 pixels, with each pixel being 15.1 μm × 15.1 μm at the image plane. The total build area for this lens configuration is 24.16 mm × 38.65 mm. The projector resolution with two LRS-50 lenses is 1600 × 2560 pixels, with each pixel being 35 μm × 35 μm at the image plane. The total build area for this lens configuration is 56 mm × 89.6 mm.

S1.4.5. Thermogravimetric Analysis (TGA). TGA was performed on a TA Instruments TGA Q500 with a heat ramp of 5.00 °C/min to 30 °C, followed by a 15 minute isothermal hold, and then a heat ramp of 5.00 °C/min to 800 °C.

S1.4.6. Gel Permeation Chromatography (GPC). Gel Permeation Chromatography was performed on a Tosoh EcoSEC HLC-8320 GPC System equipped with a series of TSKgel SuperHM-H, -M,

and -N columns. The instrument is equipped with a refractive index (RI) and ultraviolet (UV) absorbance detectors (UV-8320). The instrument was run with HPLC-grade tetrahydrofuran as the eluent, at a flowrate of 0.50 mL/min, temperature of 40 °C, and injection volume of 50-100 μL. Prior to injection, all samples were fully dissolved in the mobile phase at a concentration of ~1-5 mg/mL and passed through a 0.45 μm syringe filter. Molecular weight and molecular weight distributions for the samples were estimated using a calibration curve generated from a set of polystyrene standards (PstQuick® Kit-H) purchased from Tosoh Bioscience.

S1.4.7. Tensile Testing. Uniaxial Tensile testing was carried out using a Shimadzu Autograph AGS- X universal testing machine equipped with a 1 kN load cell. The uniaxial testing of bulk films was performed on 30% scaled ASTM D638 Type IV printed dogbone samples (1.8 mm width, 19.5 mm gauge length, 1-1.5 mm thickness). Samples were loaded with minimal slack and subjected to a 0.05 N pre-force at a strain rate of 100%/minute, followed by data collection. Shimadzu software was used to obtain Young's modulus at <0.1% strain, maximum stress, and maximum strain based on experimentally measured stress-strain curves. All samples were tested under ambient conditions.

S1.4.8. Rheology. Rheology was performed with a Discovery HR2 TA Instruments to determine viscosity of a diglyme solution containing dissolved 3D printed support material that were prepared using 405 or 460 nm light projections. Specifically, disks were printed (2-mm thick and 7-mm in diameter) and then dissolved in diglyme for ~12 hours. The viscosity was obtained using a flow sweep mode, varying shear rate from 10 s^{-1} to 500 s^{-1} at 25 °C. For the flow sweep test, a Peltier plate was used as the base along with a 20 mm steel plate top plate.

S1.4.9. Contact Profilometry. Surface roughness was characterized using a Dektak 6M Stylus Profilometer, equipped with a high-aspect-ratio stylus featuring a 25 µm tip radius. The instrument was operated in the 2620 kÅ range, providing a vertical resolution of 40 Å. Measurements were conducted over a 2000 µm scan length with a duration of 30 seconds, capturing 9000 data points at a scanning velocity of 0.222 µm per sample. To ensure statistical reliability, at least six measurements were performed for each sample, with both averages and standard deviations calculated. All tests were conducted under ambient conditions. Average height of cut support remnants was determined with six measurements using a Mitutoyo 543-392B Absolute Digital Indicator with small tipped probe accessory (~0.25 mm).

S1.4.10. Scanning Electron Microscopy (SEM). SEM studies were carried out on a FEI Quanta 650 SEM/ ESEM instrument at 5-10 kEv. SEM was used to examine the cross-section and surface characteristics of the 3D printed objects. For improved imaging, the samples were sputtered with platinum using an Electron Microscopy Science (EMS) sputter coater. Sputtering was conducted for 1.5 min at 40 mA with a target layer thickness of 5 nm.

S1.4.11. High Resolution X-Ray Computed Tomography (CT). All specimens were scanned by Dr. Jessica Maisano on a Zeiss Xradia 620 Versa using the flat panel detector, with X-ray source settings at 70kV and 8.5W, and detector position (190.5 mm) and source position (-47.3 mm) resulting in 14.88 µm resolution. For joint samples, silicone grease was added to the gap for imaging to maintain a fixed position during scanning. Scanning protocols and data reconstruction details are provided within the caption for each figure in the supplemental images below.

S1.4.12. Dynamic Mechanical Analysis (DMA). Dynamic mechanical analysis (Q800 DMA, TA Instruments) was used to characterize glass transition temperature (T_g) for all samples and rubbery storage modulus (E') for crosslinked samples (i.e., thermosets produced using UV light).

Rectangular bars (30 mm × 5 mm × 2 mm) were 3D printed and each dimension (width, thickness) was measured prior to testing. Samples were tested under at a frequency of 1 Hz and an amplitude of 0.1% strain, with a heating rate of 3 °C/min, and a temperature range of 30-175 °C.

S1.4.13. Optical/Digital Microscopy. An optical microscope (Eclipse LV100ND, Nikon) was utilized to image samples. A halogen lamp (LV-LH50PC-CH, Nikon) was used as a backlight at the bottom. Additionally, a digital microscope (VHX-7000, Keyence and Edge WF4915ZT, Dino-Lite) was used to take optical images of 3D printed structures.

S2. CHARACTERIZATION

S2.1. Fourier Transform Infrared (FTIR) Spectroscopy

Light-induced polymerizations were monitored in real-time using FTIR spectroscopy tracking either acrylate or epoxy conversion by integrating specific peaks during controlled irradiation from LEDs (**Figure S4**). Here we show conversion data for a set of photosystems, including both the full resins (acrylate + epoxy mixtures) and epoxy proxy systems (epoxy only) adding one component at a time to assess their individual roles. Data was collected for each system at three LED wavelengths (365nm, 405nm, and 460nm).

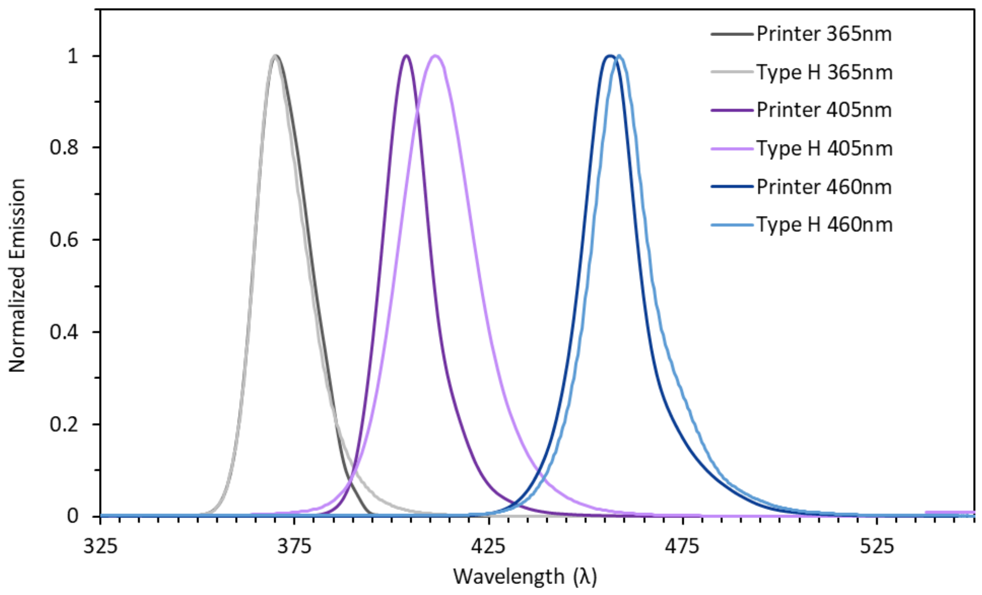

Figure S4. Overlay of each printer and type H LED used in experiments for this work. All spectra were collected at an intensity set to 50 mW/cm^2.

Table S7. Summary of light source peak wavelengths and full width at half max (FWHM).

Light Source	Peak Wavelength (nm)	FWHM (nm)
Printer 365	370	16
Printer 405	404	15
Printer 460	456	17
Type-H 365	370	15
Type-H 405	411	22
Type-H 460	459	17

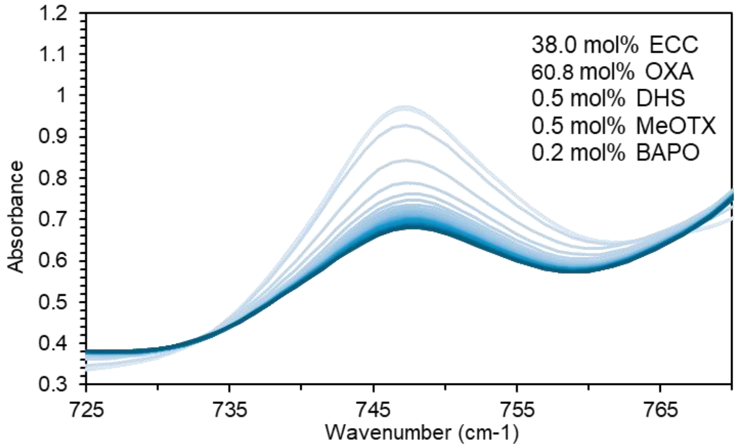

Figure S5. Representative real-time FTIR spectra used to monitor the C–O–C asymmetric stretch at 750 cm^{-1}. Conversion of epoxide was characterized using Bruker's OPUS software with a Type-B integration method.

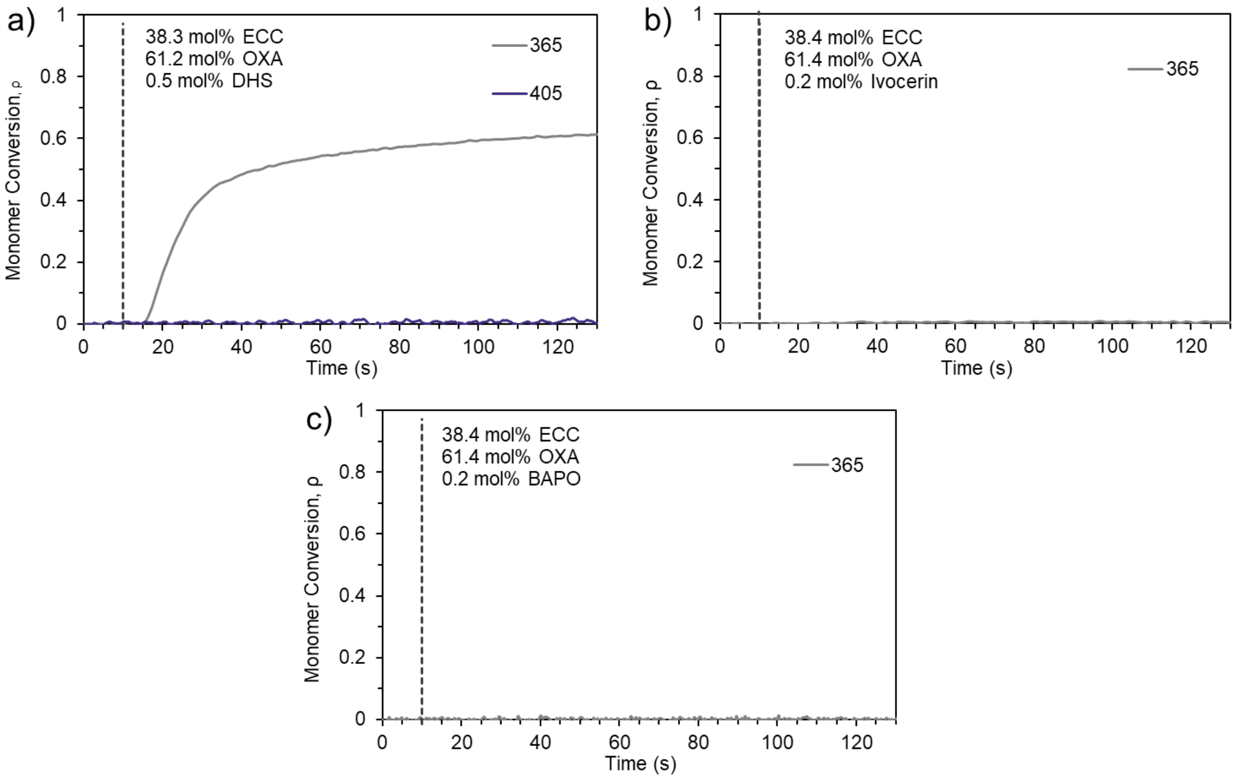

Figure S6. Conversion of epoxide groups monitored using FTIR in a transmission configuration upon exposure to an LED (365 nm and/or 405 nm) at an intensity of 50 mW/cm^2. Samples were 50 µm thick between salt plates (NaCl), containing pure 3,4-epoxycyclohexylmethyl 3,4-epoxycyclohexanecarboxylate (ECC) and 3-ethyl-3-oxetanemethanol (OXA) with (a) diphenyl[4-(phenylthio)phenyl]sulfonium hexafluoroantimonate (DHS), (b) Ivocerin, and (c) BAPO.

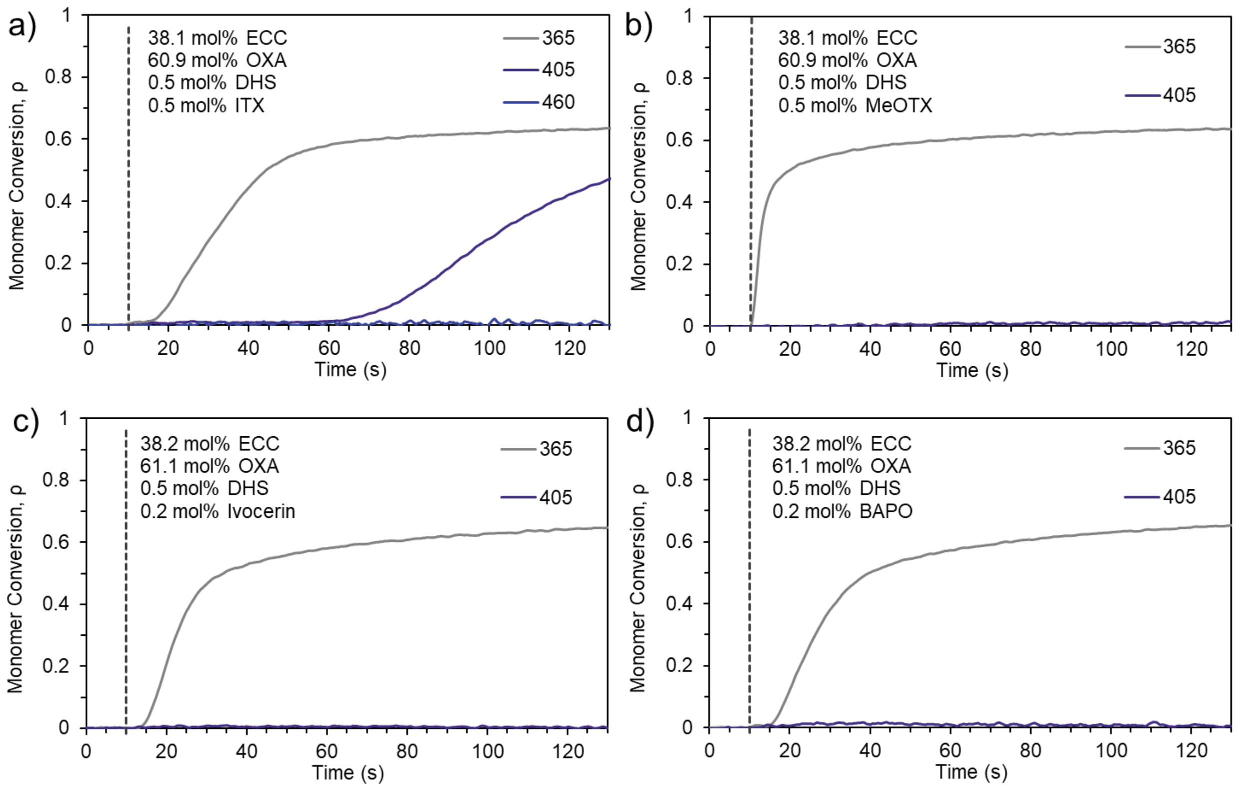

Figure S7. Conversion of epoxide groups monitored using FTIR in a transmission configuration upon exposure to an LED (365 nm, 405, and/or 460 nm) at an intensity of 50 mW/cm^2. Samples were 50 µm thick between salt plates (NaCl), containing ECC and OXA with (a) DHS and 2-isopropylthioxanthone (ITX), (b) DHS and 3,6-dimethoxy-9H-thioxanthen-9-one (MeOTX), (c) DHS and Ivocerin, and (d) DHS and BAPO.

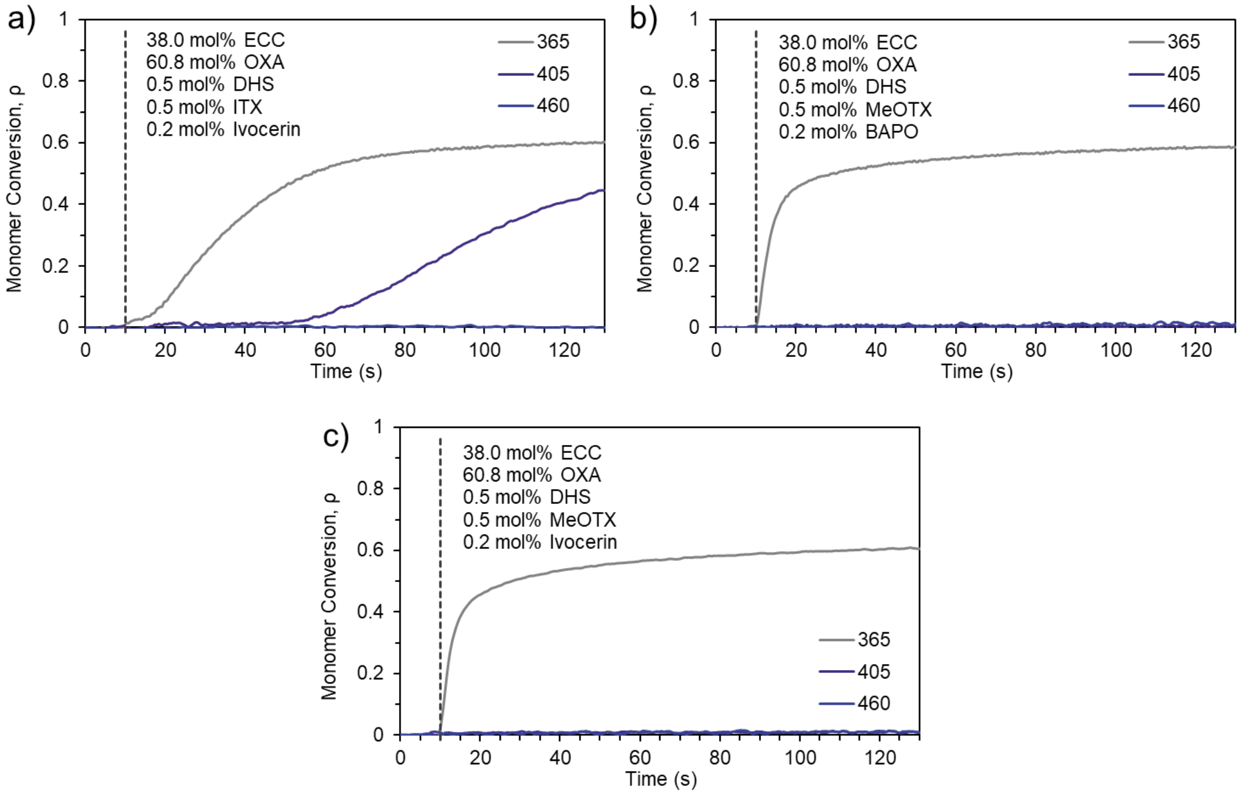

Figure S8. Conversion of epoxide groups monitored using FTIR in a transmission configuration upon exposure to an LED (365 nm, 405, and/or 460 nm) at an intensity of 50 mW/cm^2. Samples were 50 µm thick between salt plates (NaCl), containing ECC, OXA and DHS with (a) ITX and Ivocerin, (b)MeOTX and BAPO, and (c) MeOTX and Ivocerin.

Table S8. Summary of real time FTIR results for epoxy proxy resin system measured in a transmission configuration with salt plates (NaCl), monitoring at 750 cm^{-1} (Figures S5-S8).

Resin Components	Average Max Conversion (frac ± σ)			Average Initial Rate (mM/ s ± σ)		
**(ECC + OXA + …)**	**365**	**405**	**460**	**365**	**405**	**460**
BAPO	<0.01	–	–	<1	–	–
Ivocerin	<0.01	–	–	<1	–	–
DHS	0.62 ± 0.01	<0.01	–	179 ± 7	<1	–
DHS + BAPO	0.65 ± 0.02	<0.01	–	125 ± 4	<1	–
DHS + Ivocerin	0.65 ± 0.03	<0.01	–	201 ± 6	<1	–
DHS + ITX	0.63 ± 0.01	0.48± 0.04	<0.01	100 ± 10	45 ± 1	<1
DHS + MeOTX	0.64 ± 0.02	<0.01	–	620 ± 50	<1	–
(PS1) DHS + MeOTX + BAPO	0.59 ± 0.01	<0.01	<0.01	450 ± 30	<1	<1
(PS2) DHS + ITX + Ivocerin	0.60 ± 0.03	0.45 ± 0.02	<0.01	80 ± 3	50 ± <1	<1
(PS3) DHS + MeOTX + Ivocerin	0.61 ± 0.01	<0.01	<0.01	500 ± 20	<1	<1

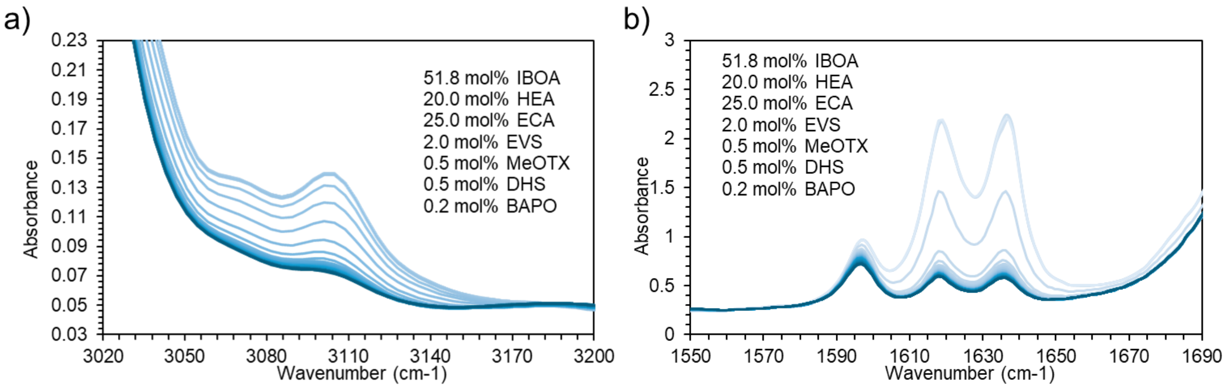

Figure S9. Representative real-time FTIR spectra used to monitor a) the C=C–H asymmetric stretch at 3100 cm^{-1} and b) the C=C stretch at 1620 cm^{-1}. Conversion of acrylate was characterized using Bruker's OPUS software with a Type-F integration method for the 3100 cm^{-1} signal, and a Type-B integration method for the 1620 cm^{-1} signal.

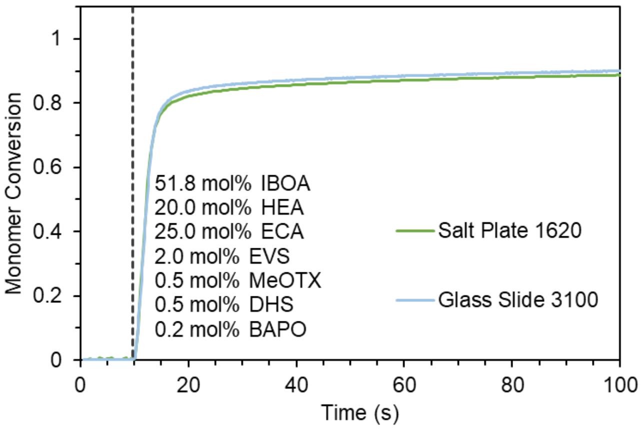

Figure S10. Conversion of acrylate groups monitored using the full photosystem 1 resin via FTIR in a transmission configuration upon exposure to an LED (365 nm, 50 mW/cm^2). Samples were 50 μm thick between NaCl salt plates (green trace) or between glass slides (blue trace). For data collected with salt plates, the C=C stretch was monitored at 1620 cm^{-1} and for data collected with glass plates, the C=C–H asymmetric stretch was monitored at 3100 cm^{-1}. Given good agreement between these traces, acrylate conversion was measured for all samples on the disposable glass slides.

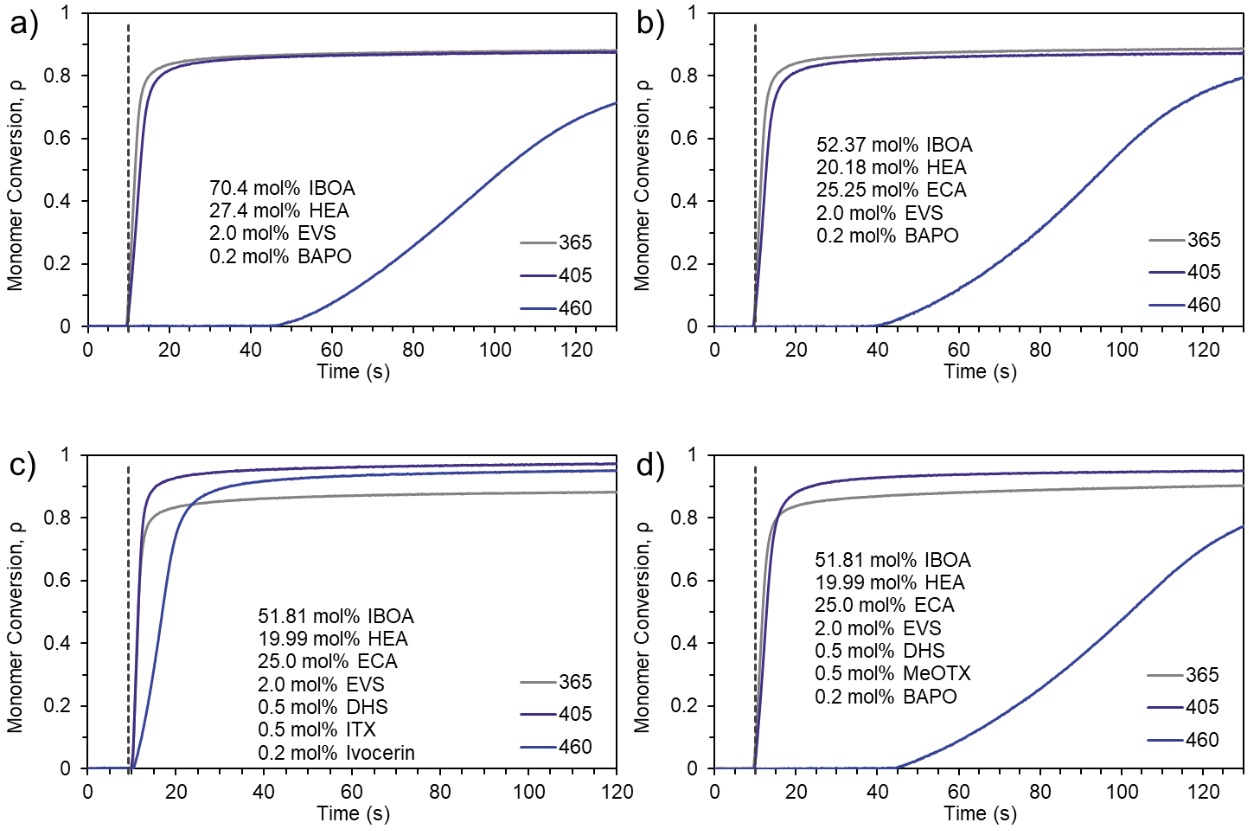

Figure S11. Conversion of acrylate groups monitored using FTIR in a transmission configuration upon exposure to an LED (365 nm, 405 nm, or 460 nm) at an intensity of 50 mW/cm^2. Samples were 50 μm thick between glass plates, containing IBOA, HEA, and EVS with (a) BAPO, (b) ECA and BAPO, (c) ECA, ITX, DHS, and Ivocerin, and (d) ECA, MeOTX, DHS and BAPO.

Table S9. Summary of real time FTIR results for multimaterial (hybrid) resin system measured in a transmission configuration with glassplates, monitoring at 3100 cm^{-1}.

Resin Components	Average Max Conversion (frac ± σ)			Average Initial Rate (mM/s ± σ)		
**(IBOA + HEA + EVS + …)**	**365**	**405**	**460**	**365**	**405**	**460**
BAPO	0.88 ± 0.01	0.88 ± 0.03	0.73 ± 0.04	1373 ± 9	880 ± 10	50 ± 4
ECA + BAPO	0.88 ± 0.03	0.87 ± 0.00	0.80 ± 0.00	1480 ± 60	945 ± 1	41 ± 3
(PS1) ECA + DHS + MeOTX + BAPO	0.90 ± 0.03	0.95 ± 0.01	0.79 ± 0.01	1360 ± 20	952 ± <1	36 ± 0
(PS2) ECA + DHS + ITX + Ivocerin	0.88 ± 0.01	0.97 ± 0.03	0.95 ± 0.03	2020 ± 90	2140 ± 80	391 ± 6
(PS3) ECA + DHS + MeOTX + Ivocerin	0.92 ± 0.03	0.96 ± 0.02	0.94 ± 0.02	1420 ± 40	2100 ± 100	540 ± 30

S2.2. Dynamic Mechanical Analysis (DMA)

Dynamic Mechanical Analysis. DMA tensile oscillation temperature sweeps were carried out for samples prepared with either photosystem 1, 2 , or 3 using the 365 nm/405 nm 3D printing setup. Crosslinking density can be estimated by the affine network theory.

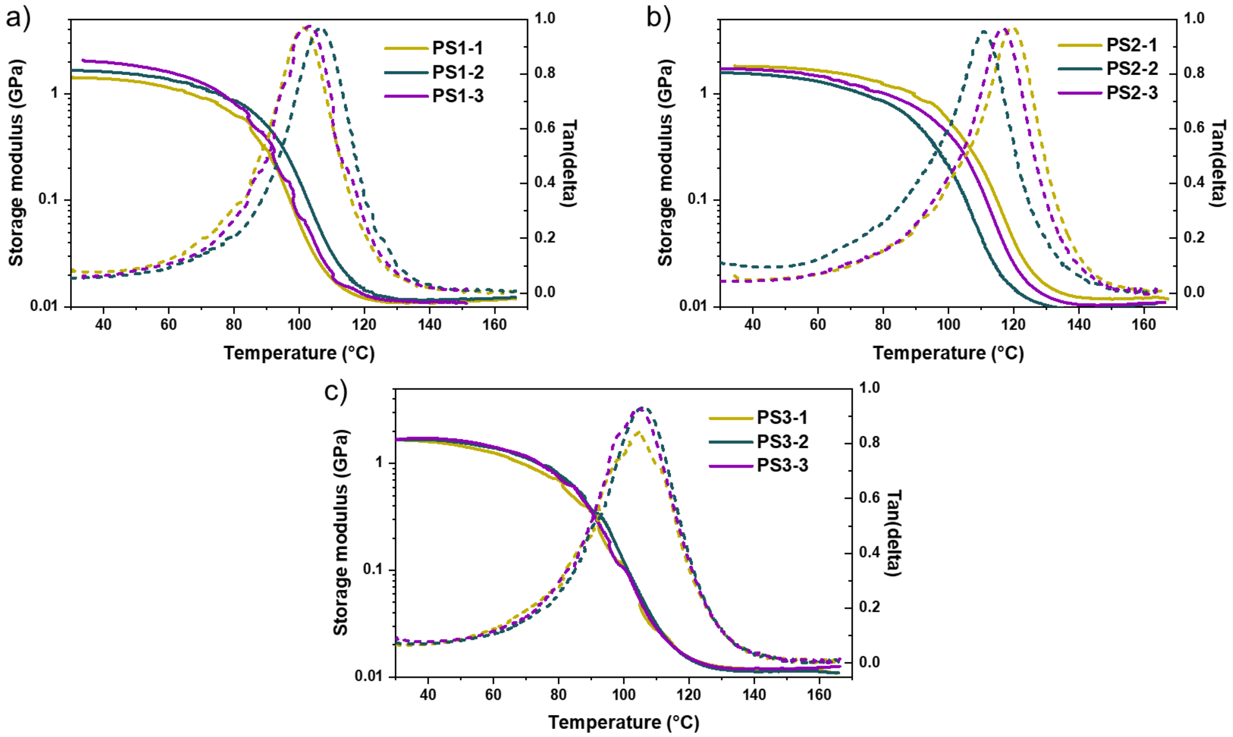

Figure S12. Dynamic mechanical analysis (n = 3) of rectangular samples (30 mm × 5 mm × 2 mm), showing the storage modulus/tan(delta) - temperature trace of (a) photosystem 1, (b) photosystem 2, and (c) photosystem 3. Samples were printed with 365 nm light, washed with isopropyl alcohol and ethyl acetate, then dried under vacuum. Samples labeled as PSX-n (photosystems X = 1-3, trial number n = 1-3)

Table S10. Glass transition temperature (T_g) and crosslinking density obtained by triplicating.

Sample name	T_g (°C)*	Plateaued E' (MPa)**	Crosslinking density ν (mol/mm^3)***
**PS1**	104 ± 2	11.3 ± 0.3	(1.09 ± 0.03) ×10^{-6}
**PS2**	116 ± 3	10.7 ± 0.9	(1.04 ± 0.09) ×10^{-6}
**PS3**	105.3 ± 0.6	11.7 ± 0.3	(1.16 ± 0.03) ×10^{-6}

*T_g represents the tan(delta) peak temperature in the DMA trace.
**Plateau modulus represents an average temperature between 130-140 °C.
***The crosslinking density was calculated using an affine network theory.

Affine network theory. The crosslinking density was calculated using affine network theory:

$$\nu_c = \frac{E'}{3RT} \quad \textbf{(Equation S1)}$$

where ν_c is the crosslinking density (mol/m^3), E' is the rubbery storage modulus measured by DMA (Pa), R is the gas constant (8.314 J/mol·K), T is the average absolute temperature (K) where the rubbery storage modulus plateau was taken

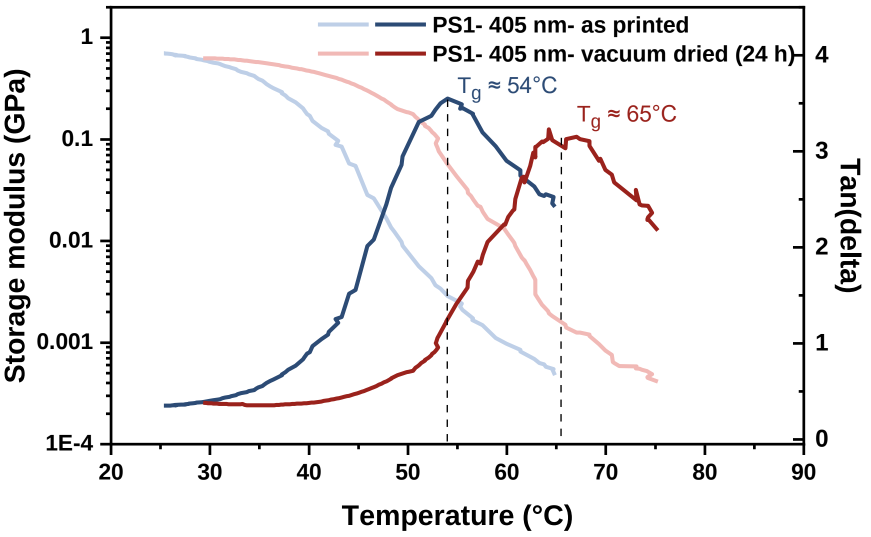

Figure S13. Representative trace of dynamic mechanical analysis on a rectangular sample 3D printed using photosystem 1 irradiated with 405 nm light, measured as printed (blue line) and after vacuum drying for 24 hours at room temperature (red line), providing a glass transition peak at around 60 °C.

Table S11. Glass transition temperature (T_g) of dissolvable support material (rectangular shape) prepared using photosystem 1 and 405 nm 3D printing and measured using DMA.

Sample name	T_g (°C)
**PS1-1**	54
**PS1-2**	61
**PS1-3**	59
**PS1-4 (dried)**	65
**Average**	60 ± 4

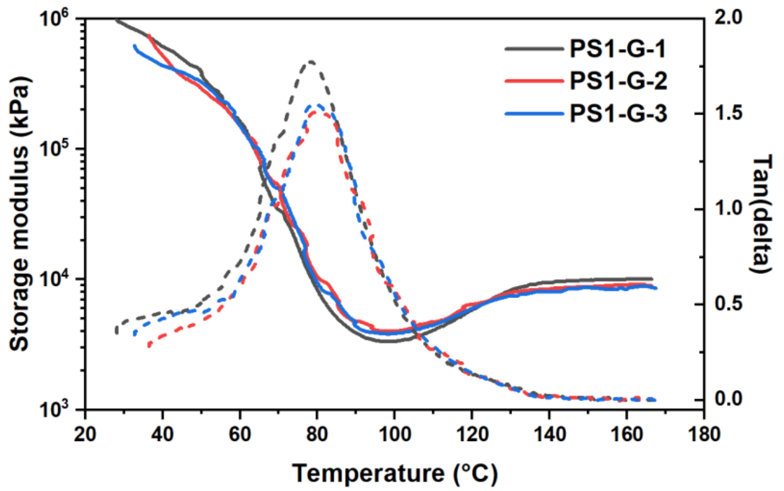

Figure S14. Dynamic mechanical analysis (DMA) of rectangle samples PS1-G-n (n = 1-3) as printed using 365 nm light (green bodies). Samples were lightly rinsed with isopropanol and air dried prior to testing.

Table S12. Glass transition temperature (T_g) of PS1 samples as printed using 365 nm light (green bodies), reported as the tan(delta) peak temperature in the DMA traces. The average value and standard deviation (STD) is also provided.

**Sample name**		**T_g (°C)**
**PS1 365 nm as printed**	**PS1-G-1**	78
	**PS1-G-2**	80
	**PS1-G-3**	80
	**Average**	**79 ± 1**

S2.3. Thermogravimetric Analysis

Thermogravimetric analysis. TGA was carried out for samples prepared with either photosystem 1 or photosystem 2 irradiated using 365/405 nm or 365/460 nm configurations, respectively. A lack of mass loss prior to 100 °C indicates effective removal of residual solvent with our drying process. Mass loss prior to ~300 °C is attributed to residual monomer or oligomeric species.

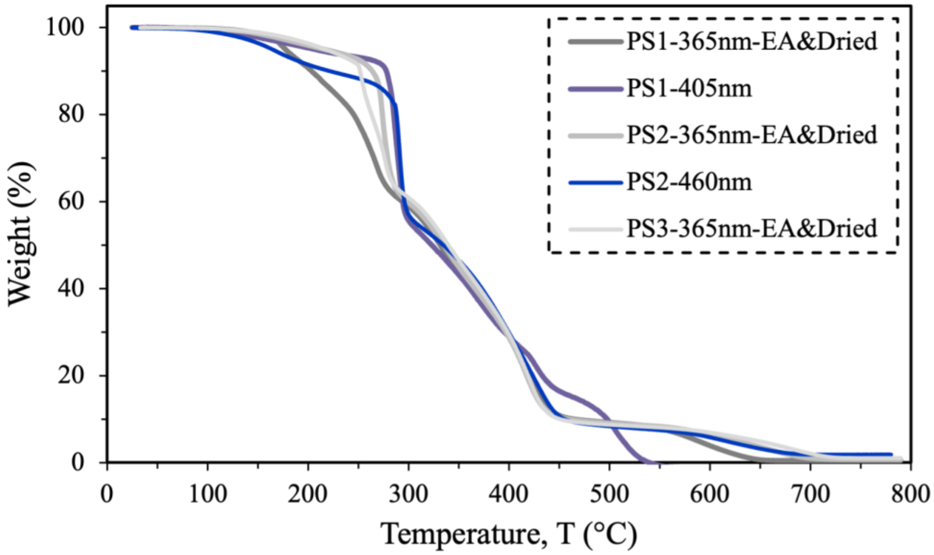

Figure S15. Thermogravimetric analysis of samples printed with photosystem 1, 2, or 3. Samples printed with 365 nm were washed with ethyl acetate for 20 minutes and then dried at 100 °C for 24 hours prior to testing. Samples printed with 405 or 460 nm light were rinsed with IPA and then air dried for 24 hours prior to testing.

S2.4. Dissolution Studies

Gravimetric analysis was accomplished to characterize dissolution over time for 3D printed samples placed in ethyl acetate. The dissolution of visible light cured structures was characterized using a representative support structure (14 mm diameter, 0.75 mm base thickness, 0.75 mm support diameter) with two photosystems: photosystem 1 using 405 nm, 4 s/50 µm layer and photosystem 2 using 460 nm, 6 s/50 µm layer at 50 mW/cm^2 (**Figure S15**). UV cured disks (14 mm diameter, 2 mm thickness) were also prepared with photosystems 1 and 2 using 365 nm, 4 s/50 µm layer at 50 mW/cm^2 (**Figure S15**). Each time point was collected in triplicate. The initial weight of each sample was collected after rinsing with isopropyl alcohol to remove any excess resin and air drying in the absence of light. Each sample was placed in a 20 mL vial filled with 15 mL ethyl acetate, capped, and agitated on a shaker at 300 rpm at room temperature for each specified time point. Solvent was then decanted off and the vial was rinsed with ethyl acetate. Vials were then dried uncapped in a vacuum oven at 100 °C for 24 hours and a final weight was collected to determine change in mass.

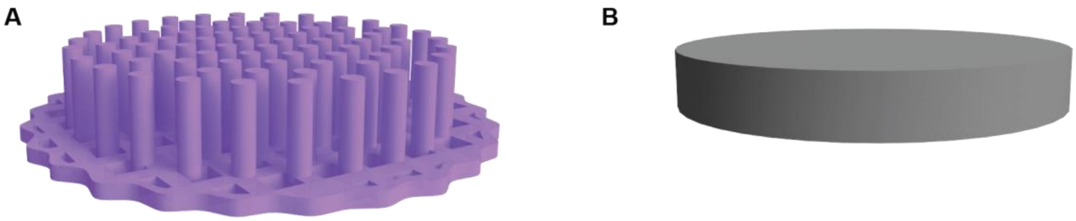

Figure S16. Digital renderings of 3D printed structures used for dissolution studies, where the structures are representative of (a) visible light cured supports that would be dissolved from a multi-material object and (b) a UV cured component that would remain after support removal.

S2.5. Molecular Weight Characterization

A series of resins were prepared using photosystem 1 with a range of EVS concentrations from 0 to 2 mol%, printing with 405 nm light. Increasing the EVS loading resulted in a decrease in the number average molecular weight (M_n) (**Table S12**). In decreasing M_n from ~39 kDa to ~9 kDa the rate of support dissolution in ethyl acetate qualitatively increased. Notably, the resins containing 0 and 0.25 mol% EVS produced objects that did not fully dissolve in ethyl acetate after soaking for ~24 hours, but did result in considerable swelling (**Figure S17**).

Table S13. Gel permeation chromatography results of samples that were printed using photosystem 1 (405nm, 50 mW/cm^2) and then placed in ethyl acetate for 24 hours. Molecular weight and dispersity determined relative to polystyrene standards.

EVS Loading (mol%)	M_n (kDa)	*Đ*
0.00	33.8	2.63
0.25	39.4	4.81
0.50	28.2	4.73
1.00	16.5	2.60
2.00	10.6	2.10

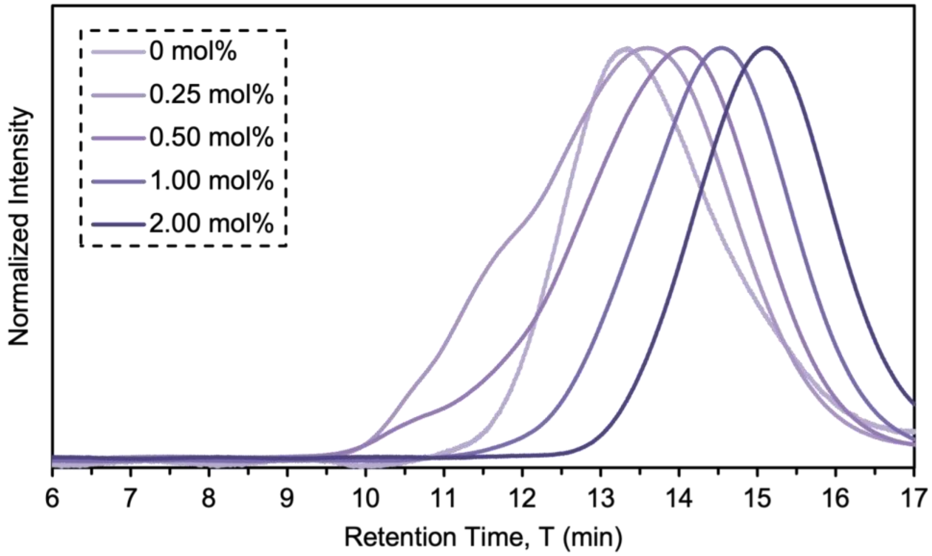

Figure S17. Gel permeation chromatography traces for samples that were printed using photosystem 1 (405nm, 50 mW/cm^2) and then placed in ethyl acetate for 24 hours.

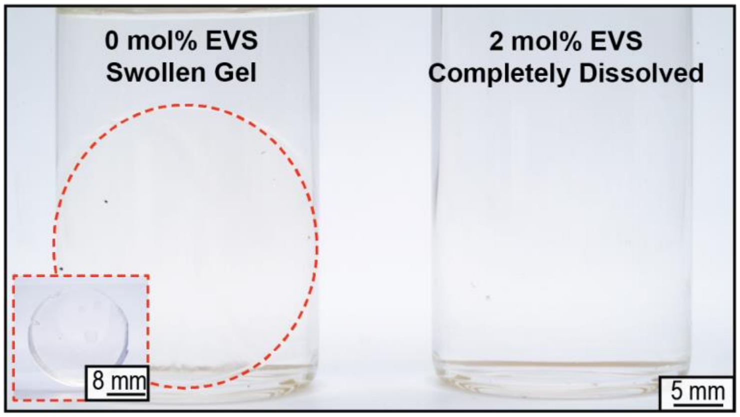

Figure S18. Representative images of samples that were printed using photosystem 1 (405 nm, 50 mW/cm^2) and then placed in ethyl acetate for 24 hours. The dashed red line has been added to draw the eye to the swollen disk for the sample prepared in the absence of EVS. Inset: image of swollen disk removed from the vial for the sample prepared with no EVS present in the resin.

S2.6. Gel Fraction of 3D Printed Thermosets

Rectangular samples (35 mm × 10 mm × 1.5 mm, length × width × thickness) were 3D printed using the optimized resin formulation and process parameters; 4 s per 50 µm layer at an intensity of 50 mW/cm^2. Four samples each were printed using a 365 nm exposure (**Figure S18**). The initial weight (W_0) of each as-printed sample was collected after rinsing the surface with isopropyl alcohol (IPA) and briefly drying in air to evaporate surface solvent. The percentage of sol (i.e., residual monomer) was determined by washing samples in ethyl acetate, a good solvent that led to a high swelling ratio (((swollen mass – dry mass)/(dry mass))×100 ≈ 53 ± 8% w/w), to extract out components that were not covalently bound to the polymer network (i.e., sol fraction, including residual monomer and photosystem components). After soaking the samples for ~12 hours in ethyl acetate, with three solvent exchanges during that time, we dried each at 100 °C for 12 hours, then recorded the final weight (W_f). Notably, 12 hours was found to be sufficient to fully remove solvent (**Figure S19**). From this method, the gel fraction ((W_f/W_0)×100) for 365 nm printed samples was found to be 86.9 ± 0.3%, 90.7 ± 0.2%, and 87.1 ± 0.3%, for PS1, PS2, and PS3 respectively.

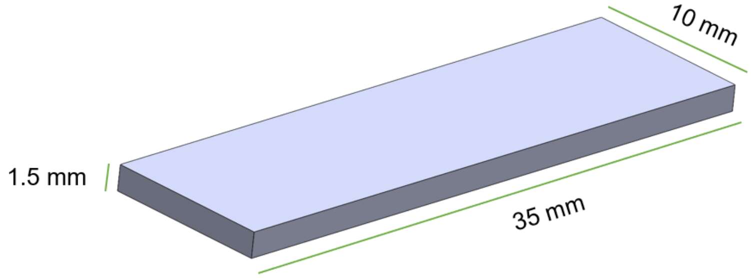

Figure S19. Digital rendering of 3D printed rectangular bars used to determine sol-gel fractions.

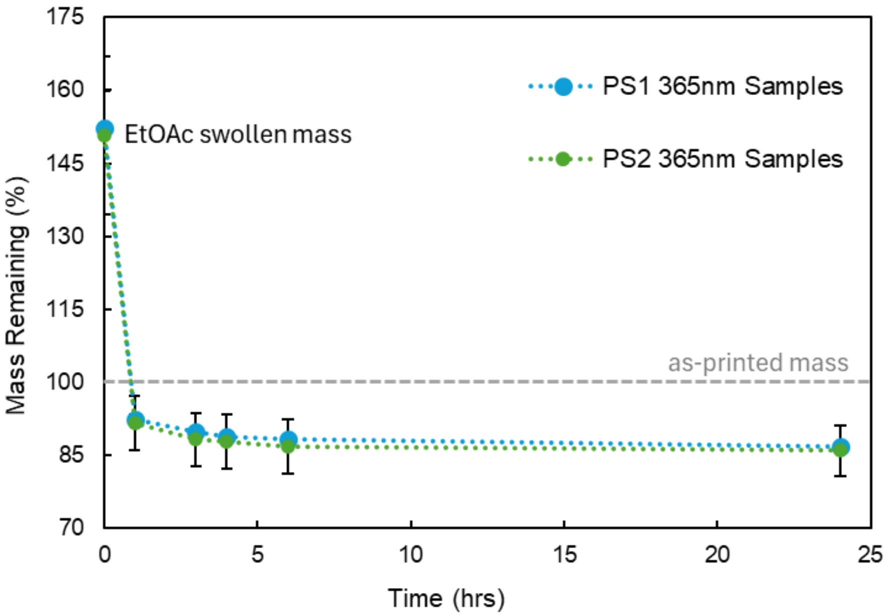

Figure S20. 3D printed samples were soaked in ethyl acetate for 12 hours then placed in a vacuum oven at 100 °C, removing and weighing periodically to determine the time required to fully remove solvent.

Table S14. Summary of swelling analysis. Pre-weighed samples were soaked in ethyl acetate for 12 hours and weighed again. Swelling was calculated as the ratio of solvent uptake weight to the dry polymer weight shown as a percentage.

**PS1 (MeOTX + BAPO)**					
Swelling (%)				**Avg**	**Std**
R1	R2	R3	R4		
45	67	51	49	**53**	**8**
**PS2 (ITX + Ivocerin)**					
Swelling (%)				**Avg**	**Std**
R1	R2	R3	R4		
39	48	56	62	**51**	**9**
**PS3 (MeOTX + Ivocerin)**					
Swelling (%)				**Avg**	**Std**
R1	R2	R3	R4		
57	43	60	62	**55**	**7**

Table S15. Summary of sol-gel analysis. Pre-weighed samples were soaked in ethyl acetate for 12 hours with three solvent exchanges and then vacuum dried at 100 °C for 12 hours, then weighed.

**PS1 (MeOTX + BAPO)**					
Gel Fraction (%)				**Avg**	**Std**
R1	R2	R3	R4		
86.8	86.5	86.6	87.5	**86.9**	**0.3**
**PS2 (ITX + Ivocerin)**					
Gel Fraction (%)				**Avg**	**Std**
R1	R2	R3	R4		
91.0	90.9	90.5	90.7	**90.7**	**0.2**
**PS3 (MeOTX + Ivocerin)**					
Gel Fraction (%)				**Avg**	**Std**
R1	R2	R3	R4		
86.6	87.2	87.2	87.3	**87.1**	**0.3**

S2.7. Viscosity Measurements

Rheology was performed on solutions containing 3D printed samples dissolved in diglyme (sample concentration = 9% w/w in diglyme), used as a proxy solvent for ethyl acetate owing to its high boiling point to mitigate evaporation during measurement. Reference sample disks (2-mm thick and 7-mm in diameter) were 3D printed using 405 nm light (PS1) or 460 nm light (PS2 and PS3), followed by dissolution in diglyme overnight. Viscosity was obtained using a flow sweep mode in a parallel plate geometry.

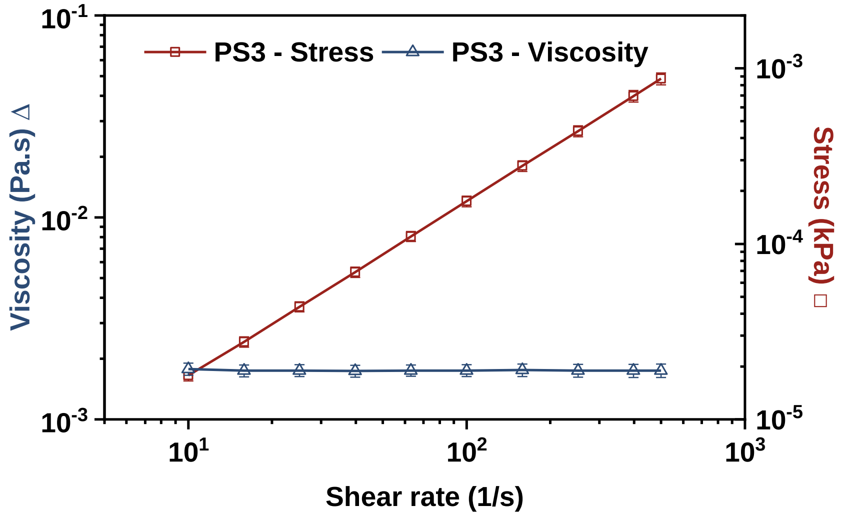

Figure S21. Representative trace of flow sweep measurements performed on rheometer with a PS3 460 nm 3D printed sample dissolved into diglyme.

Table S16. Summary of rheology results for 405 nm and 460 nm samples dissolved in diglyme.

**PS1 (MeOTX + BAPO)**				
Viscosity (cP)			**Avg**	**Std**
R1	R2	R3		
1.92	1.57	1.81	**1.77**	**0.14**
**PS2 (ITX + Ivocerin)**				
Viscosity (cP)			**Avg**	**Std**
R1	R2	R3		
1.51	1.52	1.54	**1.52**	**0.01**
**PS3 (MeOTX + Ivocerin)**				
Viscosity (cP)			**Avg**	**Std**
R1	R2	R3		
1.69	1.65	1.90	**1.75**	**0.11**

S2.8. Support Design

Supports were designed utilizing Netfabb support generation (see S1.4.4 for more details) by keeping a fixed number of summers (anchor distance) and varying the support size to change the fill density (**Table S16**). The various fill densities were tested empirically by printing a UV (365nm) disk (14 mm × 2 mm) containing the dissolvable supports and one dissolvable interfacial layer. Samples with fill densities as low as 14.3% appeared visually the same as those with 39.7% fill densities (**Figure S21**). Furthermore, surface roughness (R_a) values for all samples were comparable (see S2.11 for more details). For more complex objects or prints, a user should consider the aspect ratio and size of the printed object to avoid support distortions or collapse.

Table S17. Fill density for varying support widths from 600-1000 µm for samples printed using photosystem 1.

Support Width (µm)	Fill Density (%)
600 µm	14.3
750 µm	22.3
850 µm	28.6
1000 µm	39.7

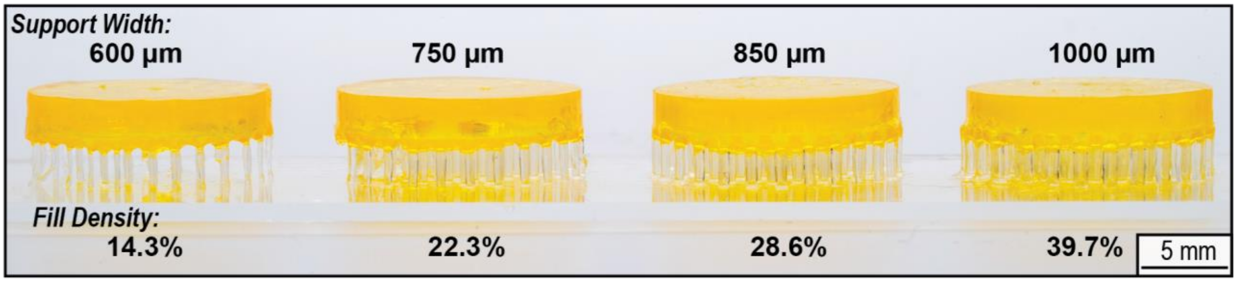

Figure S22. Samples printed with varying support width and fill densities (see **Table S16**) utilizing photosystem 1.

S2.9. Angled Supports

Supports were designed utilizing Netfabb support generation (see S1.4.4 for more details) with 750 µm supports. The print angle was modified from 0° to 45° with one dissolvable interfacial layer between supports and the UV light (365nm) cured disk (14 mm × 2 mm). Qualitatively, the samples appeared similar, while surface roughness (R_a) values were quantitatively similar for all print angles (**Figure S22-23**).

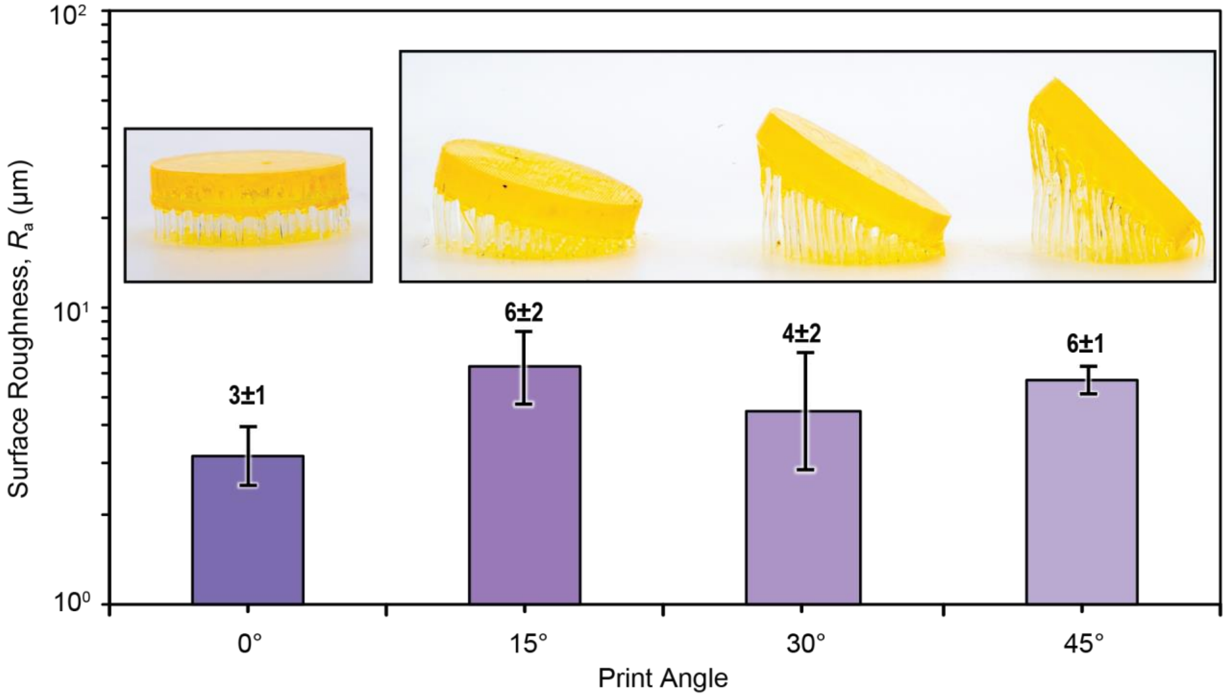

Figure S23. Surface roughness measurements and optical images of dissolvable support surfaces for samples printed using the 365nm/405nm setup with PS1, 750 µm supports, and one interface layer.

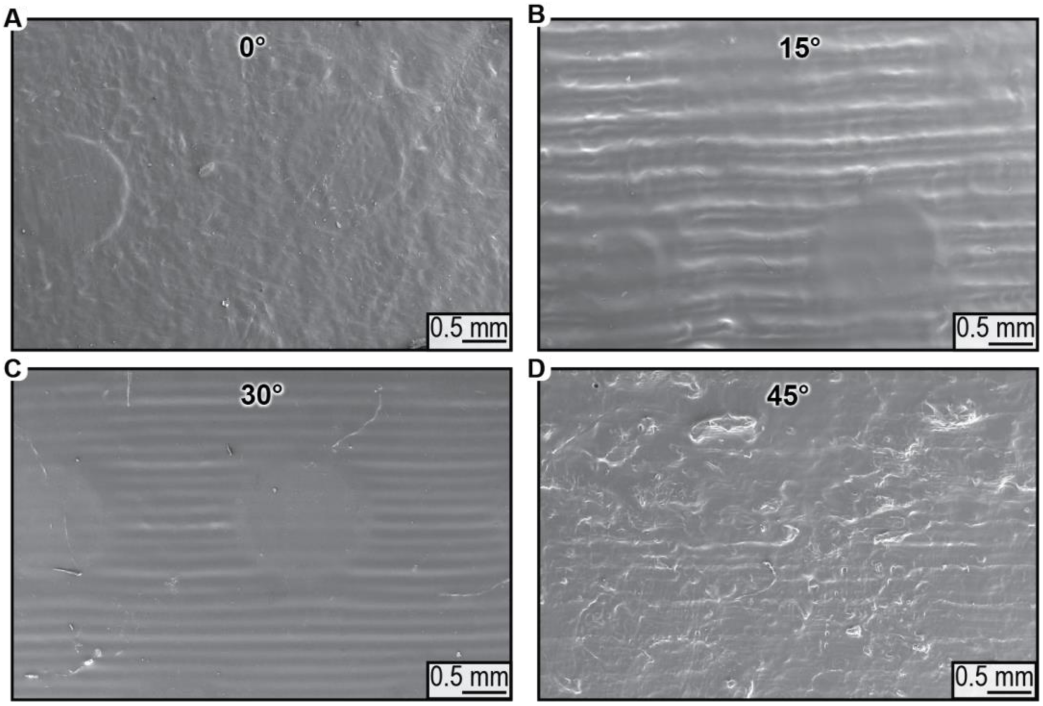

Figure S24. SEM images of dissolvable support surfaces for samples printed at 365nm/ 405nm with PS1, 750 μm supports, and one interface layer at (A) 0°, (B) 15°, (C) 30°, and (D) 45°.

S2.10. Mechanical Properties

A comprehensive report of the tensile testing results are provided below. Representative traces and photos of dogbones are included in **Fig. 3A** of the main manuscript.

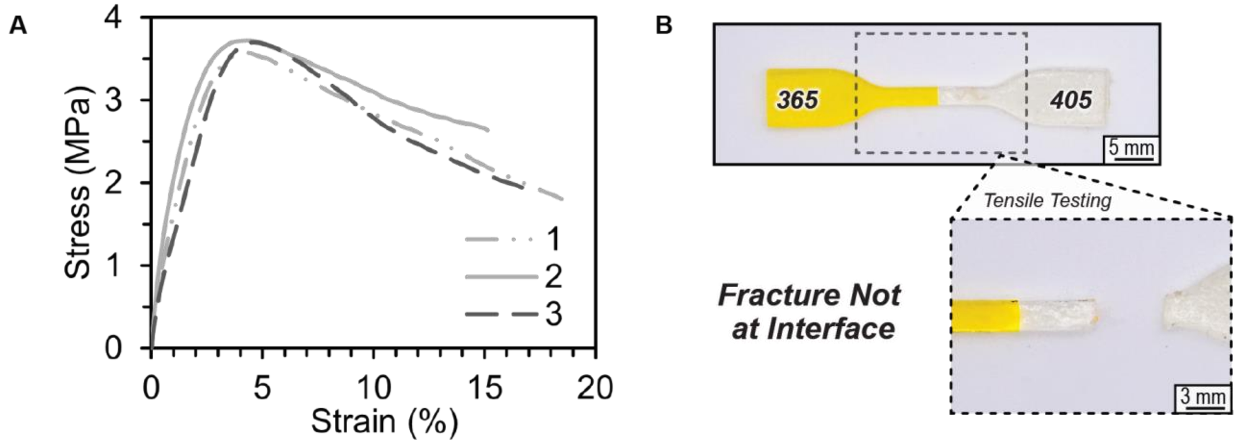

Figure S25. Mechanical testing of multimaterial dogbones prepared using photosystem 1 resin to characterize interfacial bonding between dissolvable (405 nm) and non-dissolvable (365 nm) domains. (A) Stress-strain plot from uniaxial tensile testing. (B) Image of half-and-half dogbone before and after tensile testing showing fracture occurring away from the interface. Dogbone samples were printed with one half exposed to 365 nm light and the other half to 405 nm light (50 mW/cm^2 , 4 s/50 µm layer).

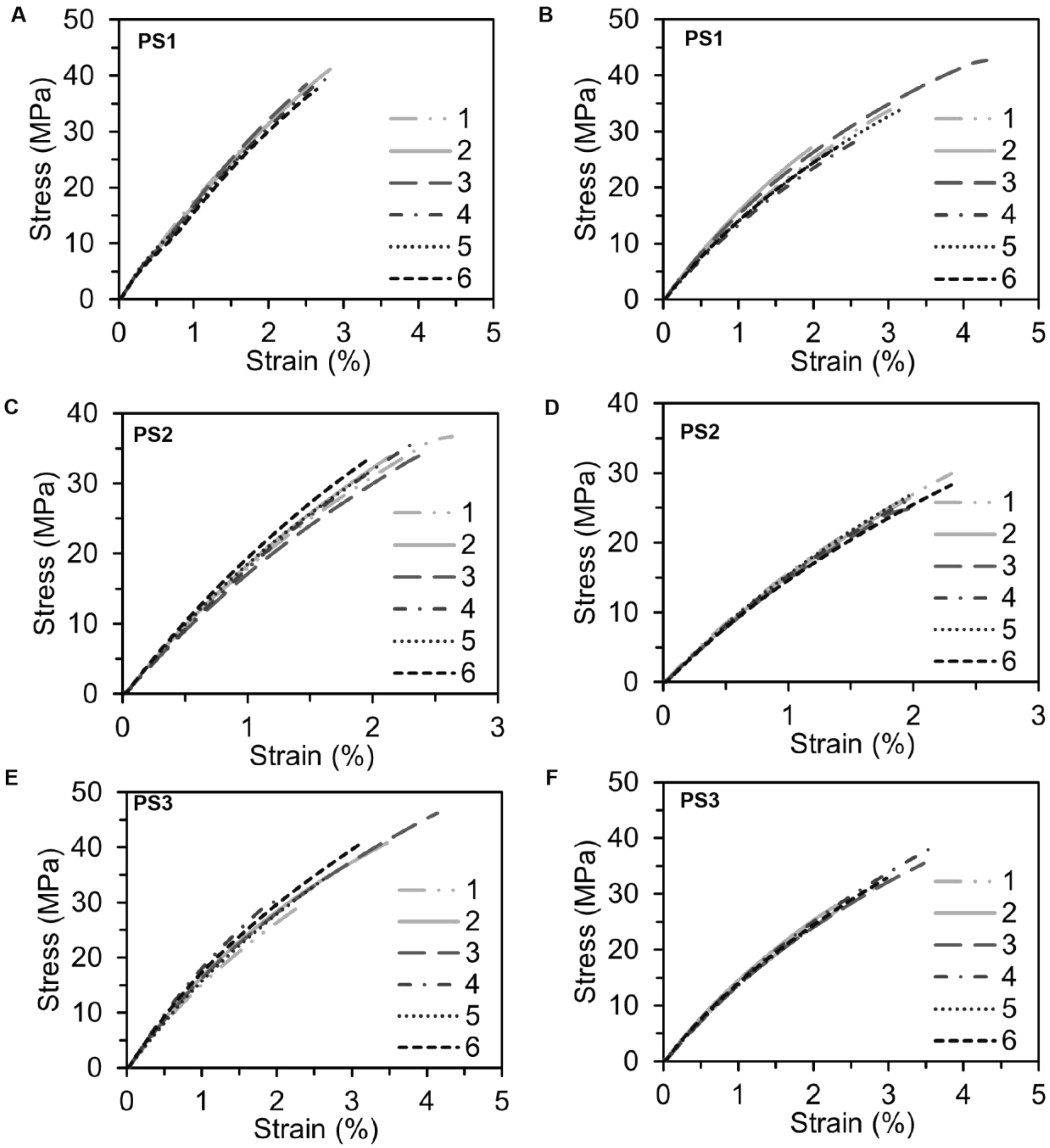

Figure S26. Stress-strain plots from tensile testing dogbone samples prepared using 365 nm light (50 mW/cm^2, 4 s/50 µm layer). All samples were rinsed with isopropyl alcohol immediately after printing to remove residual surface resin and then dried under reduced pressure at 100 °C for 24 hours prior to tensile testing. (a) Photosystem 1 samples that were not soaked in ethyl acetate. (b) Photosystem 1 samples that were soaked in ethyl acetate for 20 minutes before drying. (c) Photosystem 2 samples that were not soaked in ethyl acetate. (d) Photosystem 2 samples that were soaked in ethyl acetate for 20 minutes before drying. (e) Photosystem 3 samples that were not soaked in ethyl acetate. (f) Photosystem 3 samples that were soaked in ethyl acetate for 20 minutes before drying.

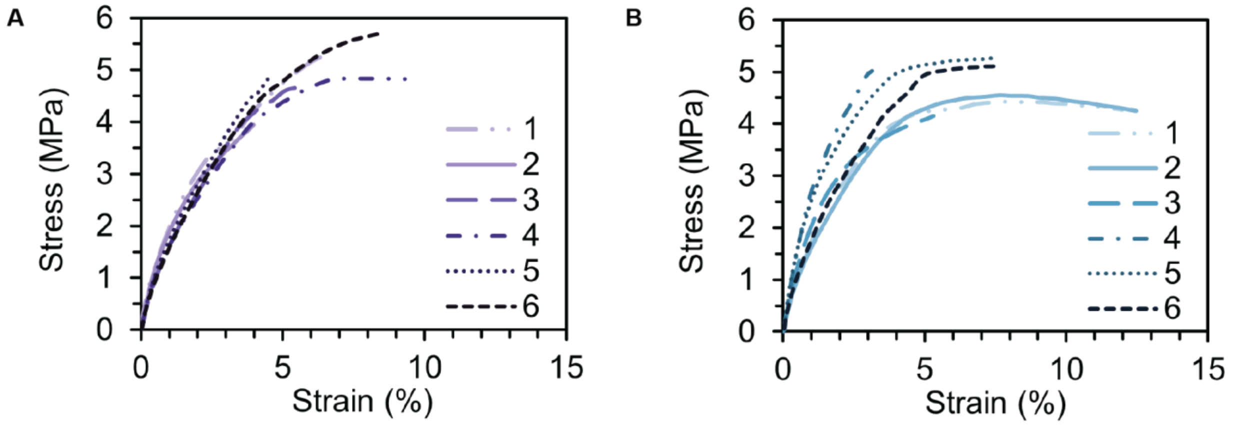

Figure S27. Stress-strain plots from tensile testing dogbone samples prepared using visible light. (a) 405 nm, 50 mW/cm^2, 4 s/50 µm layer. (b) 460 nm, 50 mW/cm^2, 6 s/50 µm layer.

Table 18. Summary of tensile Young's moduli, maximum stress at break, and maximum strain at break for 3D printed samples.

Photosystem	Wash	Wavelength (nm)	Young's Modulus	Max Stress (MPa)	Max Strain (%)
1	None	365	1.60 ± 0.07 GPa	36 ± 5	2.7 ± 0.4
1	20 min EtOAc	365	1.24 ± 0.15 GPa	32 ± 0.8	3.2 ± 0.8
1	N/A	405	162 ± 36 MPa	4.9 ± 0.6	6.5 ± 2
2	None	365	1.44 ± 0.11 GPa	34 ± 2	2.5 ± 0.3
2	20 min EtOAc	365	1.17 ± 0.11 GPa	27 ± 2	2.3 ± 0.2
2	N/A	460	201 ± 47 MPa	4.9 ± 0.6	7.4 ± 2
3	None	365	1.48 ± 0.15 GPa	37 ± 7	3.2 ± 0.8
3	20 min EtOAc	365	1.26 ± 0.13 GPa	32 ± 5	3.1 ± 0.6

S2.11 SEM of Dried Samples

Following tensile testing where a drop in tensile modulus for thermoset parts was observed after washing and drying, SEM imaging of cross-sections was conducted. This revealed a morphological change with 'green' samples appearing more dense than corresponding washed and dried samples as indicated by the presence of voids throughout the polymer matrix (Fig. S28).

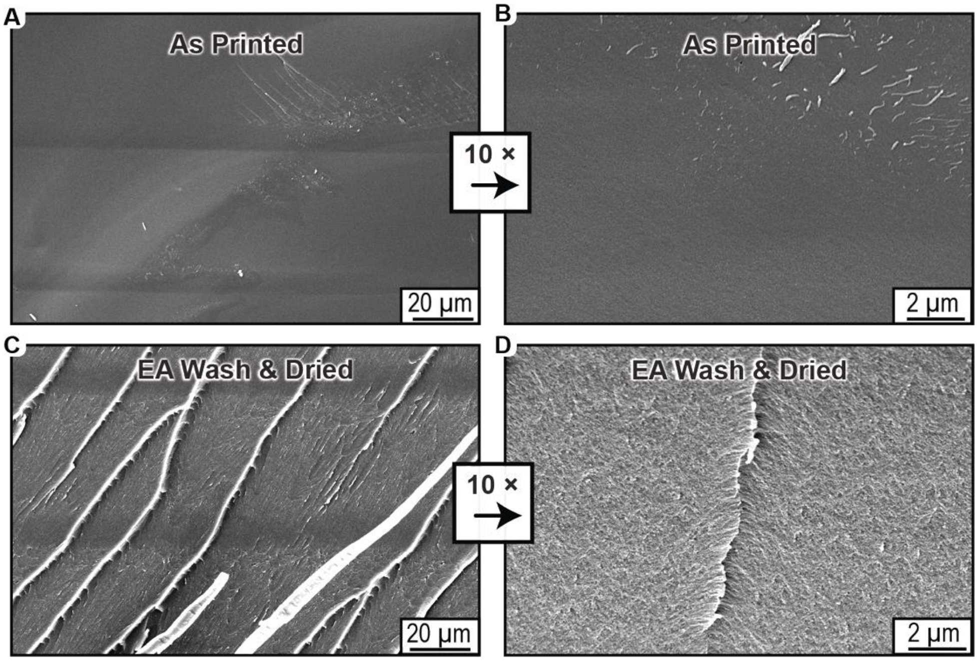

Figure S28. SEM images of sample cross-sections for 'green body' (as printed) and ethyl acetate washed (20 min.) and dried (100 °C in vacuo, 24 hours) samples that were prepared using 365 nm printing (photosystem 1). Notably changes in morphology from post-processing were observed. In both samples at ~2500× magnification, the print layer lines can be visualized by horizontal dark regions.

S2.12. Surface Roughness

Manual support removal of UV printed monoliths was conducted using a Formlabs finishing kit following their support removal guidelines.[S4,S5] Subsequently, contact profilometry was carried out according to S1.4.9 and representative profiles are provided in **Figures S27-S30** below. The average surface roughness (R_a) was determined to assess the surface finish after manual removal steps and after dissolvable support washing. The results are summarized in **Table S18**. Notably, a single interface layer was found to improve the surface finish as evidenced by a decrease in R_a.

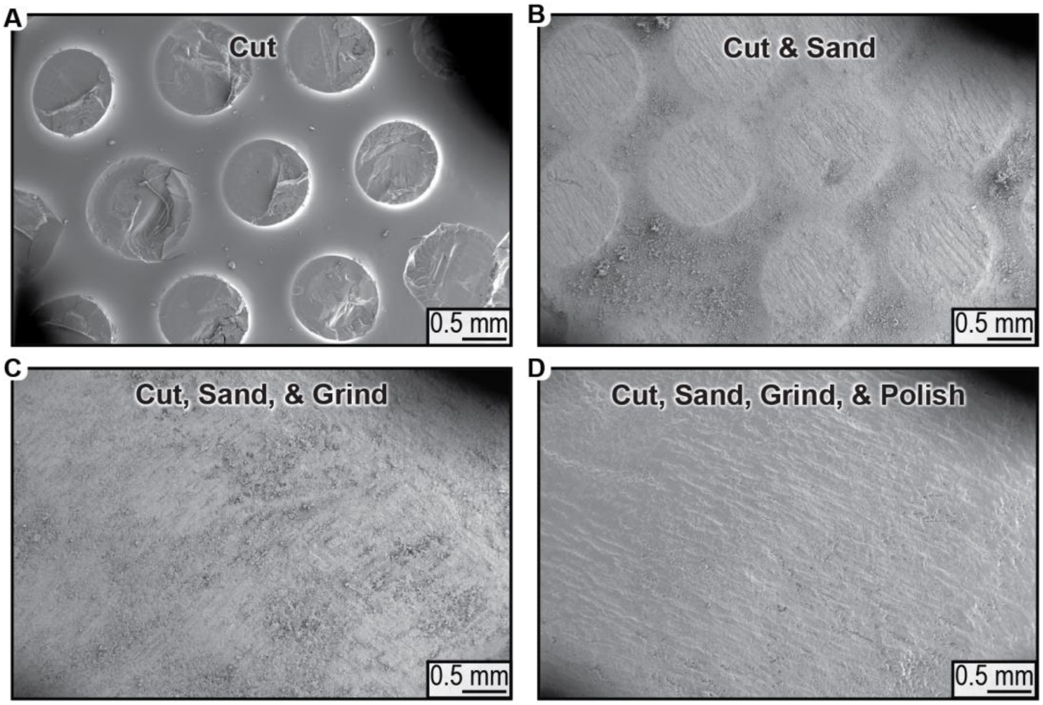

Figure S29. SEM images of monolithic disk prints at different stages of manual support removal with (A) support cutting, (B) cutting & sanding, (C) cutting, sanding, & grinding, and (D) cutting, sanding, grinding, & polishing. Prints were prepared using photosystem 1 and 365 nm light exposure with 750 µm supports.

Table S19. Summary of surface roughness values for 3D printed disk samples with manual or dissolvable support removal.

Photosystem	Support Width/ Angle	No. of Interfacial Layers	Support Removal	Surface Roughness, R_a ($\mu m \pm \sigma$)
1	750 µm/ 0°	N/A	Manual: Cut	37 ± 9*
1	750 µm/ 0°	N/A	Manual: Cut & Sand	19 ± 6
1	750 µm/ 0°	N/A	Manual: Cut, Sand, & Grind	6 ± 1
1	750 µm/ 0°	N/A	Manual: Cut, Sand, Grind, & Polish	5 ± 1
1	600 µm/ 0°	0	Dissolvable	10 ± 1
1	600 µm/ 0°	1	Dissolvable	3 ± 1
1	750 µm/ 0°	0	Dissolvable	8 ± 1
1	750 µm/ 0°	1	Dissolvable	3 ± 1
1	850 µm/ 0°	1	Dissolvable	4 ± 1
1	1000 µm/ 0°	1	Dissolvable	4 ± 1
2	600 µm/ 0°	1	Dissolvable	5 ± 1
2	750 µm/ 0°	1	Dissolvable	4 ± 2
2	850 µm/ 0°	1	Dissolvable	3 ± 2
2	1000 µm/ 0°	1	Dissolvable	5 ± 2
1	750 µm/ 15°	1	Dissolvable	6 ± 2
1	750 µm/ 30°	1	Dissolvable	4 ± 2
1	750 µm/ 45°	1	Dissolvable	6 ± 2

*Average surface roughness for manual removal of supports after the first stage (cutting) did not account for the large remaining support features, only the spaces in-between

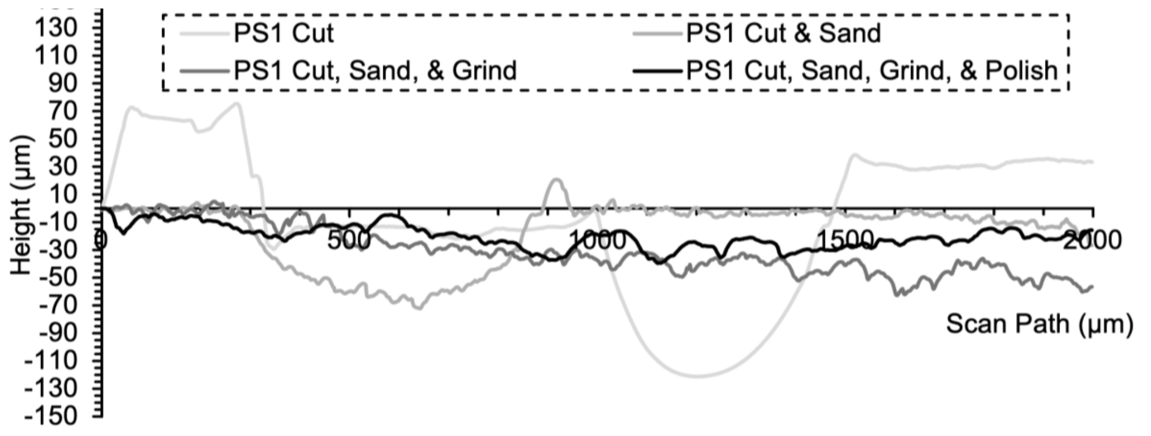

Figure S30. Representative surface profiles for photosystem 1 samples with manual support removal at each consecutive stage.

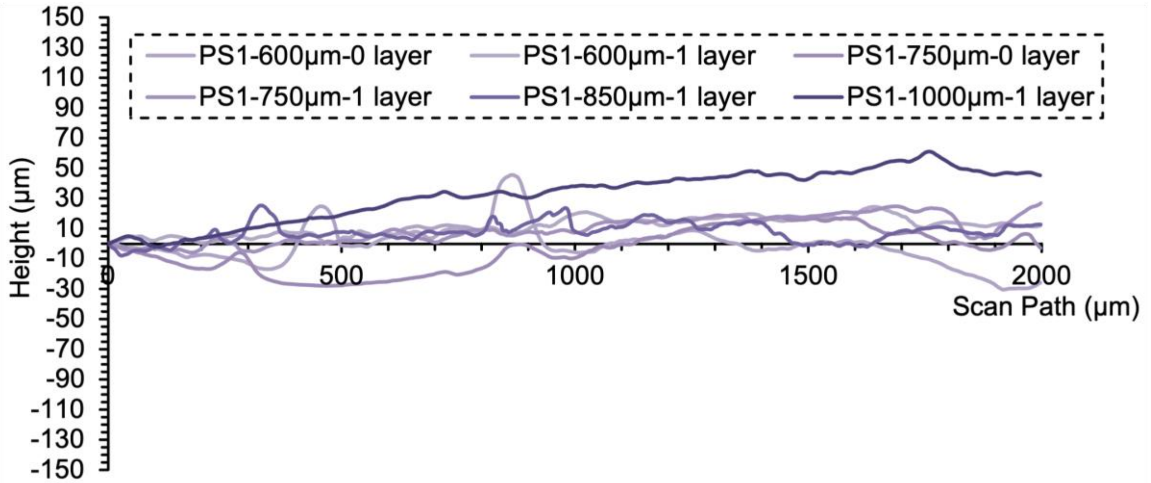

Figure S31. Representative surface profiles for photosystem 1 samples with dissolvable support removal, varying support diameter from 600-1000 µm with and without one interfacial layer of dissolvable material.

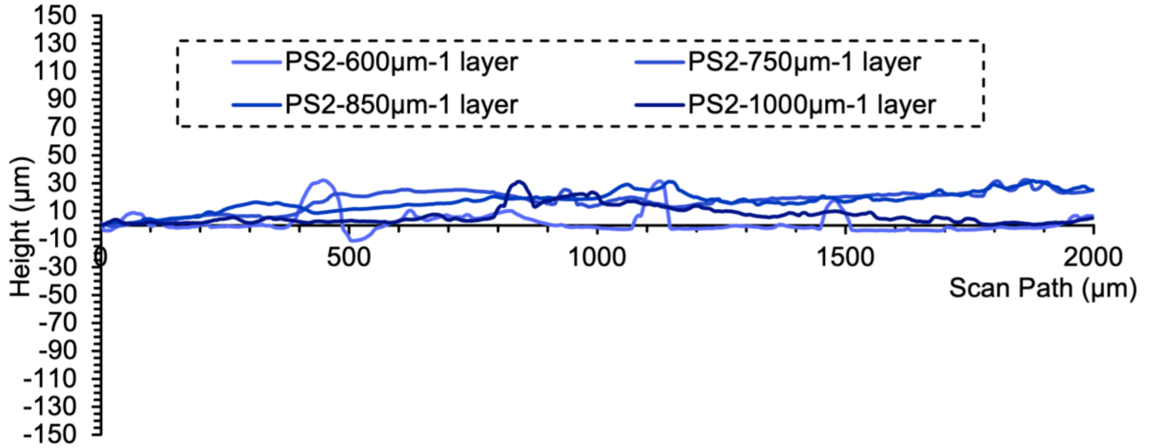

Figure S32. Representative surface profiles for photosystem 2 samples with dissolvable support removal, varying support diameter from 600-1000 µm with and without one interfacial layer of dissolvable material.

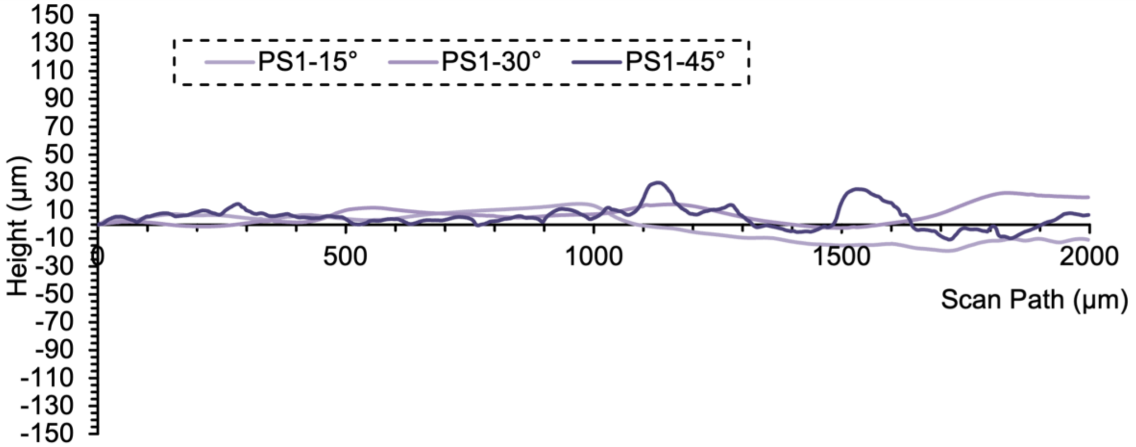

Figure S33. Representative profiles for photosystem 1 samples with dissolvable support removal, varying the angle of 750 µm supports connected to one interfacial layer of dissolvable material.

S2.13. Supplemental Resolution Prints

"Test your printer" print. The following print file (**Figure S31**) was used to qualitatively identify the fine and complex details that was possible with UV curing of the thermoset. The print was sourced and used under creative commons license from an open source print file sharing website, Thingiverse.[S6] The STL was scaled uniformly to achieve a 15 mm × 25 mm base size (~0.5× the original size). Prints were accomplished using a 365 nm exposure time of 4 seconds/50 µm and a light intensity of 50 mW/cm^2.

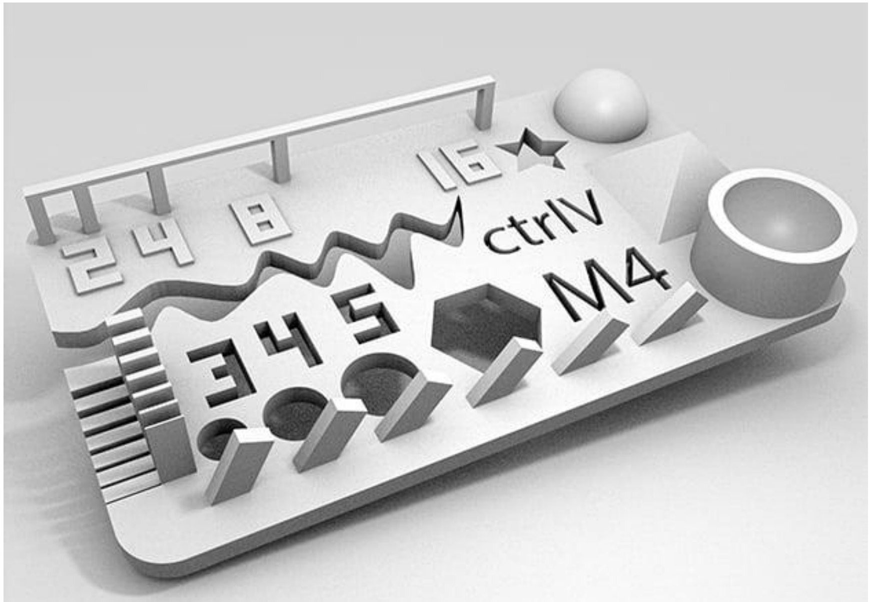

Figure S34. Digital rendering of the "Test your 3D Printer" STL file.

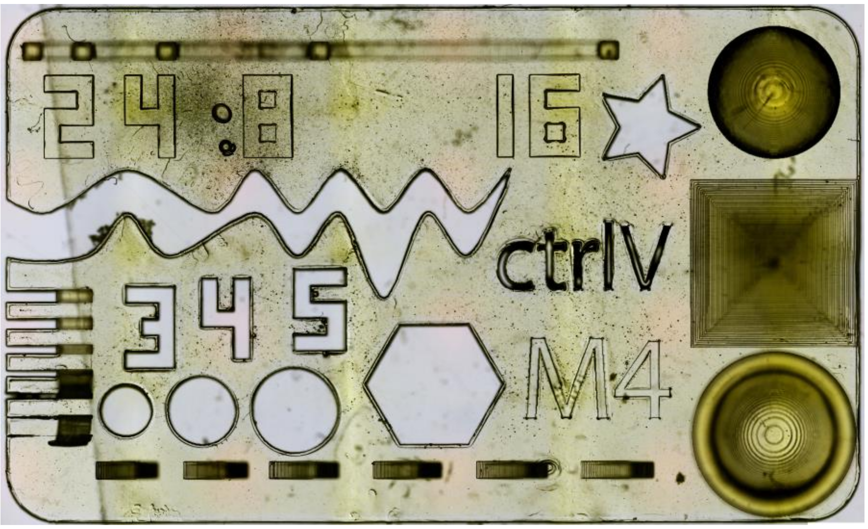

Figure S35. Stitched optical microscope image of a "Test your printer" print prepared using photosystem 2. Image taken using backlighting. Total print dimensions are ~15 mm × 25 mm.

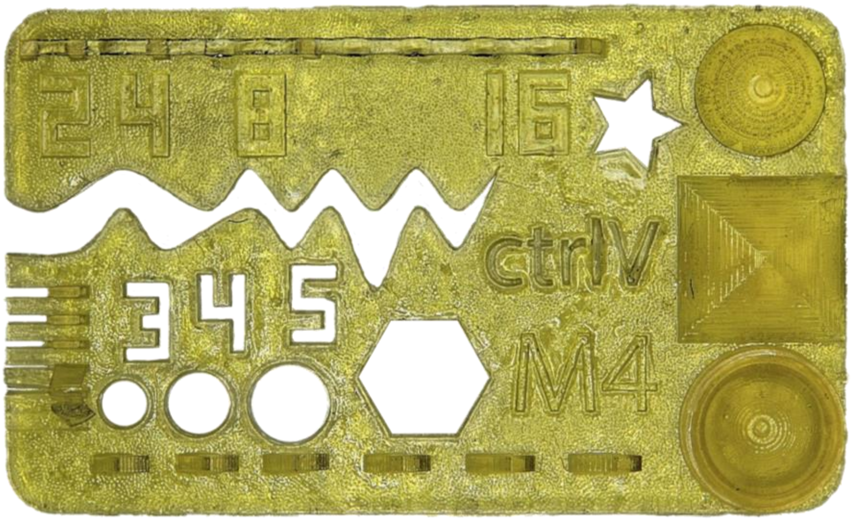

Figure S36. Digital microscope image of a "Test your printer" print prepared using photosystem 1, after dissolvable support removal that were originally added to support the sensitive bridges. Image taken using top-lighting. Total print dimensions are ~15 mm × 25 mm.

Print with pillars of variable size. The following print file (**Figures S34-S35**) was used to qualitatively identify the fine details that were achievable for the UV cured thermoset. The print was designed in SolidWorks CAD software. Prints were accomplished using a 365 nm exposure time of 4 seconds/50 µm and a light intensity of 50 mW/cm^2.

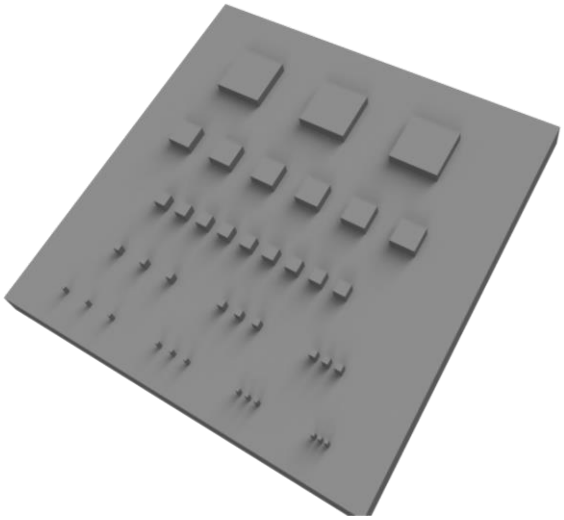

Figure S37. 3D digital rendering of the STL file containing pillars of varying size.

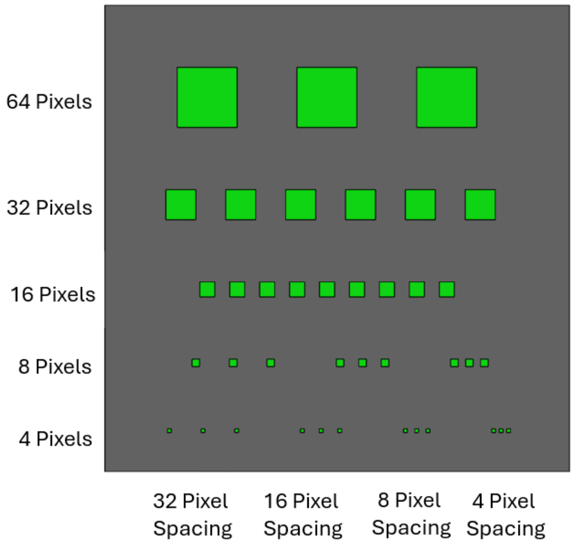

Figure S38. Digital rendering of the STL file containing pillars of varying size with dimensions noted as factors of the pixel size (15.1 µm).

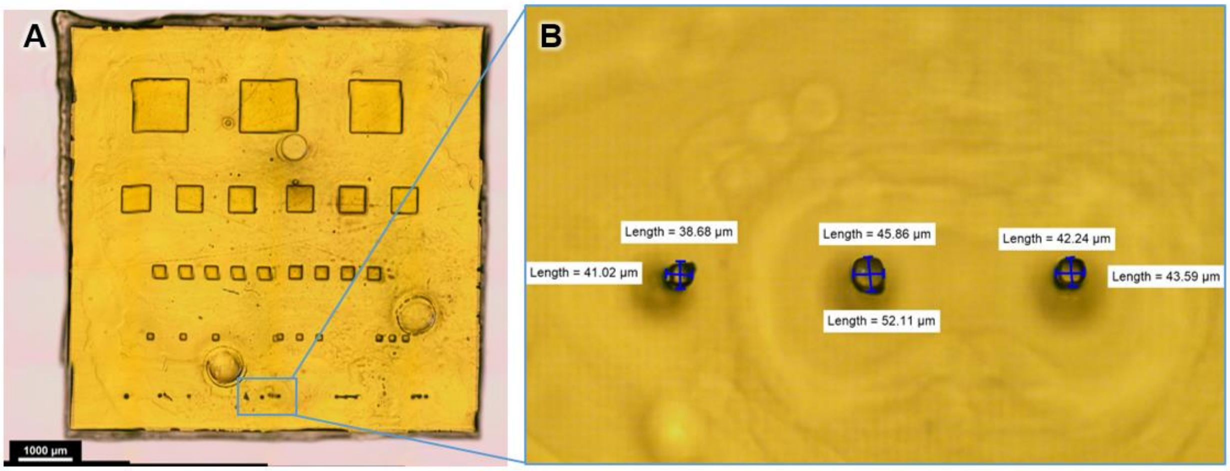

Figure S39. Optical microscope images of the print containing pillars of varying size using photosystem 1 after 365 nm exposure. Images taken using top-lighting. Total print dimensions are 7.5 mm × 7.5 mm. Full print stitched using 5× magnification (A) and 10× magnification of 4×4 pixel pillars spaced 16 pixels apart (B).

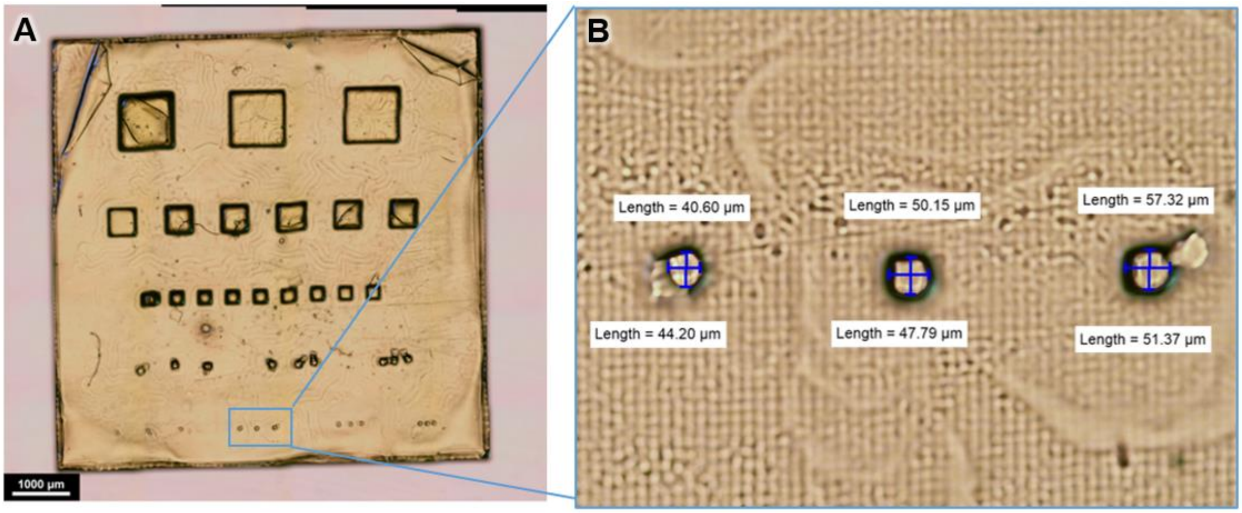

Figure S39. Optical microscope images of the print containing pillars of varying size using photosystem 2 after 365 nm exposure. Images taken using top-lighting. Total print dimensions are 7.5 mm × 7.5 mm. Full print stitched using 5× magnification (A) and 10× magnification of 4×4 pixel pillars spaced 16 pixels apart (B).

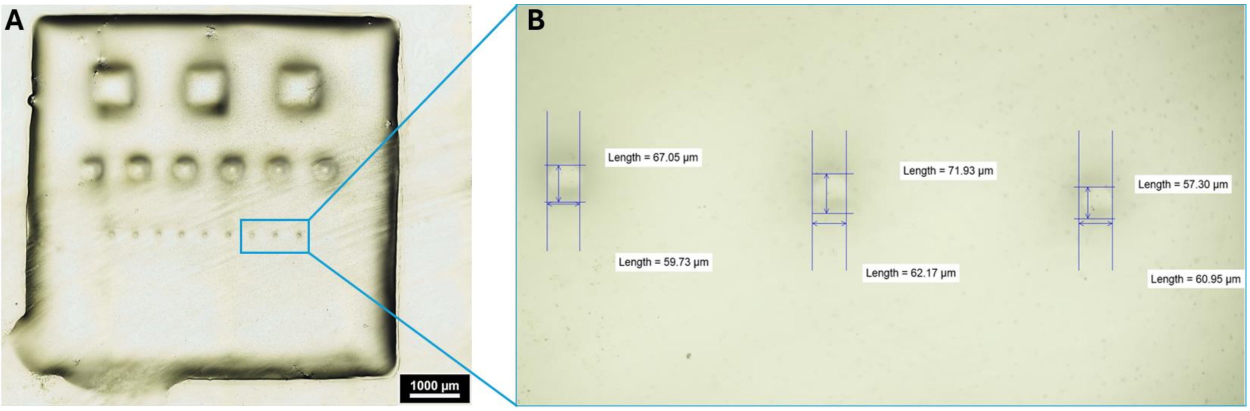

Figure S41. (A) Optical microscope image of the print containing pillars of varying size prepared using photosystem 1 and 405 nm light printing. Image taken using top-lighting. Total print dimensions are 7.5 mm × 7.5 mm. (B) Microscope image of the smallest resolved pillars (16×16 pixels).

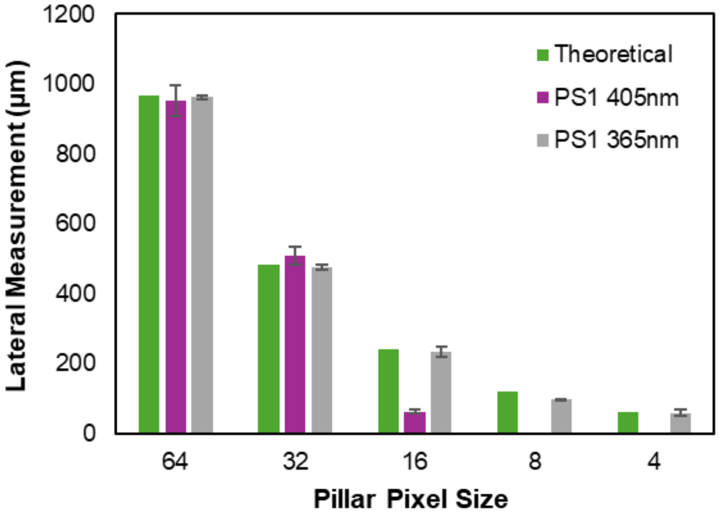

Figure S42. Bar chart displaying the compiled resolution results for measurements of pillar prints with photosystem 1 resin, including theoretical values (green), 405 nm prints (purple), and 365 nm prints (grey). Error bars are plotted as standard deviation calculated from six measurements per pillar of varying size.

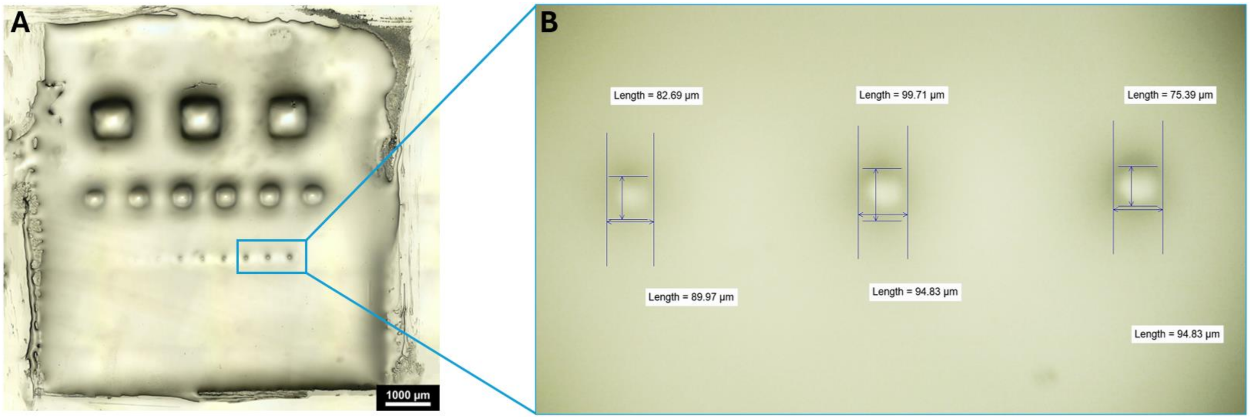

Figure S43. (A) Optical microscope image of the print containing pillars of varying size prepared using photosystem 2 and 460 nm light printing. Image taken using top-lighting. Total print dimensions are 7.5 mm × 7.5 mm. (B) Microscope image of the smallest resolved pillars (16×16 pixels).

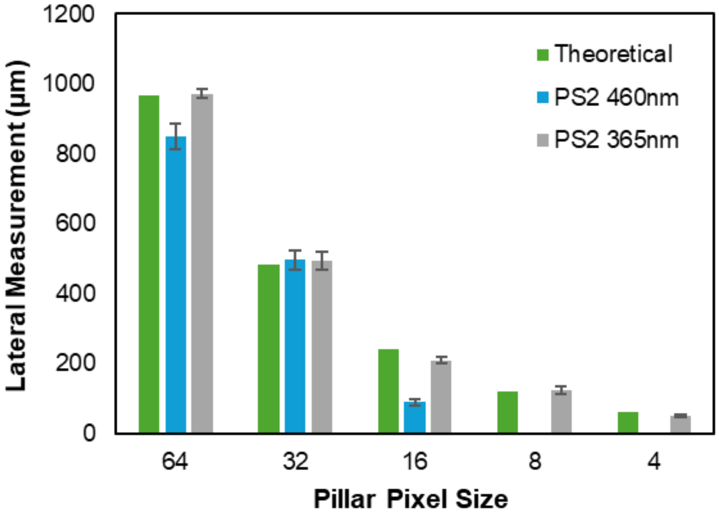

Figure S44. Bar chart displaying the compiled resolution results for measurements of pillar prints with photosystem 2 resin, including theoretical values (green), 460 nm prints (purple), and 365 nm prints (grey). Error bars are plotted as standard deviation calculated from six measurements per pillar of varying size.

S2.14. Supplemental Proof-of-Concept Prints

Additional images and renderings for multimaterial prints are provided below.

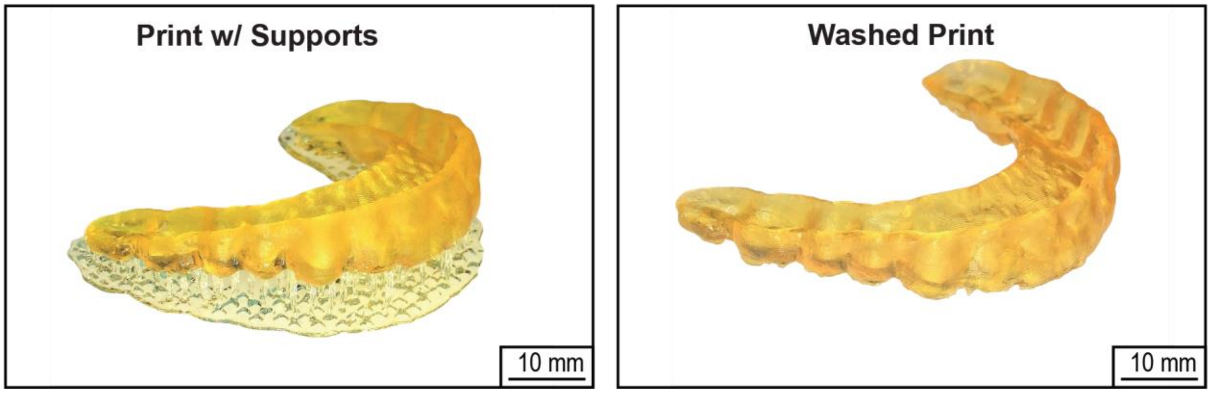

Figure S45. Digital microscope images of a retainer printed with dissolvable supports present (left) and after dissolving away the supports in ethyl acetate (right). Background has been removed for clarity.

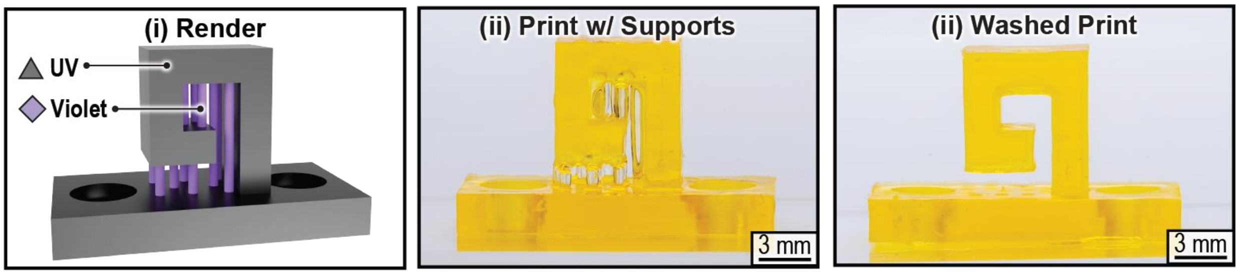

Figure S46. A hook with an overhang, showing (i) color-coded rendering, (ii) image of a print with dissolvable supports present, and (iii) image of a print after dissolving away the supports in ethyl acetate.

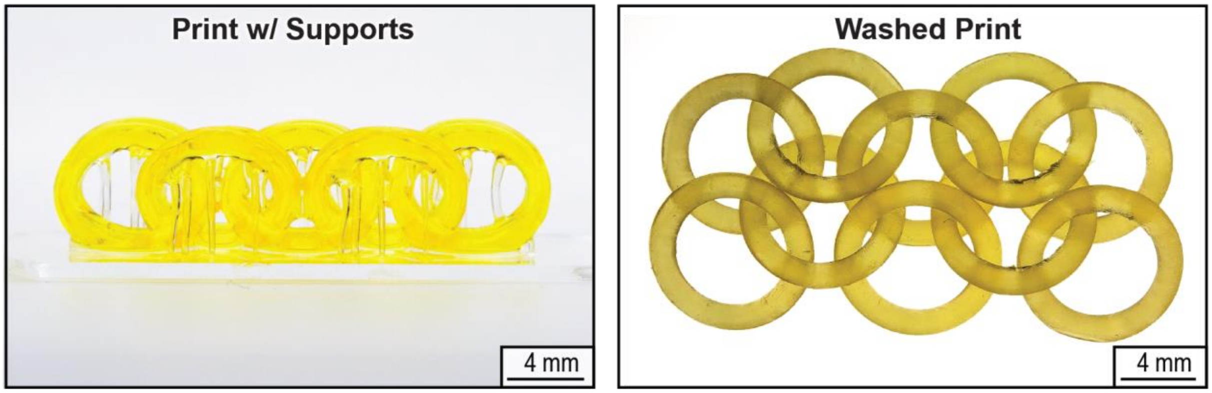

Figure S47. Picture of chainmail with supports still present (left) and digital microscope image of chainmail after dissolvable supports have been washed away in ethyl acetate (right).

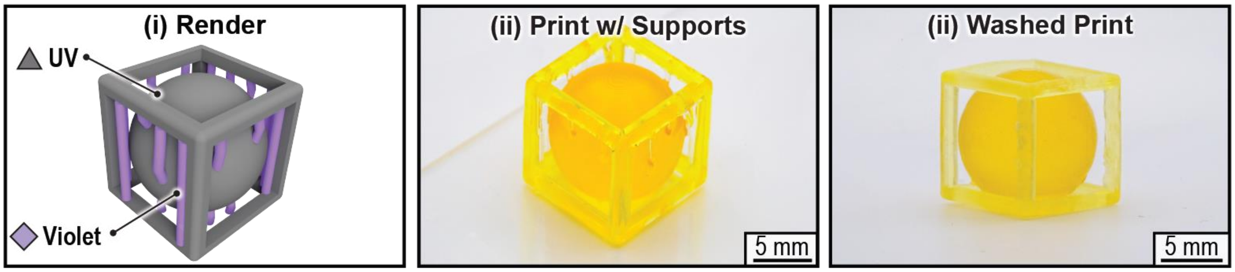

Figure S48. Ball in box[S7], showing (i) color-coded rendering, (ii) image of a print with dissolvable supports present, and (iii) image of a print after dissolving away the supports in ethyl acetate.

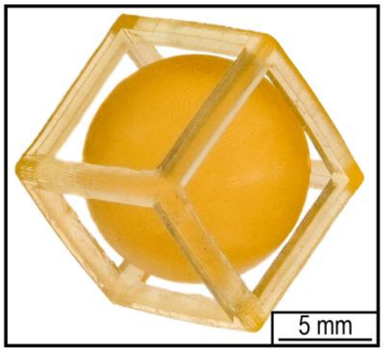

Figure S49. Digital microscope image of a ball in a box after dissolvable supports have been washed away in ethyl acetate. Background has been removed for clarity.

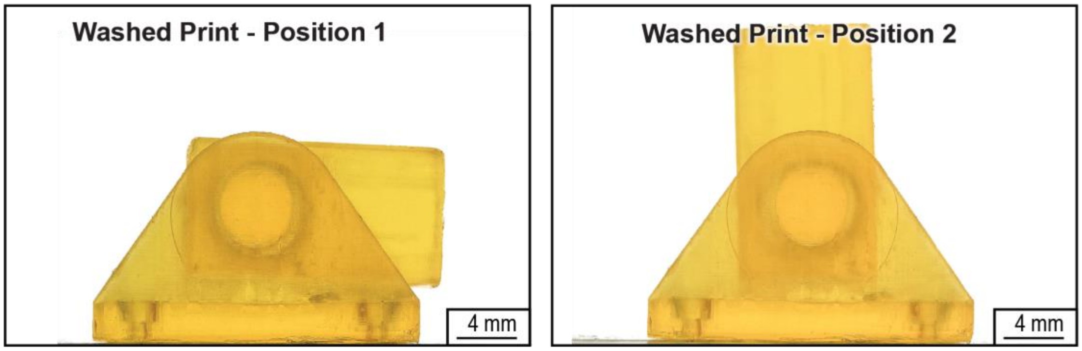

Figure S50. Digital microscope images of a revolute joint print in two positions after dissolvable supports have been washed away in ethyl acetate. Background has been removed for clarity.

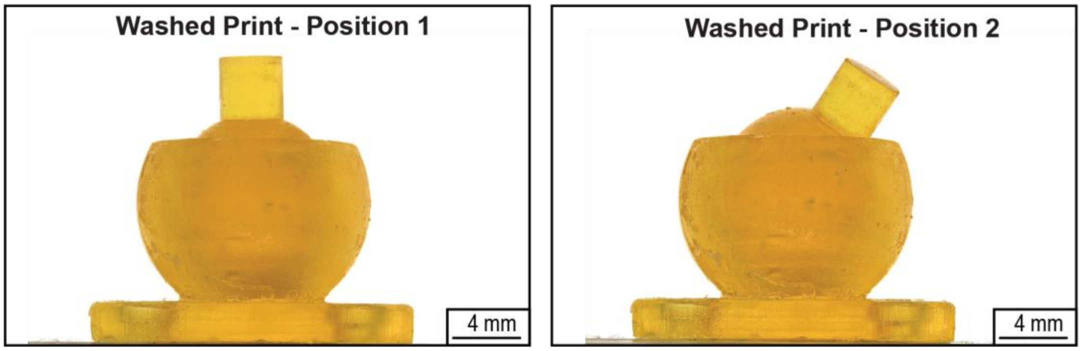

Figure S51. Digital microscope images of a ball and socket joint print in two positions after dissolvable supports have been washed away in ethyl acetate. Background has been removed for clarity.

S2.15. Computed Tomography (CT)

High resolution X-ray computed tomography to quantify 3D resolution. Computed Tomography (CT) was performed on three complex prints: retainer, revolute joint, and ball and socket joint. All prints were washed in ethyl acetate for 20 minutes to remove supports and then vacuum dried for 12 hours prior to CT scanning. 3D ("STL") files were generated from raw CT data for each of the scanned samples. These STL files were then analyzed in CloudCompare, a 3D point cloud and triangular mesh processing software.

Overlaying STL files for 3D resolution analysis. Both the original print files and the reconstructed STL files were loaded into the CloudCompare software. A point cloud alignment was performed by manually translating the original print file to overlap with the CT models and then performing an automated fine registration 20 iterations of finely aligning the point clouds. The "PoissonRecon" plugin was then used to reconstruct a triangle-based mesh by solving a 3D Laplacian system with positional value constraints. Cloud to mesh unsigned distances were computed with the original print STL file as the referenced file and the CT data model as the compared. A color scale was generated and kept consistent throughout each analysis where blue identifies areas that are the most accurate characterized by the smallest distances between the two point clouds, while red identifies areas that have the furthest distances (max = 1.0 mm). Slices were segmented from each print file using a slicing tool. Animations were performed by selecting viewpoints and interpolating positionally between them.

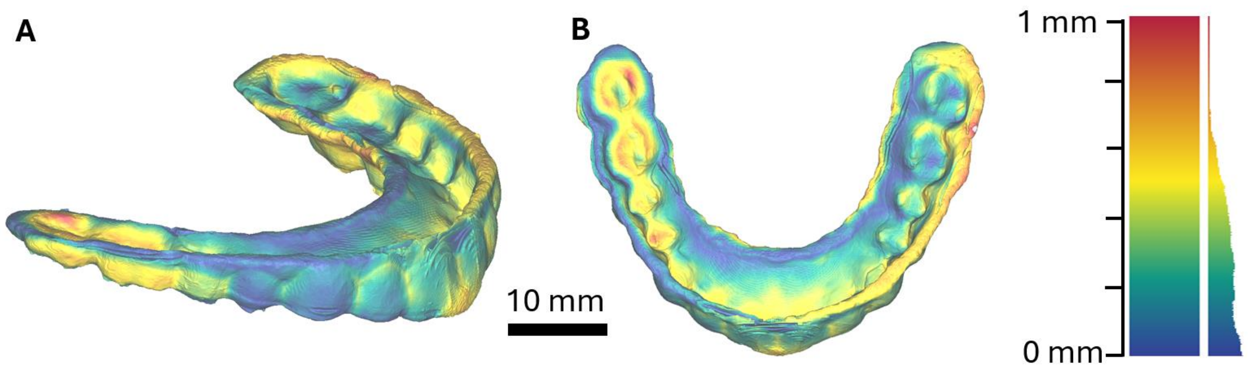

Figure S52. CT data for retainer print. Corner view (A) and top view (B) with corresponding histogram of point cloud distance distribution. Scan parameters: Zeiss. Flat panel, 70kV, 8.5W, 0.05s acquisition time, 5 samples per view, detector 140 mm, source -65.3 mm, camera bin 1, angles 180+fan, 3001 views, default HART, LE3 filter, dithering, sample drift correction. Single reference. Reconstructed with center shift -0.307, beam hardening 0.3, theta 0, byte scaling [-0.003, 0.04], binning 1, recon filter smooth (kernel size = 0.7). Total slices = 1456.

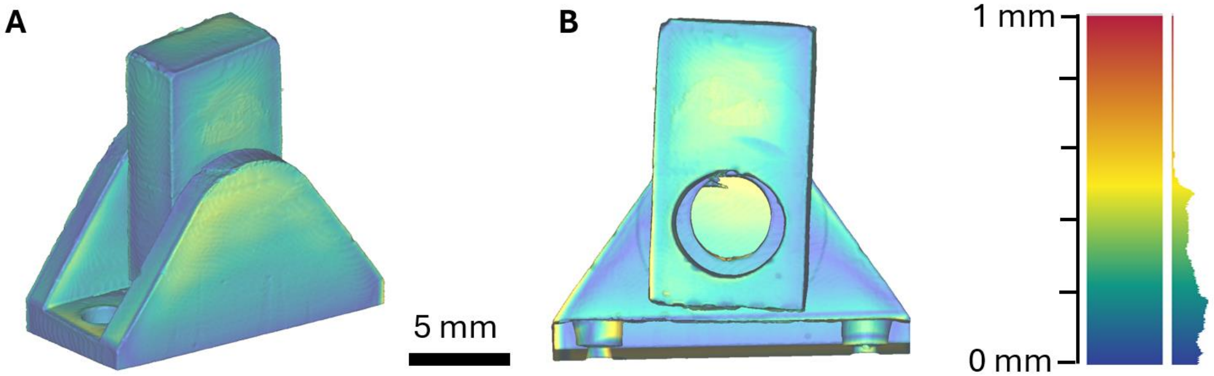

Figure S53. CT data for revolute joint print. Corner view (A) and cross-section view (B) with corresponding histogram of point cloud distance distribution. Scan parameters: Zeiss. Flat panel, 70kV, 8.5W, 0.06s acquisition time, 5 samples per view, detector 190.5 mm, source -47.3 mm, camera bin 1, angles ±180, 4501 views, air filter, dithering, sample drift correction. Single reference. Reconstructed with center shift -0.099, beam hardening 0.05, theta 0, byte scaling [-0.008, 0.5], binning 1, recon filter smooth (kernel size = 0.7). Total slices = 1298.

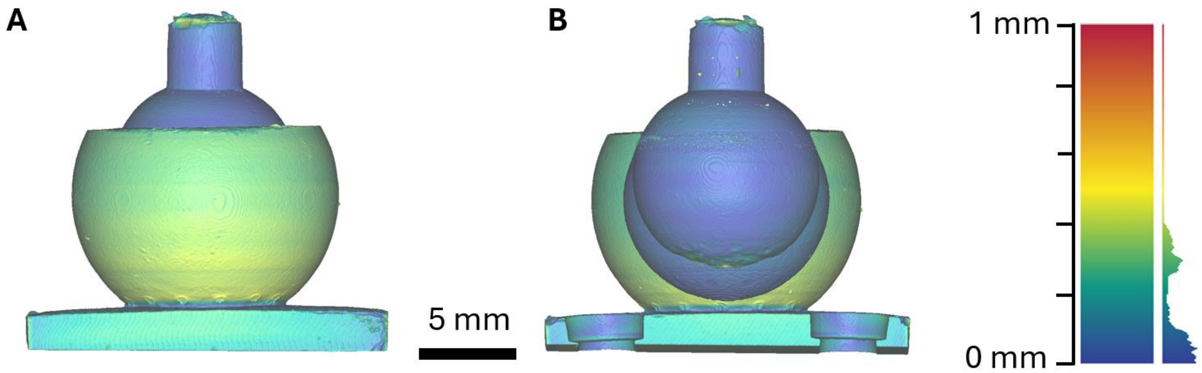

Figure S54. CT data for ball and socket joint print. Corner view (A) and cross-section view (B) with corresponding histogram of point cloud distance distribution. Scan parameters: Zeiss. Flat panel, 70kV, 8.5W, 0.06s acquisition time, 5 samples per view, detector 190.5 mm, source -47.3 mm, camera bin 1, angles ±180, 4501 views, air filter, dithering, sample drift correction. Single reference. Reconstructed with center shift -0.099, beam hardening 0.05, theta 0, byte scaling [-0.008, 0.5], binning 1, recon filter smooth (kernel size = 0.7). Total slices = 1298.

S2.16. Cure Depth Experiments

The dual wavelength printer with LRS20 lens configurations was calibrated to focus the projection pattern onto the bottom of a petri dish (Fisher Brand 35 mm × 10 mm, cat.no. FB0875711YZ). For each resin, the petri dish was filled, placed onto the build area of the printer and a single projection was displayed (**Figure S55**) at 50 mW/cm^2 for 365 nm, 405 nm, or 460 nm for various amounts of time depending on the resin system. Next, left over resin was removed and the print was washed three times with isopropanol before measuring thickness using a depth gauge (Mitutoyo 543-783B-10 Absolute Digimatic Indicator). Jacobs working curves were generated for both photosystem 1 and photosystem 2 (**Figures S56-S57**).

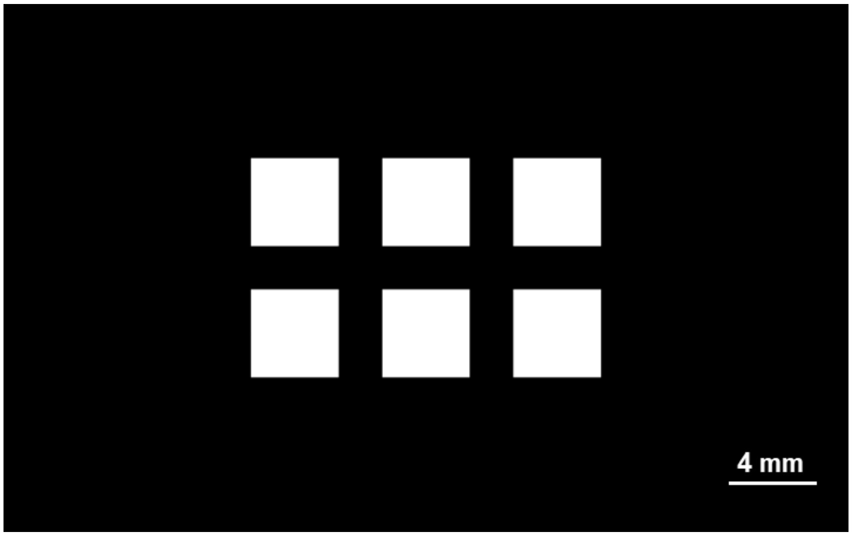

Figure S55. Projection image for cure depth experiments. Each square is 4mm × 4mm.

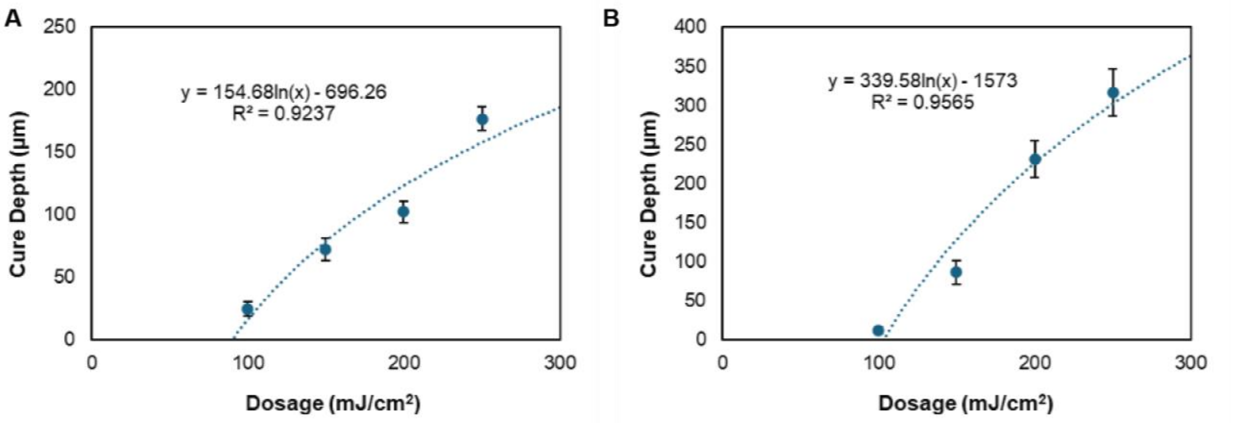

Figure S56. Working curves for PS1 resin irradiated with (A) 365nm and (B) 405nm light. Dashed lines represent fits to the data points, which were averages (n = 4) with error bars representing ± 1 standard deviation from the mean.

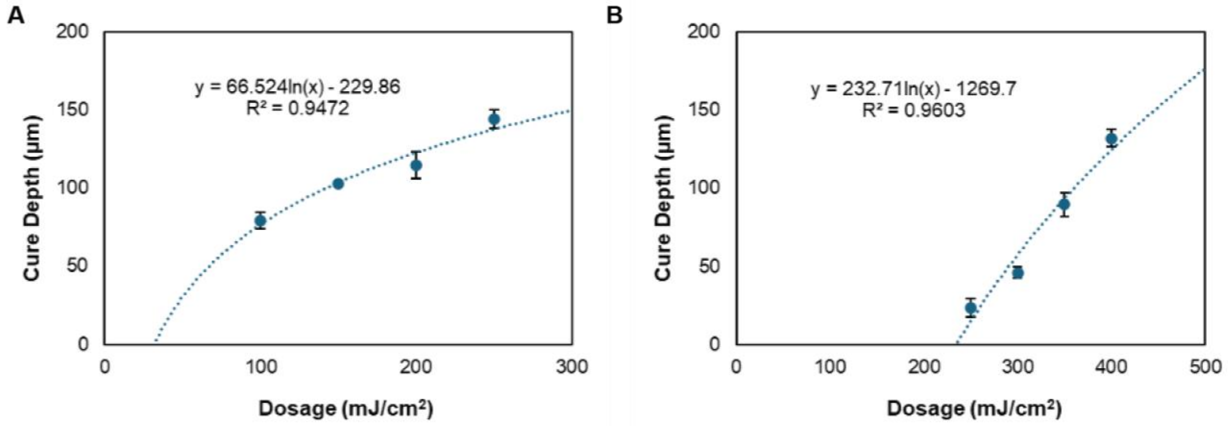

Figure S57. Working curves for PS2 resin irradiated with (A) 365nm and (B) 405nm light. Dashed lines represent fits to the data points, which were averages (*n* = 4) with error bars representing ± 1 standard deviation from the mean.

S2.17. Real-Time UV-Vis Absorption Spectroscopy

To track the change in absorbance during photocuring with different wavelengths of light, we performed in-situ UV-vis absorption spectroscopy using a custom transmission setup (**Figure S2**). Using LabView software, our lab developed a code that can simultaneously control both the LED "on/off" intervals and OceanView spectrophotometer software. Glass slide cells with 50 µm spacers were filled with monomers only (no photosystem) and used to collect the backgrounded spectrum. Subsequently resin containing the desired photosystem components were loaded in a cell and irradiated. The "on" time for the LED was set to 1000 ms at an intensity of 50 mW/cm^2, and the "off" time was set to 350. Spectra were collected during the "off" cycle using an integration time of 100 ms, boxcar set to 5, and scans-to-average set to 3. The cycle repeated a total of 100 times, providing a total exposure time of 100 seconds. The resulting raw data was baseline corrected and plotted below. These results begin to inform our understanding of how coloration occurs during the curing process, which can be used to develop systems with reduced color in the future.

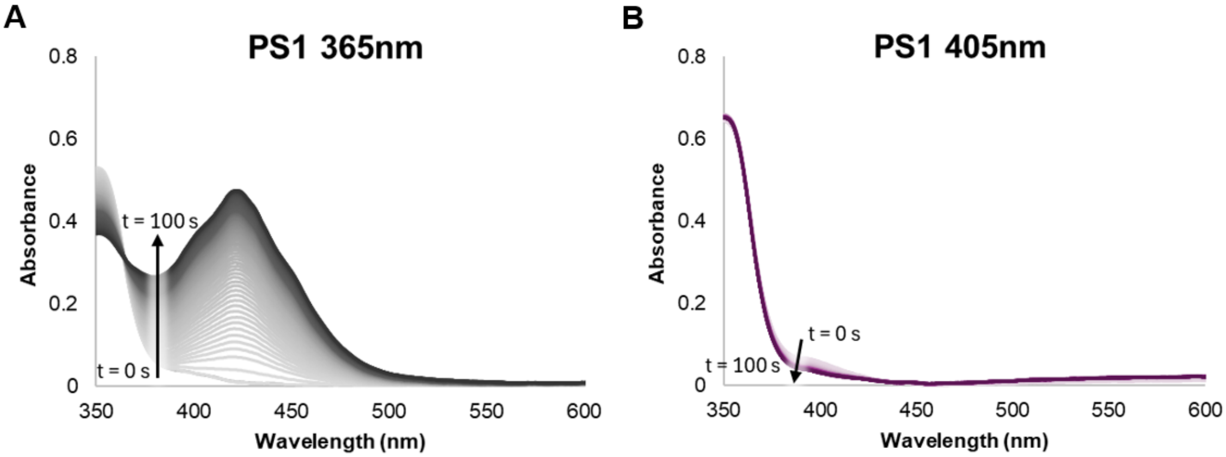

Figure S58. Real-time UV-vis absorption spectra for photosystem 1 irradiated with (a) 365 nm and (b) 405 nm, both at an intensity of 50mW/cm^2. The total LED "on" time (i.e., sample exposure time) was 100 seconds, where start to finish is represented by light to dark lines, respectively.

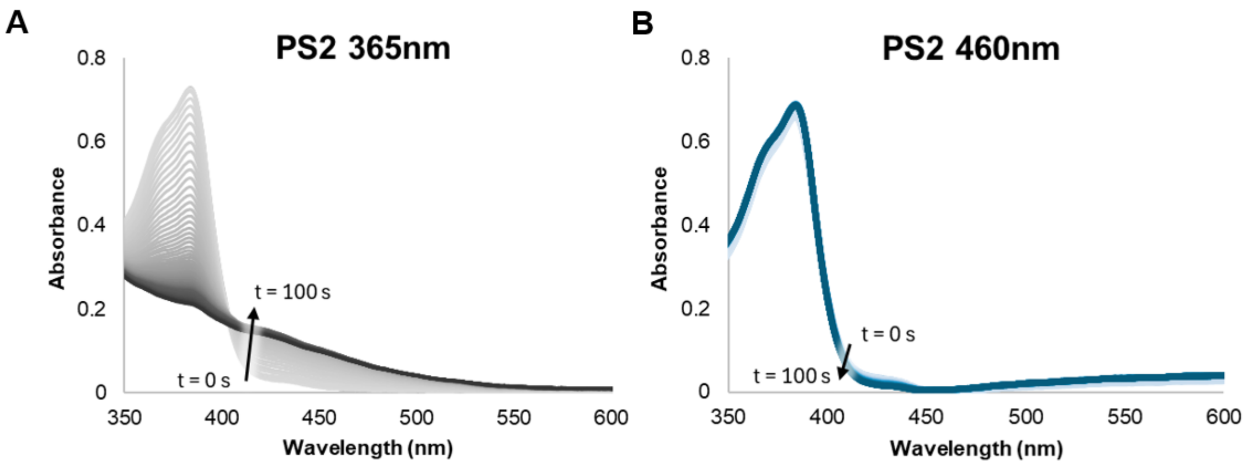

Figure S59. Real-time UV-vis absorption spectra for photosystem 2 irradiated with (a) 365 nm and (b) 460 nm, both at an intensity of 50mW/cm^2. The total LED "on" time (i.e., sample exposure time) was 100 seconds, where start to finish is represented by light to dark lines, respectively.

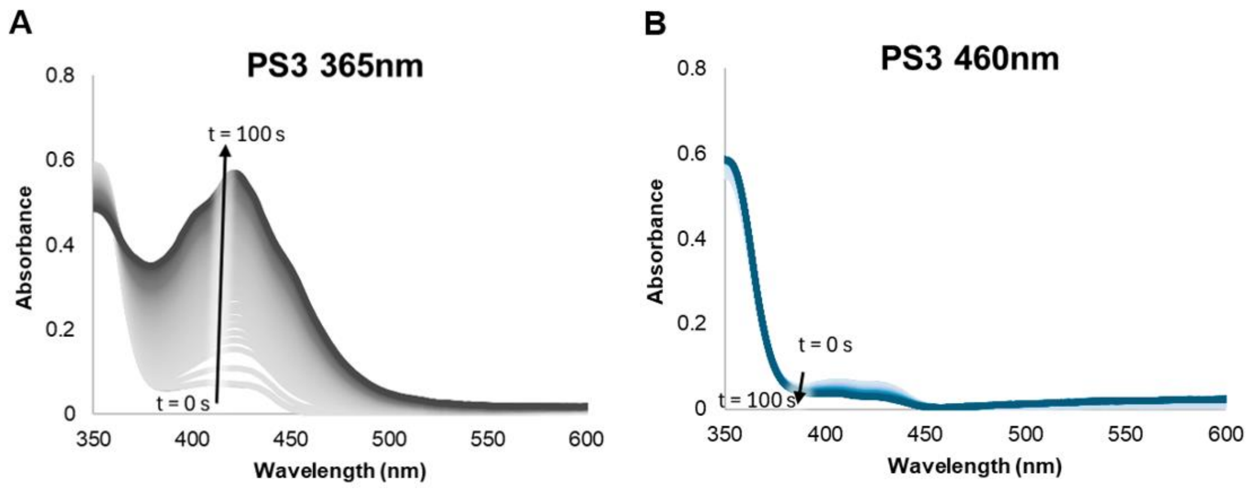

Figure S60. Real-time UV-vis absorption spectra for photosystem 3 irradiated with (a) 365 nm and (b) 460 nm, both at an intensity of 50mW/cm^2. The total LED "on" time (i.e., sample exposure time) was 100 seconds, where start to finish is represented by light to dark lines, respectively.

S2.18. Nuclear Magnetic Resonance (NMR) Spectra

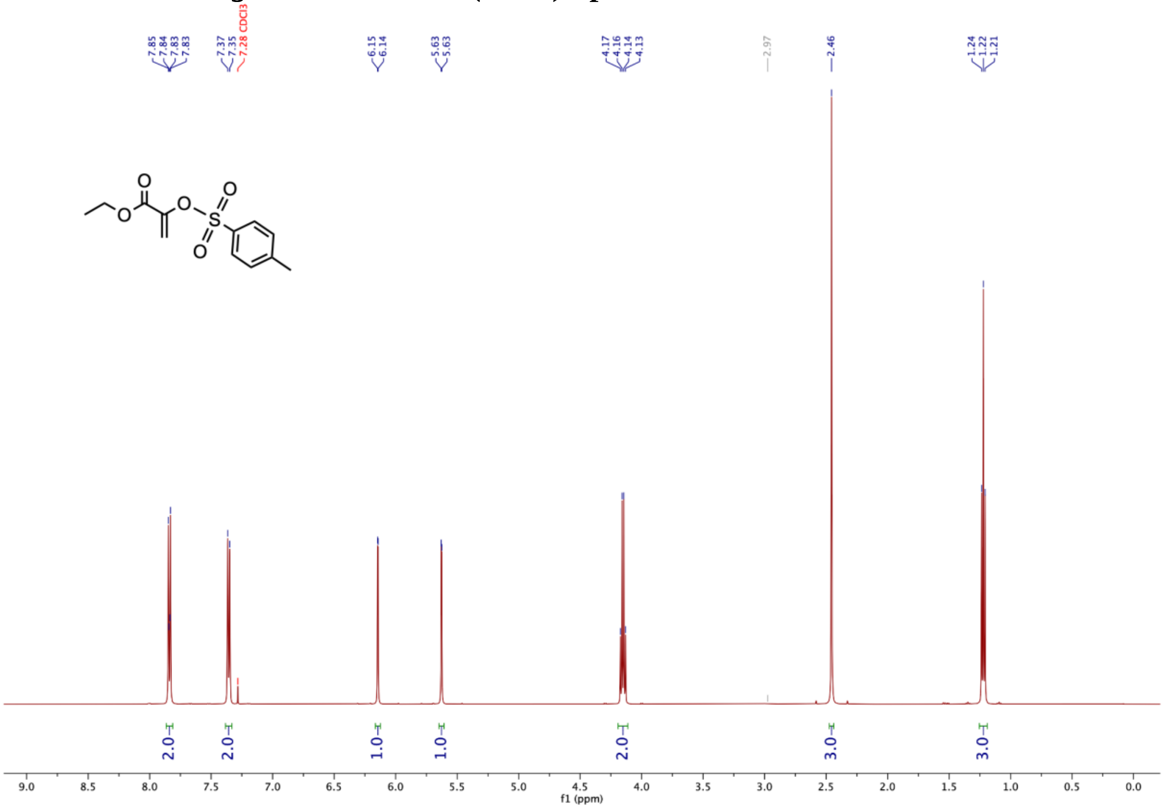

Figure S61. ^{1}H NMR spectrum for ethyl 2-(tosyloxy)acrylate (EVS).

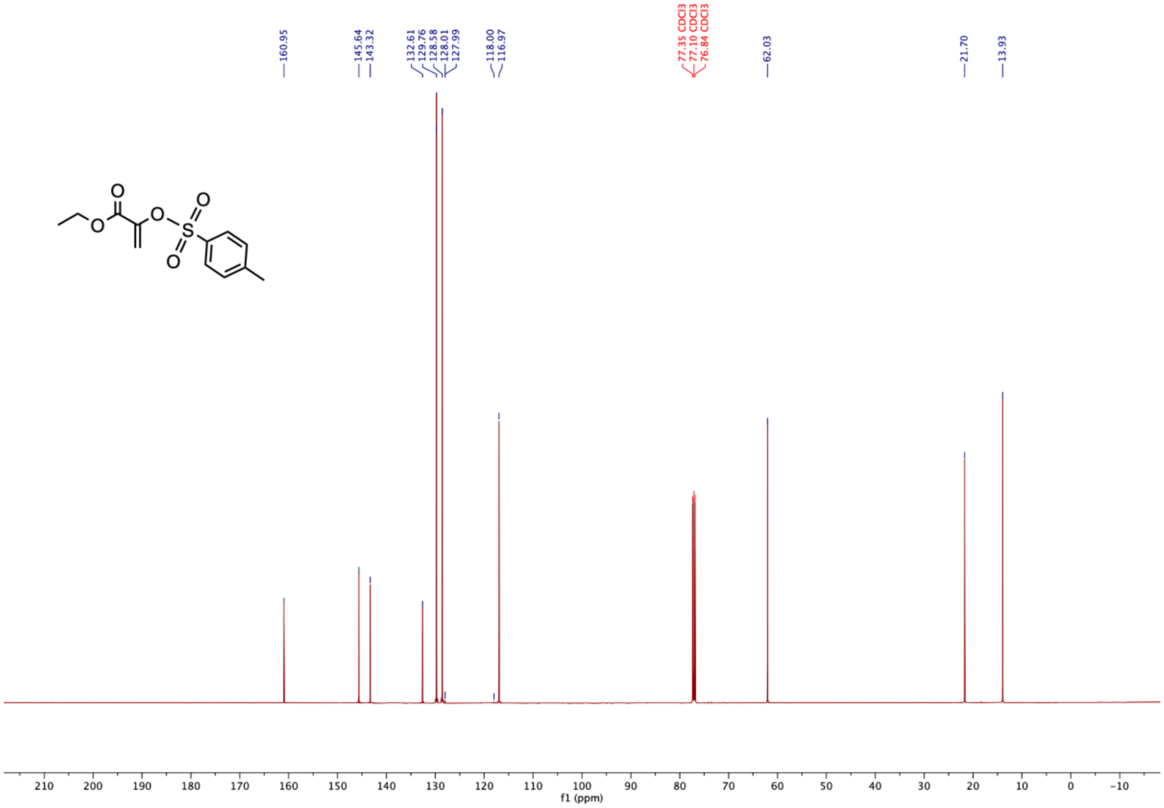

Figure S62. ^{13}C NMR spectrum for ethyl 2-(tosyloxy)acrylate (EVS).

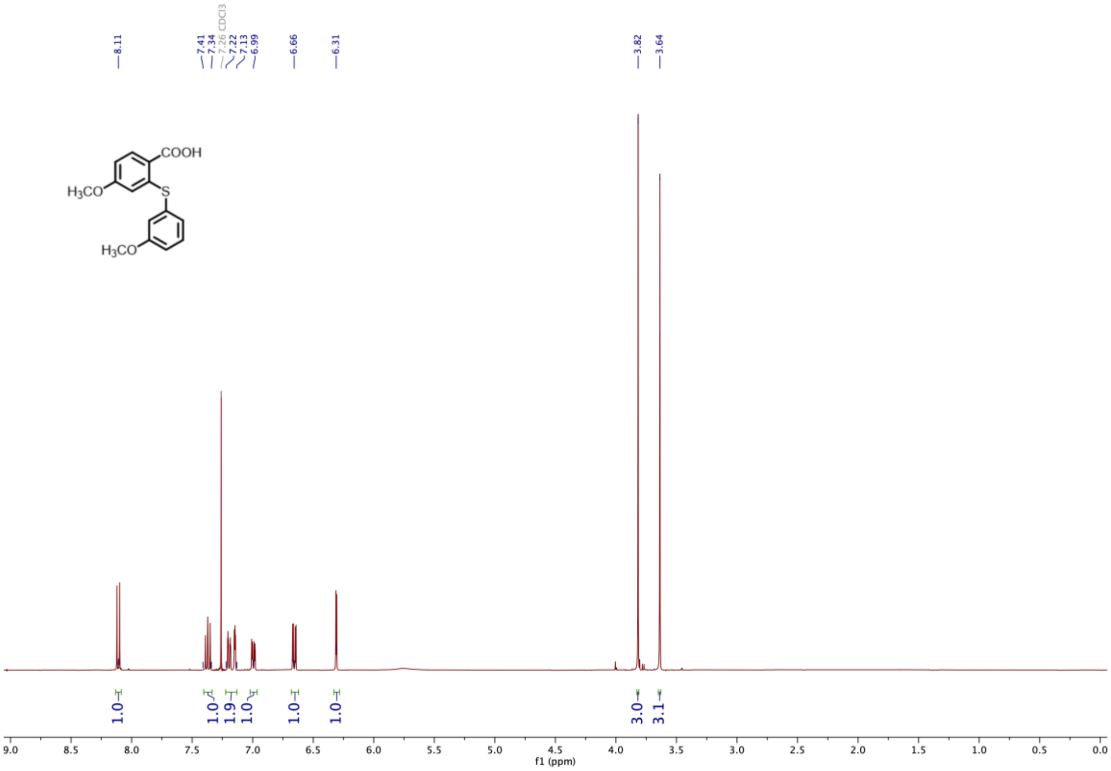

Figure S63. ^{1}H NMR spectrum for 4-methoxy-2-((3-methoxyphenyl)thio)benzoic acid.

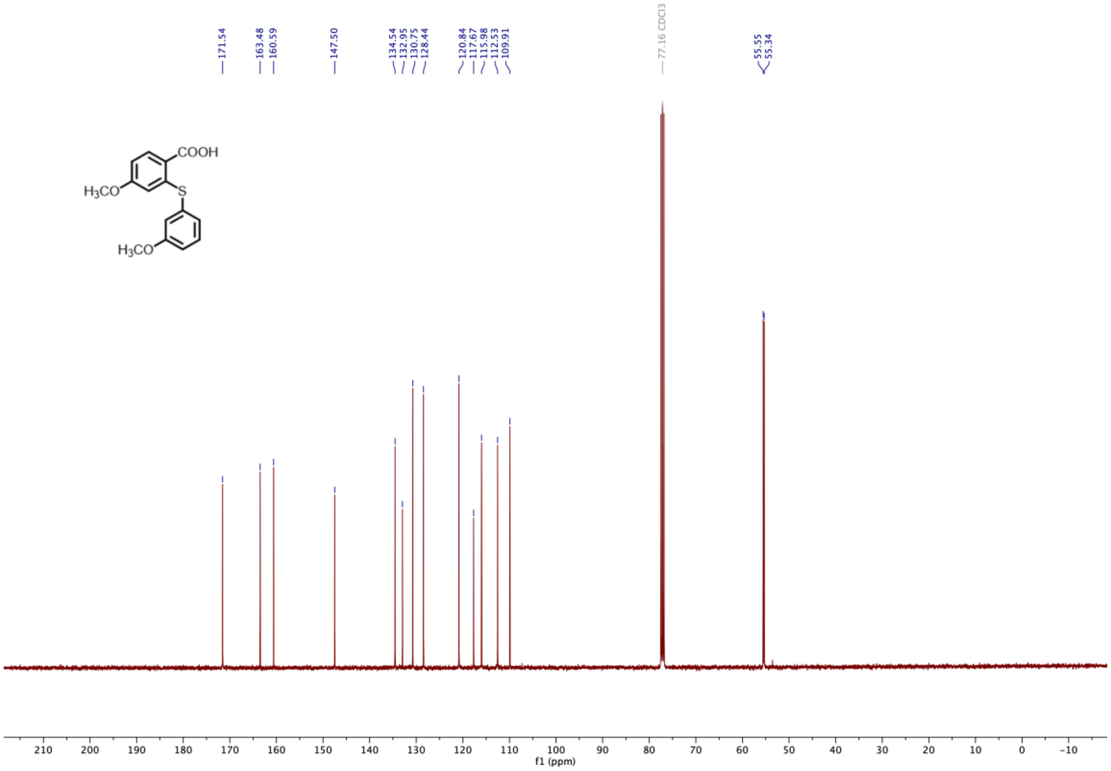

Figure S64. ^{13}C NMR spectrum for 4-methoxy-2-((3-methoxyphenyl)thio)benzoic acid.

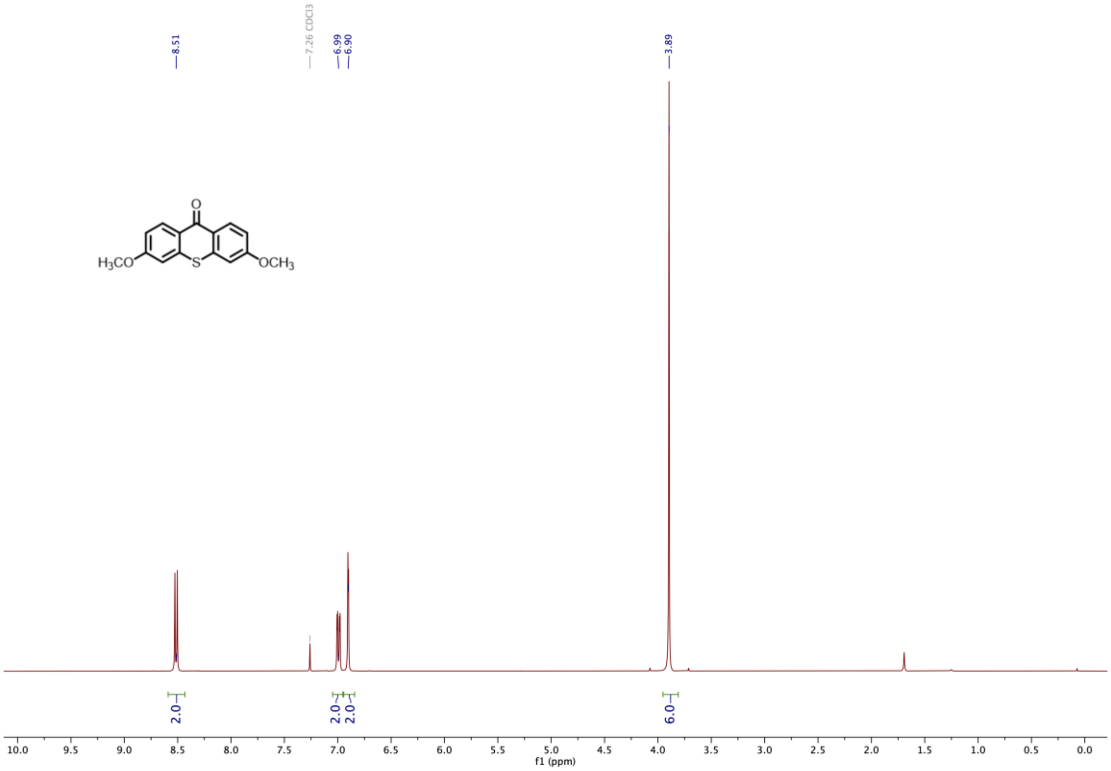

Figure S65. ^{1}H NMR spectrum for 3,6-dimethoxy-9H-thioxanthen-9-one (MeOTX).

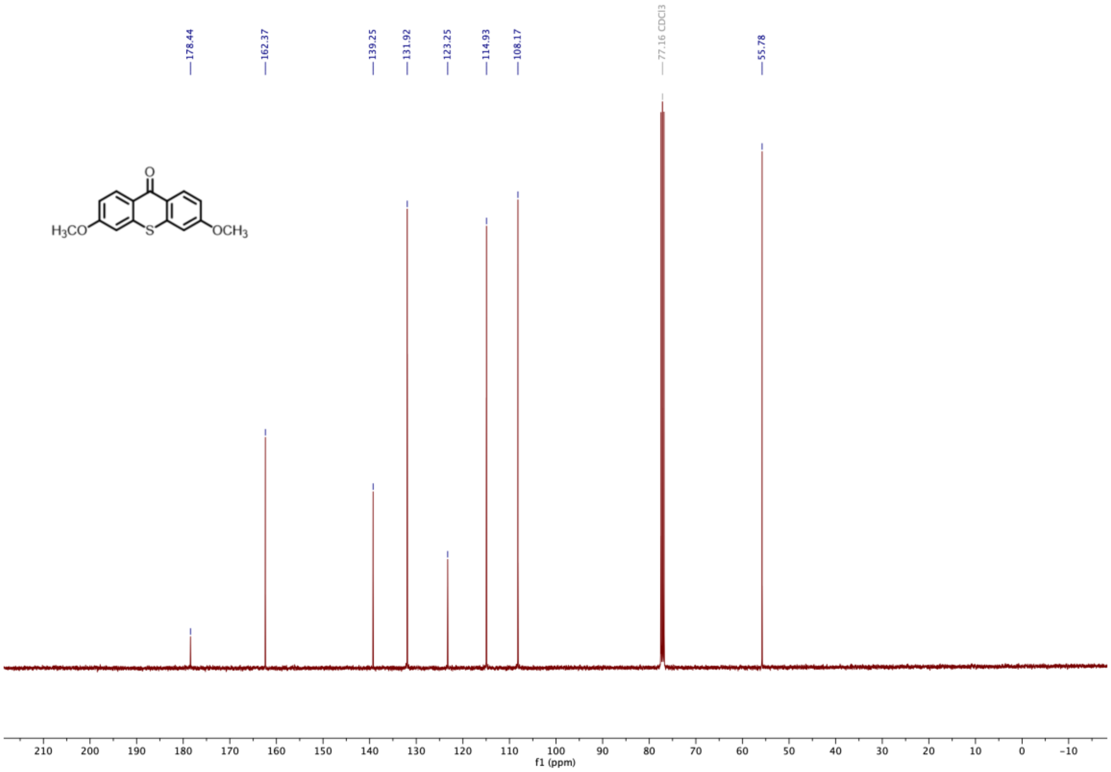

Figure S66. ^{13}C NMR spectrum for 3,6-dimethoxy-9H-thioxanthen-9-one (MeOTX).

S3. MOVIE CAPTIONS

All movies were recorded on a digital camera (Sony a7s II with Sony FE 2.9/90 Macro G OSS lens). Scale bars have been overlaid onto each movie and playback speed has been noted when altered from real time.

Movie S1: Representative example for support dissolution using a reference disk print containing 750 µm-wide supports placed in ethyl acetate for five minutes to facilitate their dissolution. The print was produced using photosystem 1 with a 365 nm / 405 nm light projection, in line with **Fig. 3A**. The washing step is played at 10× speed.

Movie S2: Rotational view of retainer obtained from computed tomography scanning. Details of the 3D structures can be found in **Fig. 4A** of the main manuscript and **Fig. S45**.

Movie S3: Movement of chainmail with gravity in real time. Details of the 3D structures can be found in Fig. 4C of the main manuscript and **Fig. S40**.

Movie S4: Movement of washed ball in a box in real time. Details of the 3D structures can be found in **Figs. S41-42**.

Movie S5: Movement of washed revolute joint in real time. Details of the 3D structures can be found in **Fig. 4D** of the main manuscript and **Fig. S43**.

Movie S6: Rotation and cross section view of revolute joint obtained from computed tomography scanning. Details of the 3D structures can be found in **Fig. 4D** of the main manuscript and **Fig. S46**.

Movie S7: Movement of washed ball & socket joint in real time. Details of the 3D structures can be found in **Fig. 4E** of the main manuscript and **Fig. S44**.

Movie S8: Rotation and cross section view of ball & socket joint obtained from computed tomography scanning. Details of the 3D structures can be found in **Fig. 4D** of the main manuscript and **Fig. S47**.

S4. REFERENCES

(S1) Page, Z.; Kim, J.-W.; Allen, M.; Cater, H.; Uddin, A.; Recker, E.; Freeman, B. Hybrid Epoxy-Acrylate Resins for Wavelength-Selective Multimaterial 3D Printing. May 2, 2024. https://doi.org/10.21203/rs.3.rs-4237033/v1.

(S2) Seidler, K.; Griesser, M.; Kury, M.; Harikrishna, R.; Dorfinger, P.; Koch, T.; Svirkova, A.; Marchetti-Deschmann, M.; Stampfl, J.; Moszner, N.; Gorsche, C.; Liska, R. Vinyl Sulfonate Esters: Efficient Chain Transfer Agents for the 3D Printing of Tough Photopolymers without Retardation. *Angew Chem Int Ed* **2018**, *57* (29), 9165–9169. https://doi.org/10.1002/anie.201803747.

(S3) Cazin, I.; Plevová, K.; Alabiso, W.; Vidović, E.; Schlögl, S. Dual-Wavelength Vat Photopolymerization 3D Printing with Hybrid Acrylate-Epoxy Resins: Influence of Resin Composition on Microstructure and Mechanical Properties. *Adv Eng Mater* **2024**, *26* (8), 2301699. https://doi.org/10.1002/adem.202301699.

(S4) *Using Formlabs Finishing Tools*. formlabs. https://support.formlabs.com/s/article/finishing-tools?language=en_US.

(S5) *Advanced support removal techniques*. formlabs. https://support.formlabs.com/s/article/Advanced-Support-Removal-Techniques?language=en_US.

(S6) ctrlV. *Test your 3D printer! v2*. https://www.thingiverse.com/thing:1019228.

(S7) Parmsco. *Ball in a Box*. https://www.thingiverse.com/thing:72788/files#google_vignette.
