# Supplementary material for: Multicolor Digital Light Processing 3D Printing Enables Dissolvable Supports for Freestanding and Non-Assembly Structures
Source: ACS Cent Sci. 2025 May 29;11(6):975–82. doi: 10.1021/acscentsci.5c00289 (PMC12203260; doi:10.1021/acscentsci.5c00289)
Supplement: Supplementary file 10 [file oc5c00289_si_010.pdf]

Name: Peer Review Information for "Multicolor Digital Light Processing 3D Printing Enables Dissolvable Supports for Freestanding and Non-Assembly Structures"

First Round of Reviewer Comments

Reviewer: 1

Comments to the Author

This manuscript presents a novel approach to multimaterial digital light processing (DLP) 3D printing using wavelength-selective resins to create dissolvable supports with very high printing quality. The work is well-executed and addresses an important challenge in additive manufacturing. The authors demonstrate the potential of their method through several proof-of-concept structures. Overall, it is a highly complete manuscript with very supportive results. I recommend this manuscript for publication in ACS Central Science with a suitable revision. Here are some detailed comments for consideration:

1.The curing depth under different wavelengths should be characterized, and the printing fidelity should be evaluated for both wavelengths.

2.How is the interfacial bonding between the two different parts? This should be discussed or characterized.

3.For all the pictures of as-printed samples (Figure 3F-iv; Figure S39-ii; Figure S40), it appears that the supported parts are over-cured only at the interfacial region. However, the washed samples appear very smooth. This discrepancy should be explained.

4.The authors mention that HEA is used to accelerate the cationic curing. This claim should be supported with experimental results.

5.It seems that the soluble thermoplastic part is transparent, while the crosslinked part has a yellow color. Why is that?

6.The captions for Figure S36 and S37 are the same. Please confirm if S37 corresponds to photosystem 2?

Reviewer: 2

Comments to the Author

In this manuscript, Page group developed a novel strategy to incorporate dissolvable supports in DLP printing. Here, they used a multi-color (or dual wavelength) DLP printer. By widely designing the chemistry and photoinitiators, they were able to use one wavelength (365nm) to cure the resin into a stiff thermoset, and use another wavelength (either 405nm or 460nm) to turn the resin into a relatively soft thermoplastics, which can be dissolve quickly at room temperature by ethyl acetate. With this capability, they presented some very interesting demonstrations, such as interlocked chains and ball and socket joint. Overall, this is an excellent work representing a breakthrough in DLP 3D printing. I only have one small concern. The printed thermoset after the solvent treatment (for removing the supports) and drying (100C for 24 hours) showed a significant drop in modulus (from 1.6GPa to 1.2GPa for photosystem 1). The authors attributed this to microcracks from swelling or microvoids during drying. However, this seems to be an arbitrary guess. Can the authors do an SEM to see if there are microcracks or microvoids? Also, can the authors measure the Tg and see if there is a Tg shift?

Author's Response to Peer Review Comments:

Reviewer: 1

Recommendation: Publish in ACS Central Science after minor revisions noted.
Comments: This manuscript presents a novel approach to multimaterial digital light processing (DLP) 3D printing using wavelength-selective resins to create dissolvable supports with very high printing quality. The work is well-executed and addresses an important challenge in additive manufacturing. The authors demonstrate the potential of their method through several proof-ofconcept structures. Overall, it is a highly complete manuscript with very supportive results. I recommend this manuscript for publication in ACS Central Science with a suitable revision.

We thank the Reviewer for their time in carefully reading the manuscript and for recommending publication in *ACS Central Science* after minor revisions. Below we address point-by-point the individual comments/questions raised by Reviewer 1.

1. The curing depth under different wavelengths should be characterized, and the printing fidelity should be evaluated for both wavelengths.

We appreciate this suggestion and in response we have conducted cure-depth experiments and resolution prints for both wavelengths of light. The updated results have been included in section S2.16 (and below for convenience). The print fidelity was further characterized with pillar resolution prints for dissolvable features (405 nm and 460 nm) and included with section S2.13 (and below for convenience). These revealed minimum feature sizes of ~60-100 µm were able to be resolved for dissolvable features. For all print conditions, the print feature fidelity was summarized in Fig. S42 and S44). For practical application as support features, we found that ~600 µm was the minimum size that would print for use as vertical bar supports in disk support prints.

New results on pages S39-S40 in the revised supporting information file:

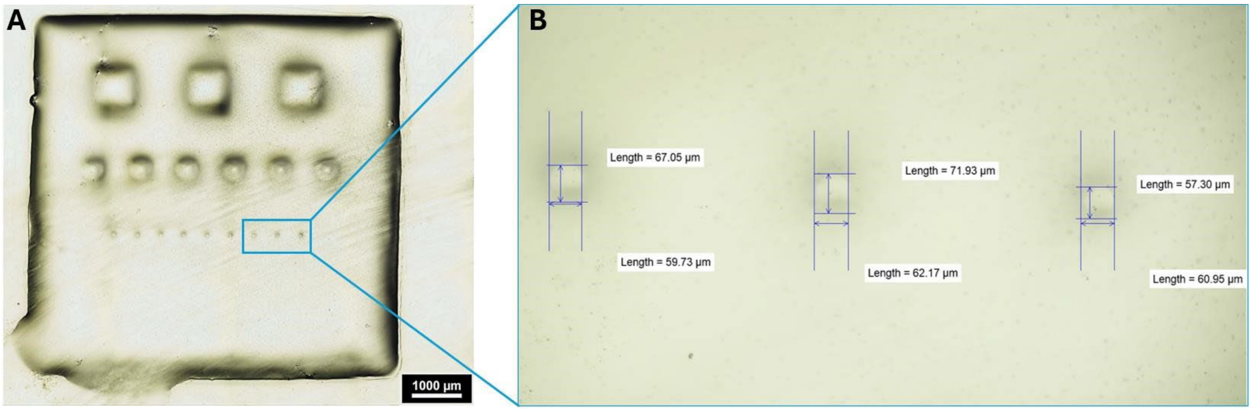

Figure S41. (A) Optical microscope image of the print containing pillars of varying size prepared using photosystem 1 and 405 nm light printing. Image taken using top-lighting. Total print dimensions are 7.5 mm × 7.5 mm. (B) Microscope image of the smallest resolved pillars (16×16 pixels).

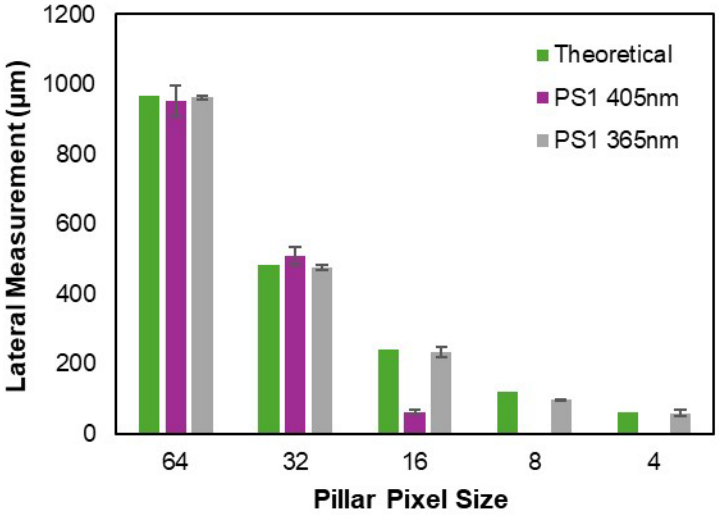

Figure S42. Bar chart displaying the compiled resolution results for measurements of pillar prints with photosystem 1 resin, including theoretical values (green), 405 nm prints (purple), and 365 nm prints (grey). Error bars are plotted as standard deviation calculated from six measurements per pillar of varying size.

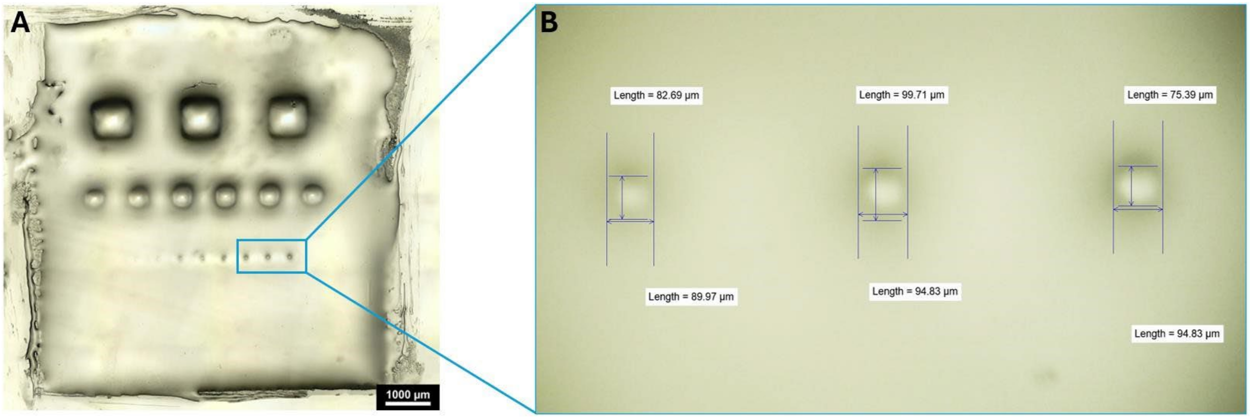

Figure S43. (A) Optical microscope image of the print containing pillars of varying size prepared using photosystem 2 and 460 nm light printing. Image taken using top-lighting. Total print dimensions are 7.5 mm × 7.5 mm. (B) Microscope image of the smallest resolved pillars (16×16 pixels).

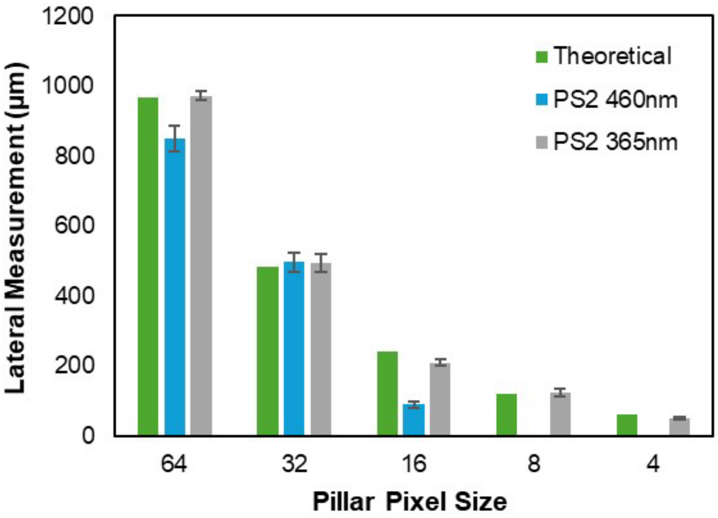

Figure S44. Bar chart displaying the compiled resolution results for measurements of pillar prints with photosystem 2 resin, including theoretical values (green), 460 nm prints (purple), and 365 nm prints (grey). Error bars are plotted as standard deviation calculated from six measurements per pillar of varying size.

New results on pages S44-S45 in the revised supporting information file:

S2.16. Cure Depth Experiments

The dual wavelength printer with LRS20 lens configurations was calibrated to focus the projection pattern onto the bottom of a petri dish (Fisher Brand 35 mm × 10 mm, cat.no. FB0875711YZ). For each resin, the petri dish was filled, placed onto the build area of the printer and a single projection was displayed (**Figure S55**) at 50 mW/cm^2 for 365 nm, 405 nm, or 460 nm for various amounts of time depending on the resin system. Next, left over resin was removed and the print was washed three times with isopropanol before measuring thickness using a depth gauge (Mitutoyo 543-783B10 Absolute Digimatic Indicator). Jacobs working curves were generated for both photosystem 1 and photosystem 2 (**Figures S56-S57**).

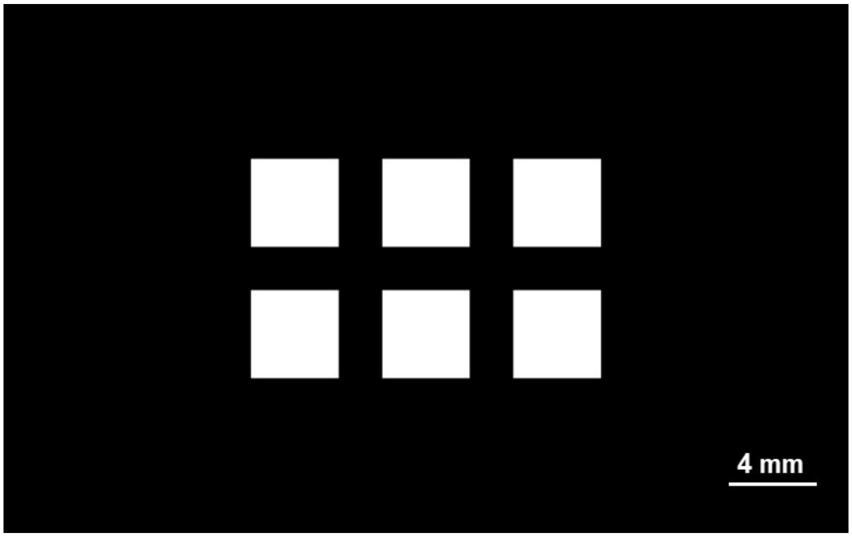

Figure S55. Projection image for cure depth experiments. Each square is 4mm × 4mm.

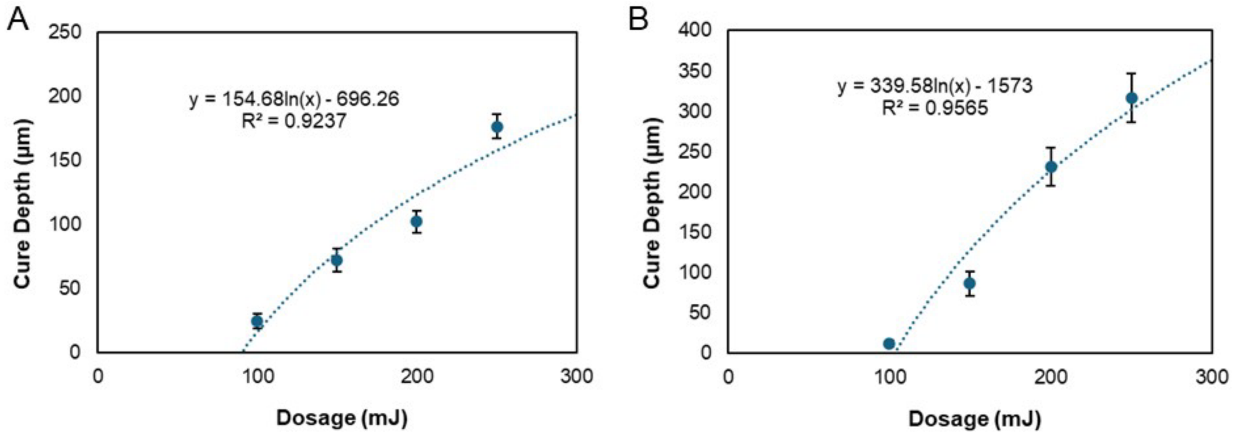

Figure S56. Working curves for PS1 resin irradiated with (A) 365nm and (B) 405nm light. Dashed lines represent fits to the data points, which were averages (n = 4) with error bars representing ± 1 standard deviation from the mean.

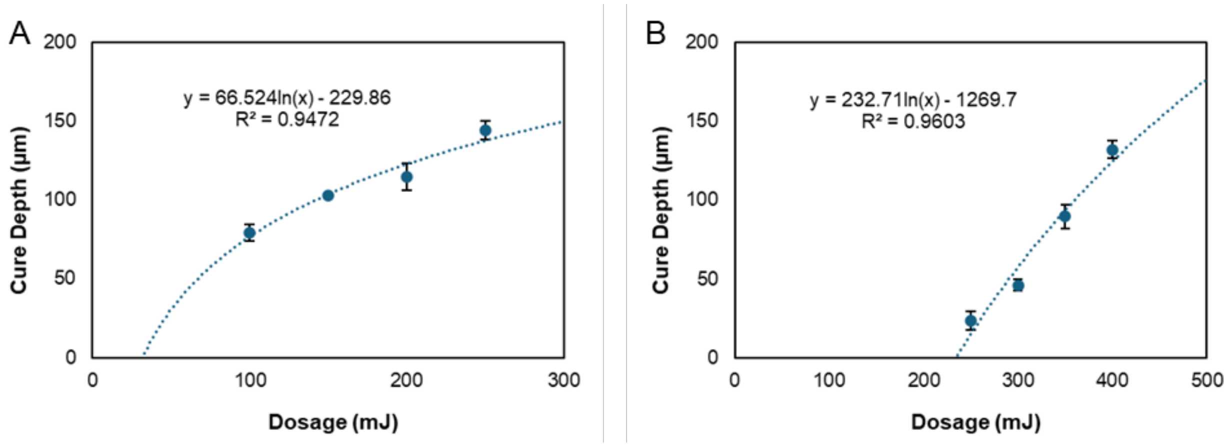

Figure S57. Working curves for PS2 resin irradiated with (A) 365nm and (B) 405nm light. Dashed lines represent fits to the data points, which were averages (n = 4) with error bars representing ± 1 standard deviation from the mean.

2.How is the interfacial bonding between the two different parts? This should be discussed or characterized.

We thank the reviewer for raising this question. We probed interfacial bonding with tensile specimens which were patterned using 365 nm/405 nm light for resin with photosystem 1, and the results are included in section S2.10 (and below for convenience). However, we wish to note that providing quantitative characterization of interfacial mechanical properties (e.g., strength and toughness) for multimaterial 3D printed structures remains an open question that would require extensive characterization with careful controls that goes beyond the scope of the present study. With our experiments we qualitatively found that the interfacial bonding between the two domains was strong by uniaxially tensile testing multimaterial dogbone specimens and observing that fracture occurred away from the dissolvable/non-dissolvable interfaces. We hypothesize that the strength of the interfaces arise from covalent bonding of acrylate chains across the multimaterial domains. Further studies to probe this hypothesis are ongoing.

New/modified text on page 6 of the revised main manuscript file: "Moreover, angled supports (15°, 30°, and 45°) were effective (Fig. S23-24), and interfaces between dissolvable and nondissolvable domains were qualitatively strong as indicated by fracture of multimaterial dogbones under tension away from the interfaces (Fig. S25)."

New results on page S28 of the revised supporting information file:

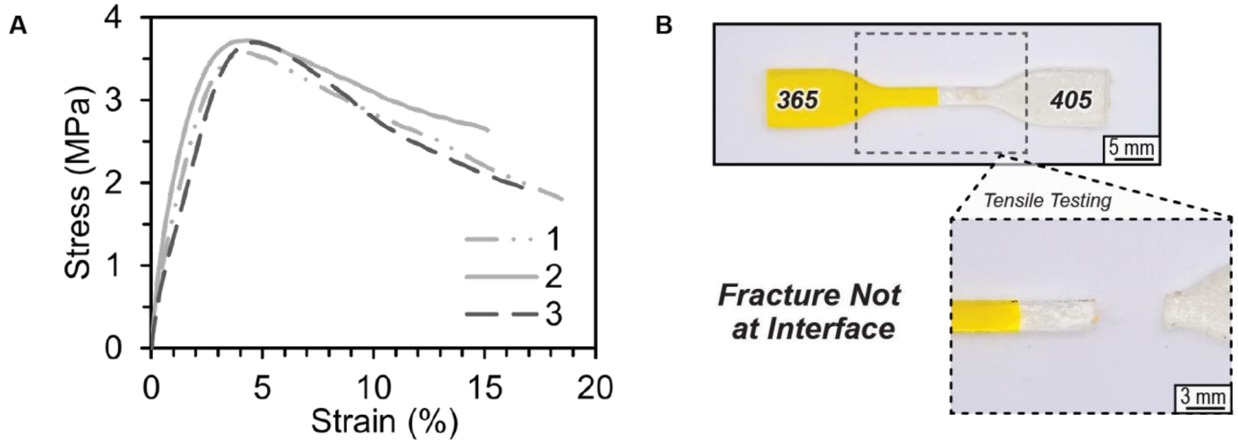

Figure S25. Mechanical testing of multimaterial dogbones prepared using photosystem 1 resin to characterize interfacial bonding between dissolvable (405 nm) and non-dissolvable (365 nm) domains. (A) Stress-strain plot from uniaxial tensile testing. (B) Image of half-and-half dogbone before and after tensile testing showing fracture occurring away from the interface. Dogbone samples were printed with one half exposed to 365 nm light and the other half to 405 nm light (50 mW/cm^2 , 4 s/50 µm layer).

3.For all the pictures of as-printed samples (Figure 3F-iv; Figure S39-ii; Figure S40), it appears that the supported parts are over-cured only at the interfacial region. However, the washed samples appear very smooth. This discrepancy should be explained.

We thank the Reviewer for their query regarding possible interfacial overcuring. We attribute this behavior to the capillary forces between closely packed features during printing, which can hold onto resin along with potentially creating a high local concentration of oligomeric species that can vitrify upon subsequent layer irradiation due to the high optical transparency of the present resin system. Another possibility is that the solvent rinse prior to imaging, which consisted of ~60 second stirring of samples in IPA, may not have been sufficient to remove all unreacted liquid resin especially in small gaps such as those pointed out by Reviewer 1. The fact that dissolution of these 'overcured' regions occurs indicates that these regions are either trapped liquid resin or thermoplastic and hence do not impact the surface finish.

4.The authors mention that HEA is used to accelerate the cationic curing. This claim should be supported with experimental results.

We appreciate the Reviewer for noting this point. We and others have previously demonstrated that the presence of alcohol functionality can accelerate cationic epoxy curing (DOI: 10.21203/rs.3.rs-4237033/v1, 10.1016/j.addma.2023 and 10.1007/BF02706350). These citations have been included in the revised manuscript to support our claim of accelerated cationic curing by hydroxy functionality.

New references on page 3 of the revised manuscript: "… and 2-hydroxyethyl acrylate (HEA, 12 w%) to accelerate the cationic curing[31,37,38]…"

5.It seems that the soluble thermoplastic part is transparent, while the crosslinked part has a yellow color. Why is that?

We appreciate this query by Reviewer 1. Although we do not know the exact origin of the color for parts exposed to UV light, we attribute it to a byproduct from photoacid generation. This is in-line with what has been observed for other cationic photopolymerizations. For example, Liska and coworkers reported that diphenyliodonium photoacid generators with different counteranions can produce cured parts with different extents of optical clarity and color (DOI: 10.1021/acsapm.2c01465). We thus view this as an opportunity going forward to identify multimaterial resin systems that can provide access to thermoset parts with different degrees of optical clarity and color.

6.The captions for Figure S36 and S37 are the same. Please confirm if S37 corresponds to photosystem 2?

We thank the reviewer for pointing out this error. Figure S37 (now Figure S40) corresponds to photosystem 2 and has been updated accordingly. Additionally, the wavelength was noted for both Figures S36 and S37 (now Figures S39 & S40, respectively) as being 365 nm.

Updated captions on page S38 of the revised manuscript:

Figure S39. Optical microscope images of the print containing pillars of varying size using photosystem 1 after 365 nm exposure. Image taken using top-lighting. Total print dimensions are 7.5 mm × 7.5 mm. Full print stitched using 5× magnification (A) and 10× magnification of 4×4 pixel pillars spaced 16 pixels apart (B).

Figure S39. Optical microscope images of the print containing pillars of varying size using photosystem 2 after 365 nm exposure. Image taken using top-lighting. Total print dimensions are 7.5 mm × 7.5 mm. Full print stitched using 5× magnification (A) and 10× magnification of 4×4 pixel pillars spaced 16 pixels apart (B).

Reviewer: 2

Recommendation: Publish in ACS Central Science after minor revisions noted.

*Comments:*In this manuscript, Page group developed a novel strategy to incorporate dissolvable supports in DLP printing. Here, they used a multi-color (or dual wavelength) DLP printer. By widely designing the chemistry and photoinitiators, they were able to use one wavelength (365nm) to cure the resin into a stiff thermoset, and use another wavelength (either 405nm or 460nm) to turn the resin into a relatively soft thermoplastics, which can be dissolve quickly at room temperature by ethyl acetate. With this capability, they presented some very interesting demonstrations, such as interlocked chains and ball and socket joint. Overall, this is an excellent work representing a breakthrough in DLP 3D printing.

We thank the Reviewer for their time in carefully reading the manuscript and for recommending publication in *ACS Central Science* after minor revisions. Below we address the individual concern raised by the Reviewer.

1. I only have one small concern. The printed thermoset after the solvent treatment (for removing the supports) and drying (100 °C for 24 hours) showed a significant drop in modulus (from 1.6GPa to 1.2GPa for photosystem 1). The authors attributed this to microcracks from swelling or microvoids during drying. However, this seems to be an arbitrary guess. Can the authors do an SEM to see if there are microcracks or microvoids? Also, can the authors measure the T_g and see if there is a Tg shift?

We thank the Reviewer for suggesting further characterization to support our hypothesis regarding the origin of differences observed in tensile properties between as printed green parts and dried structures. Following Reviewer 2's advice, we conducted additional experiments with SEM and DMA to assess morphological or thermomechanical differences of the thermoset parts after washing and drying. SEM was utilized to image cross-sectional areas of PS1 365 nm printed samples before and after washing+drying (100 °C for 24 hours). In support of our hypothesis we observed the formation of microvoids/cracks upon drying, which we attribute to 'sol' removal. Notably, at the same magnification 'green' samples appeared relatively uniform and dense (i.e, absent of voids). This visual difference is further

substantiated by DMA experiments of 'green' as printed samples which revealed an increase in T_g from 79 ± 1 to 104 ± 2 °C upon drying indicating that monomeric, oligomeric, or photosystem components are removed during the EA wash and drying process leading to this shift. This data has been included in the updated SI (sections S2.2 and S2.11) and referenced in the main manuscript with our discussion of the DMA results as well as for the modulus drop. The SI data and new manuscript text are included below for accessibility.

New text on page 4 of the revised manuscript: "glassy thermoplastic ($T_g \approx 60$ °C by dynamic mechanical analysis, Figs. S12-S14, Tables S10-12)"

New text on page 6 of the revised manuscript: "The decrease in stiffness and stress at break may arise from defect formation, such as microcracks from swelling or microvoids during drying (Fig. S28)."

New results on page S19 of the revised supporting information file:

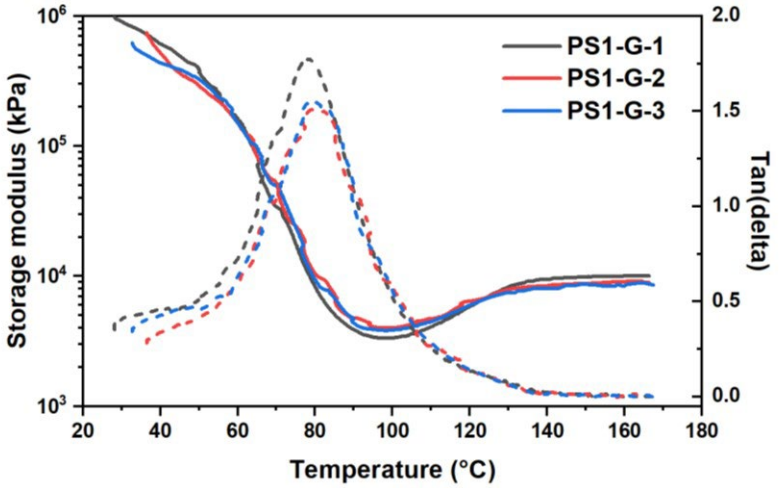

Figure S14. Dynamic mechanical analysis (DMA) of rectangle samples PS1-G-n (n = 1-3) as printed using 365 nm light (green bodies). Samples were lightly rinsed with isopropanol and air dried prior to testing.

Table S12. Glass transition temperature (T_g) of PS1 samples as printed using 365 nm light (green bodies), reported as the tan(delta) peak temperature in the DMA traces. The average value and standard deviation (STD) is also provided.

**Sample name**		**T_g (°C)**
**PS1 365 nm as printed**	**PS1-G-1**	78
	**PS1-G-2**	80
	**PS1-G-3**	80
	**Average**	**79 ± 1**

New results on page S31 of the revised supporting information file:

S2.11 SEM of Dried Samples

Following tensile testing where a drop in tensile modulus for thermoset parts was observed after washing and drying, SEM imaging of cross-sections was conducted. This revealed a morphological change with 'green' samples appearing more dense than corresponding washed and dried samples as indicated by the presence of voids throughout the polymer matrix (Fig. S28).

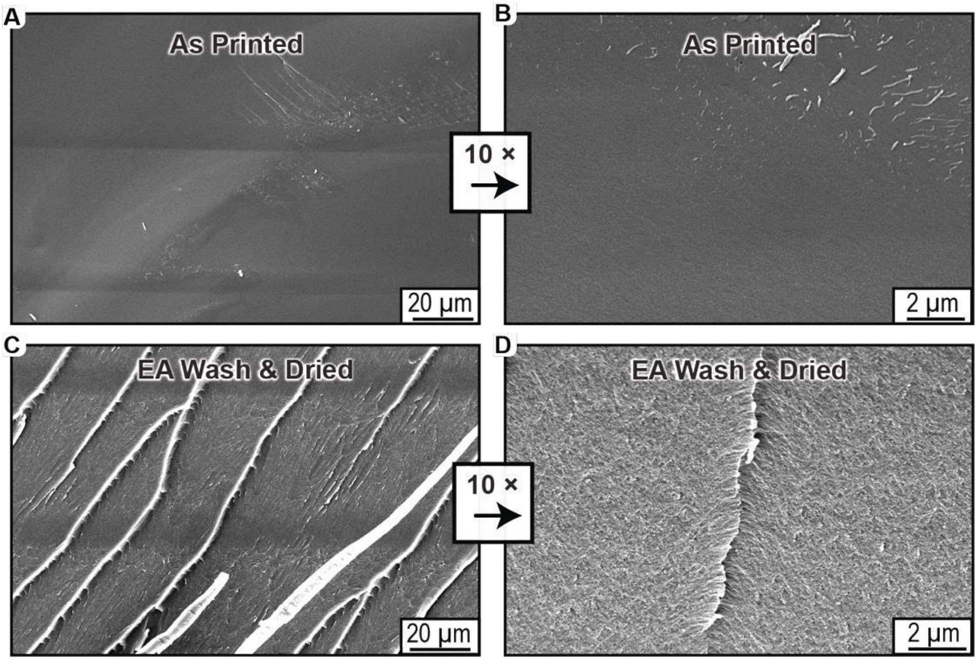

Figure S28. SEM images of sample cross-sections for 'green body' (as printed) and ethyl acetate washed (20 min.) and dried (100 °C in vacuo, 24 hours) samples that were prepared using 365 nm printing (photosystem 1). Notably changes in morphology from post-processing were observed. In both samples at ~2500× magnification, the print layer lines can be visualized by horizontal dark regions.
